# Exploratory Graph Analysis of the Strengths and Difficulties Questionnaire for Aboriginal and/or Torres Strait Islander Children

**DOI:** 10.3389/fpsyg.2021.573825

**Published:** 2021-08-18

**Authors:** Pedro Henrique Ribeiro Santiago, Davi Manzini Macedo, Dandara Haag, Rachel Roberts, Lisa Smithers, Joanne Hedges, Lisa Jamieson

**Affiliations:** ^1^Australia Research Centre for Population Oral Health, Adelaide Dental School, The University of Adelaide, Adelaide, SA, Australia; ^2^School of Public Health, The University of Adelaide, Adelaide, SA, Australia; ^3^School of Psychology, The University of Adelaide, Adelaide, SA, Australia; ^4^School of Health and Society, University of Wollongong, Wollongong, NSW, Australia

**Keywords:** exploratory graph analysis, network psychometrics, strengths and difficulties questionnaire, dimensionality, Aboriginal and/or Torres Strait Islanders

## Abstract

In Australia, one of the most frequently used measures for assessing social and emotional well-being (SEWB) of Aboriginal and/or Torres Strait Islander children is the Strengths and Difficulties Questionnaire (SDQ). Previous studies on state-level validations have indicated the problems associated with the original five-factor SDQ structure, especially in the dimension of Peer Problems. The aim of this study was to use a novel psychometric methodology, namely Exploratory Graph Analysis (EGA), to evaluate the dimensionality of caregiver-informant SDQ version 4–10 years at a national level in Australia. Data for this study were retrospectively collected from two independent longitudinal studies: the Longitudinal Study of Indigenous Children (LSIC) and South Australian Aboriginal Birth Cohort (SAABC). The caregiver-informed SDQ version 4–10 years was applied across several study waves, including more than 4,000 responses. To conduct EGA, Gaussian graphical models (GGMs) were estimated using the Least Absolute Shrinkage and Selection Operator. About 2,500 bootstrap samples were also employed to investigate dimensions and item stability. The findings indicated robust evidence against the construct validity of the original five-factor SDQ structure. Future studies should conduct a direct external validation of the findings with Aboriginal and/or Torres Strait Islander parents/carers and community groups to develop the guidelines for future use of the instrument among Aboriginal and/or Torres Strait Islander children in Australia.

## Introduction

In Australia, Aboriginal and/or Torres Strait Islander children disproportionally experience early developmental indicators that are poorer in performance compared to non-Aboriginal and/or Torres Strait Islander children (DSS, 2015; AIHW, 2018). A historical process of oppression and dispossession has placed Aboriginal and/or Torres Strait Islanders on the margins of Australian society, which impacts health and social and emotional well-being (SEWB) across generations (Paradies, 2016). Data collected from across Australia suggest that Aboriginal and/or Torres Strait Islander children aged 4–17 years are at a higher risk of clinically significant emotional and behavioral difficulties compared to their non-Indigenous peers (Zubrick et al., 2005; DSS, 2015). Mental-health-related conditions (e.g., depression, anxiety, alcohol misuse, self-inflicted injuries, and suicide) are the main contributors to the burden of disease for Aboriginal and/or Torres Strait Islander young people aged 10–24 years (AIHW, 2018). Thus, poor SEWB persists over time with deleterious effects on mental health. The design of early prevention and intervention strategies depends on the appropriate measurement of SEWB levels that consider an adaptation to specific sociocultural contexts (Williamson et al., 2014).

One of the most frequently used measures for assessing the SEWB of Aboriginal and/or Torres Strait Islander children is the Strengths and Difficulties Questionnaire (SDQ) (Zubrick et al., 2005; Williamson et al., 2014; DSS, 2015). SDQ is a 25-item behavioral screening instrument for children aged 4–17 years old, comprising five scales (each containing five items) named As “Hyperactivity,” “Emotional Symptoms,” “Conduct Problems,” “Peer Problems,” and “Prosocial” scales. Responses to the first four scales are summed to generate a total emotional and behavioral difficulty score (Goodman, 1997). Since its development, SDQ has been widely adopted in research and clinical practice and translated into more than 60 languages (Stone et al., 2010). Distinct SDQ versions have been developed to target specific age groups (2–4, 4–10, and 11–17 years) and different informants, such as the parent/carer, the teacher or a self-report by the child (Goodman et al., 1998).

### Psychometric Properties of SDQ

A systematic review conducted by Stone et al. (2010) indicated that there are strong SDQ psychometric properties, including sufficient convergent and discriminant validity, in addition to satisfactory internal consistency, test–retest reliability, and inter-rater agreement. The SDQ factorial structure, however, remains a topic of ongoing controversy (Goodman et al., 2010). There is a mixed support for the original five-factor structure originally proposed by Goodman (1997), and several studies have reported an unacceptable model fit, including the findings from a representative sample of children in Victoria, Australia (*n* = 914) (Mellor and Stokes, 2007). Mellor and Stokes (2007) concluded that the SDQ “factorial structure has not been adequately and appropriately confirmed,” and that “there might be limitations to the use of the instrument.” The authors also recommended that the “studies that ask whether or not it is feasible to find an alternative structure that would force a rearrangement of items onto alternative subscales are warranted” (Mellor and Stokes, 2007).

A recent review by Kersten et al. (2016) appraised the 17 studies that employed a confirmatory factor analysis (CFA) to evaluate the SDQ five-factor structure. In all studies, the fit was measured using the Root Mean Square Error of Approximation (RMSEA) and the Comparative Fit Index (CFI). The findings showed that while the RMSEA had acceptable values (RMSEA ≤0.7) (Steiger, 2007) for 12 out of 17 studies, only 7 studies had an acceptable CFI (CFI ≥0.95) (Kersten et al., 2016). The problems associated with the SDQ original five-factor structure has led researchers to seek alternatives. The most prominent one is a three-factor structure proposed by Dickey and Blumberg (2004) and Goodman et al. (2010), comprising the “prosocial,” “internalizing,” and “externalizing” scales, recommended for children with a low risk of psychosocial problems (Goodman et al., 2010).

### SQD Dimensionality for Aboriginal and/or Torres Strait Islander Children

The problems associated with the SDQ original five-factor structure among Aboriginal and/or Torres Strait Islander children have also been reported. Williamson et al. (2014) investigated the psychometric properties of the SDQ for 717 Aboriginal and/or Torres Strait Islander children in New South Wales, Australia. The findings indicated the problems associated with the “Peer Problems” scale as the items “picked on or bullied by other children” (*bullied*), “gets on better with adults than with other children” (*prefer adults*), and “rather solitary, tends to play alone” (*solitary*) displayed unacceptably low factor loadings (<0.40). The authors suggested that the “Peer Problems subscale does not appear to be completely appropriate for Aboriginal children.” Nonetheless, the removal of the Peer Problems scale altogether did not substantially improve a fit similar to the five-factor model (Williamson et al., 2014).

Another study by Zubrick et al. (2006) adapted SDQ for Aboriginal and/or Torres Strait Islander children and evaluated its properties in a large sample in Western Australia (*n* = 3,993). The adapted version slightly reworded the items to ensure cultural appropriateness and modified response categories (e.g., “sometimes” instead of “somewhat true”). Despite the overall good fit of the five-factor model, the study also reported the problems associated with the Peer Problems scale. The Peer Problems scale had the same two items, *bullied* and *prefer adults*, with unacceptable factor loadings (<0.40) and displayed inadequate reliability (<0.70). Zubrick et al. (2006) mentioned that the “predominant lack of fit in the factor analysis occurred with the *Peer Problems* factor.”

An additional concern is that, due to cultural differences, Western-developed instruments should not be assumed to be valid for Aboriginal and/or Torres Strait Islander (Santiago et al., 2019). For example, the problems of the Peer Problems scale potentially occurred as “Aboriginal parents did not conceptualize relationships with peers as the primary indicator of how well a child was able to operate interpersonally” (Williamson et al., 2014). Because Aboriginal and/or Torres Strait Islander societies historically comprise a kinship system, Aboriginal and/or Torres Strait Islander cultures place the value on connections to the extended family, to Elders and to the land. Thus, although the relationships of child with their peers are considered important, they may not have the same prominent role during child development in Aboriginal and/or Torres Strait Islander cultures as they do in Western societies. The SDQ focus on peer relationships fails to capture other more important relationships established by Aboriginal and/or Torres Strait Islander children, such as the connection to extended family and community (Thurber et al., 2019). In summary, it is possible that Aboriginal and/or Torres Strait Islander parents and/or caregivers potentially attributed a different meaning to the Peer Problems items. For an in-depth overview of previous validations of SDQ for Aboriginal and/or Torres Strait Islander children, please refer to Thurber et al. (2019).

### The Present Research

The rigorous measurement of SEWB is critical to the development and evaluation of prevention and treatment programmes for Aboriginal and/or Torres Strait Islander children (Williamson et al., 2014). The investigation of the SDQ dimensionality can inform whether alternative structures are required for non-Western cultures (such as Aboriginal and/or Torres Strait Islander), in which SEWB cannot be adequately described by “mental health” constructs and epistemologies (Nelson and Wilson, 2017). The present study aims to identify the dimensionality of the parent/carer-informed SDQ version 4–10 years (Goodman et al., 1998) for Aboriginal and/or Torres Strait Islander children. We have intended to address two research gaps. Firstly, we will investigate the SDQ dimensionality at a national level, using the data from the Longitudinal Study of Indigenous Children (LSIC), expanding from previous validation studies conducted at a state level in Western Australia and New South Wales (Zubrick et al., 2006; Williamson et al., 2014).

Secondly, we will employ a cutting-edge psychometric technique, namely exploratory graph analysis (EGA), to identify dimensionality (Golino and Epskamp, 2017). EGA is a technique within a broader framework of network psychometrics, a rapidly evolving field that investigates the associations between *behaviors* and *symptoms* instead of *constructs* or *domains* (Christensen et al., 2019). Simulation studies showed several advantages of EGA over traditional factor analytical and/or eigenvalue-based methods, such as principal component analysis (PCA) used in preceding studies (Zubrick et al., 2006; Thurber et al., 2019). For example, EGA outperforms traditional factor analytical methods in identifying the correct dimensionality when evaluating the instruments with multiple strongly correlated factors (as is the case of SDQ) or in large samples. In addition, EGA findings are displayed graphically (in a color-coded network plot), making the interpretation more intuitive (compared to a matrix of factor loadings) and more accessible to researchers, clinicians and policymakers without an expertise in psychological assessment. Another important advantage of EGA is that it reduces the “researcher degrees of freedom” as the algorithm automatically detects the instrument dimensionality, whereas factor analytical methods require the researcher to decide upon several statistical criteria, such as choosing between an enormous number of factor rotations to employ (Golino and Epskamp, 2017; Golino et al., 2020c). Finally, one fundamental advantage, which led EGA to receive scientific interest over the past years, is theoretical. EGA is compatible with the *network theory of mental disorders* (Borsboom, 2017) as it makes possible to evaluate dimensionality by inferring clusters of mutually reinforcing connected behaviors in a psychological network, in contrast to traditional factor analytical methods, which pose the existence of an unobservable latent variable causing these behaviors (Schmittmann et al., 2013). For an in-depth discussion of network theory and EGA, please see Christensen et al. (2020b).

To the best of our knowledge, prior to our research, only one study has employed network psychometrics to evaluate SDQ. Fonseca-Pedrero (2017) evaluated the self-report version of SDQ in a sample of 1,664 non-Indigenous students aged 14–19 years old in Spain. However, in that study, the dimensionality assessment (such as EGA) was not conducted.

## Methods

### Participants

The data collected from two independent longitudinal studies were analyzed. The first was the LSIC, which began in 2008 to investigate the developmental outcomes of Aboriginal and/or Torres Strait Islander children. Due to its accelerated cross-sequential design, LSIC follows two cohorts: the baby cohort (B Cohort) and the child cohort (K Cohort), respectively, aged 0.5–1.5 and 3.5–5 years at study baseline. Non-random purposive sampling was employed across 11 sites—from capital cities to remote communities—to ensure the inclusion of participants from different socioeconomic and geographic areas where Aboriginal and/or Torres Strait Islander children are likely to live. Participants were contacted using the recorded addresses provided by Centrelink and Medicare—Australian social welfare and healthcare services (Department of Families, 2009). The LSIC participants have been followed annually since 2008. In this study, we used the recent LSIC data available from baseline to Wave 10. Therefore, all waves containing the data from the caregiver-informed SDQ were included in the analysis: Wave 3, K Cohort (*n* = 502); Wave 4, K Cohort (*n* = 491); Wave 6, B and K Cohort (*n* = 1,072); Wave 8, B and K Cohort (*n* = 988), and Wave 10, B Cohort (*n* = 988).

In case of completion of the caregiver-informed SDQ version 4–10 years using both cohorts in the same wave (e.g., Wave 6 B Cohort and Wave 6 K Cohort), their information was combined into a single sample (i.e., Wave 6) during analysis. The samples from different waves were not combined as many of the children were re-evaluated throughout LSIC follow-ups and, consequently, the caregiver-informed SDQ item responses cannot be considered to be independent across waves. That is, the item responses by a caregiver regarding a single child are expected to be more similar across waves than in case of their reference to two completely distinct children. The children from the K Cohort were aged 4–8 years and 5–8 years old at Wave 3 and Wave 4, respectively. The children from the B Cohort were aged 8–10 years old at Wave 10. The children from the B and K Cohort were aged 4–10 years and 6–10 years old at Wave 6 and Wave 8, respectively. In all LSAC samples, the age of the participant children was within the age range covered by the caregiver-informed SDQ version 4–10 years, the version used in our analysis. In LSIC, the primary caregiver (i.e., informant) was defined as the parent who knows the study child best, in most cases “the child's biological mother but in some cases it was the child's father or another guardian” (Department of Social Services, 2019). The process to choose the type of psychological instruments to be included to measure the SEWB of Aboriginal and/or Torres Strait Islander children in LSIC (such as the caregiver-informed SDQ version 4–10 years) was conducted by Aboriginal and/or Torres Strait Islander researchers in partnership with Aboriginal and/or Torres Strait Islander stakeholders. A knowledge exchange session with the LSIC Research Administration Officers was conducted to learn about their previous experiences by administering specific psychological instruments to Aboriginal and/or Torres Strait Islander children and their views on the adequacy of these instruments. For more details on the selection of a psychological instrument in LSIC, please refer to Thurber et al. (2019).

An ethical approval for LSIC was received from the Australian Government Department of Health Departmental Ethics Committee and local Human Research Ethics Committees. All participants provided the signed informed consent. Prior to being interviewed for the first time at the study baseline, the parents were provided with an introductory letter and a DVD describing the LSIC study and the consent process. A plain language statement about the LSIC study and consent process was also available. The parents were informed that they could change their consent and/or withdraw from LSIC at any point of time. For the subsequent waves, participants were asked at each interview to confirm the consent that was previously given. For more information regarding the LSIC study consent process, please see the Department of Social Services (2019).

The second study was the South Australian Aboriginal Birth Cohort (SAABC) Study. At baseline, 449 mothers who were pregnant with an Aboriginal and/or Torres Strait Islander child throughout South Australia were recruited, which represented two-thirds of those who were eligible during the recruitment period. Follow-ups to document social, behavioral, cognitive, anthropometric, dietary and educational functioning of a child were conducted at a child aged 2 years (*n* = 324), 3 years (*n* = 324), and 5 years (*n* = 299). In this study, the sample comprised 250 Aboriginal and/or Torres Strait Islander children with valid caregiver-completed SDQ responses included in a 5-year follow-up study. The children from the SAABC in the 5-year study follow-up were aged 4–8 years old. The age of the participant children in the SAABC was within the age range covered by the caregiver-informed SDQ version 4–10 years, so the caregiver-informed SDQ version 4–10 years was the version indicated for this population and used in our analysis. In the SAABC, the primary caregiver (i.e., informant) was the biological mother of a child (Merrick et al., 2012). Following the recommended procedures in the cultural adaptation of instruments (Geisinger, 1994), the inclusion of the caregiver-informed SDQ version 4–10 years in the SAABC was decided in conjunction with the 15-member Aboriginal Reference Study Group, comprising Aboriginal community members, and Aboriginal infant care workers. Upon examination of the instrument, the Aboriginal Reference Group indicated that the caregiver-informed SDQ version 4–10 years could be useful to examine Aboriginal and/or Torres Strait Islander children SEWB, but an in-depth instrument evaluation was required. An ethical approval for this study was obtained from the University of Adelaide Human Research Ethics Committee and the Aboriginal Health Council of South Australia. All participants provided the signed informed consent. The consent was obtained using the NHMRC Guidelines for Ethical Conduct in Aboriginal and Torres Strait Islander Health Research (Merrick et al., 2012). Participants were informed at baseline that their participation was voluntary and they can refuse or withdraw at any stage without any reason or justification. All participants received a form explaining how to discuss their rights as a participant, raise concerns about the study and/or make complaints. At each study follow-up visit, the consent process was discussed again with participants, and participants were asked whether they wanted to maintain consent, change their consent and/or withdraw from the SAABC. Throughout the SAABC, the study results were also sent to the interested participants. For more information regarding the SAABC study consent process, please see Merrick et al. (2012). For general information about the SAABC, please refer to Jamieson et al. (2021).

In both LSIC and SAABC studies, information was only included for children aged 4–10 years at the time of completion of the caregiver-informed SDQ version 4–10 years. Throughout this study, the data analysis, the interpretation of the findings and the development of recommendations for future applications of the caregiver-informed SDQ version 4–10 years in Aboriginal and/or Torres Strait Islander children received oversight from and was developed in collaboration with the Deputy Director or Research and Senior Aboriginal Research Officer, Ms. Joanne Hedges, of the Indigenous Oral Health Unit (IOHU) at the University of Adelaide. In both the studies, all procedures were performed by following the ethical standards laid down by the 1964 Declaration of Helsinki and its later amendments.

### Measures

#### Strengths and Difficulties Questionnaire

Strengths and Difficulties Questionnaire is a 25-item brief behavioral screening tool that measures behaviors, emotions and relationships of children (Goodman, 1997). In this study, the SDQ version for children aged 4–10 years completed by the parent or primary caregiver was evaluated. Despite the information on the SDQ version 2–4 years being collected in certain LSIC waves, this study focused on the SDQ version 4–10 years as this version can be applied throughout a very long period of Aboriginal and/or Torres Strait Islander child development. Other versions, such as the version of 11–17 years (Goodman et al., 1998), are more commonly applied to evaluate adolescent well-being (Yao et al., 2009).

The SDQ version 4–10 years items were rated on a three-point scale (“not true,” “somewhat true,” and “certainly true”). As per the manual, 10 items were reverse-scored before analysis, so that higher scores indicated higher behavioral or emotional difficulties (Goodman et al., 2010).

### Statistical Analysis

The statistical analyses were conducted using the R software (R Core Team, 2013) and R packages *qgraph* (Epskamp et al., 2012) and *EGAnet* (Golino and Christensen, 2019). The R script used in this analysis is available in the Supplementary Material.

#### Exploratory Graph Analysis

The analysis was used to investigate the SDQ dimensionality (Golino and Epskamp, 2017). EGA is a technique within a broader framework of network psychometrics that employs the *walktrap algorithm* (Pons and Latapy, 2005) to identify clusters of items in a psychological network. In a psychological network, the nodes represent items and the edges represent associations between items (e.g., partial correlations). A *cluster* occurs when certain nodes are more strongly connected with each other compared to the rest of the network. Considering that these clusters generate the covariance patterns that are statistically equivalent to those produced using a latent variable (Kruis and Maris, 2016), any covariance matrix can be represented both as a network model and as a latent variable model (e.g., factor model), independently of the true data-generating mechanism (van Bork et al., 2019). Hence, EGA can be used to detect item clusters, which correspond to the dimensionality of the instrument. Simulation studies showed that EGA performs as accurate as traditional factor analytical techniques (e.g., Kaiser–Guttman eigenvalue >1 rule, screen test, parallel analysis) to identify dimensionality and outperforms them in large sample conditions (Golino et al., 2020c).

#### Network Model and Estimation

To conduct an EGA, it is necessary to first estimate the network model. In our study, network models were estimated for each sample (LSIC Wave 3K, LSIC Wave 4K, LSIC Wave 6, LSIC Wave 8, LSIC Wave 10B, and SAABC Wave 5). The network model used was the Gaussian graphical model (GGM), in which the nodes represent items and the edges represent partial correlation coefficients. In the GGM, the absence of an edge indicates conditional independence and the presence of an edge indicates conditional dependence (after conditioning on the entire set of variables) between items (Lauritzen, 1996). Moreover, partial correlations will rarely be exact zeros, so a penalized maximum likelihood (ML) estimation, the least absolute shrinkage and selection operator (LASSO) (Tibshirani, 1996), was used to avoid overfitting. The selection of the LASSO turning parameter was based on minimizing the extended Bayesian information criteria (EBIC) (Foygel and Drton, 2010). To ensure the robustness of the EGA results regarding the estimation method, sensitivity analysis was conducted by employing another estimation method, namely the triangulated maximally filtered graph (TMFG) approach (Massara et al., 2016). For instance, when both EGA and EGA with TMFG estimates find the same number of dimensions, it is likely that the optimal dimensionality solution has been found (Golino et al., 2020c).

An open question in network psychometrics is the handling of missing data and the performance of imputation methods (Santos et al., 2018), so we conducted all analysis using complete cases (that is, containing only participants who responded to all 25 SDQ items). The networks were plotted using the Fruchterman–Reingold algorithm (Fruchterman and Reingold, 1991), which plots the nodes according to the strength of their associations, arranging the nodes more closely with stronger associations.

#### Dimensionality and Item Stability

Exploratory graph analysis identifies the dimensionality specific to each sample. Therefore, to avoid an incorrect inference due to a sampling variation, we employed 2,500 bootstrap samples to evaluate the stability of the identified dimensions. We also evaluated *item stability*, which is the proportion of times the items clustered in their EGA-identified dimension (Christensen and Golino, 2019).

#### Network Loadings

After dimensionality was established, we calculated network loadings, which are the standardized sum of connections of each node within a particular dimension. Network loadings represent the contribution of each item (node) to the emergence of a coherent dimension (cluster) in a network (Christensen et al., 2020b). When the underlying causal structure is a common-cause model (e.g., factor model), network loadings provide equivalent information to factor loadings. However, under distinct causal structures (such as small-world networks), traditional factor loadings are inaccurate and network loadings should be preferred. Effect sizes for network loadings can be small (0.0–0.15), moderate (0.16–0.25), or large (0.26–0.35). Network loadings higher than 0.35 correspond to traditional factor loadings higher than 0.70. Network loadings can indicate the items that contribute to more than one dimension (i.e., cross-loadings) and also the items that are poorly related to any dimension (i.e., non-substantive network loadings; Christensen and Golino, 2021).

#### Model Fit

The evaluation of a model fit was done at two steps. Firstly, we evaluated the *relative fit* between dimensional structures to indicate which dimensional structure was more appropriate *compared* to the other SDQ structures. Secondly, we evaluated the *absolute fit* of each dimensional structure to indicate for each dimensional structure whether it correctly explained the observed SDQ item responses. That is, the absolute fit for each dimensional structure indicates “the *degree* of correspondence between the model and data” (Ribeiro Santiago et al., 2021b). The relative fit was evaluated by the Total Entropy Fit Index using Von Neumann entropy (TEFIvn) (Golino et al., 2020b). TEFIvn is an entropy fit index based on Von Neumann entropy, which was originally developed to measure quantum entanglement. Lower TEFIvn values indicate a better fit. The absolute fit was evaluated using three traditional fit measures in the factor analysis, Root Mean Squared Error of Approximation (RMSEA), Comparative Fit Index (CFI) and the Standardized Root Mean Square Residual (SRMR). The values of CFI ≥0.95, SRMR <0.08 and RMSEA ≤0.50 indicate a good absolute model fit (Kline, 2015), whereas RMSEA ≤0.70 indicates an acceptable absolute fit (Steiger, 2007).

To compare the fit of network models and factor models, we followed the recommendations from Kan et al. (2020). Considering that the SDQ items are polytomous ordinal items, network and factor models were both estimated based on the zero-order polychoric correlation matrix (Epskamp and Fried, 2018). While the use of zero-order polychoric correlation matrix is recommended for ordinal items, “polychoric inter-item correlation matrices that fail to be positive definitive are relatively common” (Lorenzo-Seva and Ferrando, 2021). In case of a non-positive definite correlation matrix, we used the Straight Smoothing algorithm (Bentler and Yuan, 2011) recommended by Lorenzo-Seva and Ferrando (2021). Kan et al. (2020) recommended that for both factor and network models, the absolute fit indices (i.e., RMSEA, CFI) should be calculated based on the discrepancy between the *zero-order correlation matrix implied by the factor or network model* and the *observed zero-order correlation matrix*. To highlight this correspondence (i.e., the fit of both network and factor models were calculated based on the same observed zero-order polychoric correlation matrix), we also reported the fit of the baseline model (Asparouhov and Muthén, 2010). The baseline model was specified by constraining covariances among the observed variables to zero while variances were freely estimated (Widaman and Thompson, 2003). Because the fit of the baseline model for both factor and network models is equal when based on the same observed zero-order correlation matrix, the baseline model χ^2^ should be similar across all models. Additionally, to enable a comparison with the network models, CFA models were estimated using the ML estimation. Considering the ordinal nature of the SDQ items, we also provide as means of sensitivity analyses factor models estimated with weighted least squares with a mean- and variance-adjusted (WLSMV) test statistic (Asparouhov and Muthén, 2010). The fit evaluation of factor and network models was conducted using R package *psychometrics* (Epskamp et al., 2020).

The evaluated *factor models* were: (1) the traditional SDQ five-factor structure (Goodman, 1997); (2) the three-factor structure (internalizing, externalizing and prosocial behavior) proposed by Dickey and Blumberg (2004) and Goodman et al. (2010); and (3) a proposed factorial structure based on the most common dimensions identified across all samples by EGA. The rationale of the proposed factorial structure is to inform whether there is any *common factorial structure* (distinct from the traditional SDQ five- and three-factor structures) that would be preferred and suitable for Aboriginal and/or Torres Strait Islander children across all samples. We evaluated the restricted factor models (i.e., items loaded only on the specified dimension and all cross-loadings set to zero) and the unrestricted factor models (i.e., cross-loadings allowed to be freely estimated; Marsh et al., 2014). To estimate the unrestricted models (Asparouhov and Muthén, 2009) using the R software, we followed the procedures described in Fischer and Karl (2019) and mentioned in detail in Silvestrin (2020). Because TEFIvn requires the partitioning of the items into dimensions (Golino et al., 2020b) to calculate TEFIvn for the unrestricted factor models, we partitioned the items into dimensions according to the highest factor loading. All factor models were estimated using the R package *lavaan* (Rosseel, 2012).

#### Item Redundancy

A psychological network is composed of autonomous causal components (Christensen et al., 2020b). However, the network requirement of autonomous causal components can be violated in the presence of latent confounding. Latent confounding occurs when a latent variable, which is not included in the network, has causal effects on two or more network components. The presence of latent confounding induces problems such as the failure to detect causal effects in the network and the lack of the interpretability of centrality measures (Hallquist et al., 2019). Latent confounding can occur in redundant items, such as the items so similar in content (e.g., “I like attending social events” and “I like going to parties”) that they are caused by a narrower characteristic (e.g., liking parties) of the original trait (e.g., extraversion) they intend to measure. In these cases, the redundant items will form “minor factors” instead of clustering with the other items measuring the broader trait (e.g., extraversion) and can lead dimensionality assessment methods such as EGA to *overfactor* (to identify more dimensions than in the true data-generating model) (Christensen et al., 2020a). Redundancy can also lead to structural inconsistency as the redundant items will cluster into minor factors in some samples but not in others (Christensen et al., 2020a).

To investigate whether the network components were unique or there were redundancies, we evaluated the weighted topological overlap (wTO) statistic (Zhang and Horvath, 2005) with an adaptive alpha (Pérez and Pericchi, 2014) following the recommendations by Christensen et al. (2020a). In case of the detection of redundancies between a set of items, instead of removing one or more redundant items, the set of redundant items was combined into a latent variable to avoid any loss of information. Items were combined into a latent variable only when, in addition to the items, exhibiting a strong and significant wTO, there was a theoretical justification for the observed redundancy. To evaluate the impact of redundancy on the SDQ dimensionality, we followed the subsequent steps: (1) we combined the pair of redundant items; (2) re-applied EGA to the new data with the combined items; and (3) re-applied a parametric bootstrap approach to evaluate structural consistency (Flores-Kanter et al., 2021).

#### Reliability

We investigated the reliability of the five-factor SDQ structure, three-factor structure and EGA-identified dimensions with the internal consistency reliability coefficient McDonald's Ω (McDonald, 2013). McDonald's Ω is recommended due to their several advantages over the traditional reliability index such as Cronbach's α, including that McDonald's Ω does not assume (1) tau-equivalence and a (2) congeneric model without correlated errors (i.e., locally independent items; Dunn et al., 2014). Reliability above 0.70 is adequate for research purposes (Furr and Bacharach, 2013). When the test scores are used to make decisions at an individual level, the internal consistency reliability of at least 0.80 or 0.85 is required for “lower-stakes standardized tests” while “high-stakes standardized tests should have internal consistency coefficients of at least 0.90” (Wells and Wollack, 2003).

## Results

The characteristics of participants are displayed in Table 1.

**Table 1:** Characteristics of study participants.

	**LSIC wave 3K**		**LSIC wave 4K**		**LSIC wave 6**		**LSIC wave 8**		**LSIC wave 10B**		**SAABC wave 5**	
	**(*****n*** **=** **519)**		**(*****n*** **=** **491)**		**(*****n*** **=** **1,072)**		**(*****n*** **=** **988)**		**(*****n*** **=** **727)**		**(*****n*** **=** **250)**	
	***n***	**%**	***n***	**%**	***n***	**%**	***n***	**%**	***n***	**%**	***n***	**%**
**AGE**												
Mean (SD)	5.6 (0.6)		6.6 (0.6)		6.8 (1.5)		8.2 (1.1)		9.6 (0.5)		5.6 (0.8)	
Min/Max	4–8		5–8		4–10		6–10		8–10		4–8	
Missing	0	0.0	0	0.0	0	0.0	0	0.0	0	0.0	10	4.0
**SEX**												
Female	257	49.5	242	50.7	554	51.7	498	50.4	373	51.3	111	44.4
Male	262	50.5	249	49.3	518	48.3	490	49.6	354	48.7	118	47.2
Missing	0	0.0	0	0.0	0	0.0	0	0.0	0	0.0	21	8.4

*Mean values, minimum, maximum, and SDs; numbers and percentages*.

For each sample, item and subscale scores are displayed in Supplementary Table 2. Before network estimation, the straight smoothing algorithm was applied to the SAABC Wave 5 polychoric correlation matrix as this polychoric correlation matrix was initially non-positive definite. On the other hand, all polychoric correlation matrices from LSIC were positive definite.

### Dimensionality

Exploratory graph analysis EGA indicated a three-dimensional structure in three samples (LSIC Wave 3K, LSIC Wave 8, and SAABC Wave 5), a four-dimensional structure in another two samples (LSIC Wave 4K and LSIC Wave 10B) and a five-dimensional structure in LSIC Wave 6. The EGA results and the percentages of dimensions identified in the bootstrap samples are displayed in Table 2.

**Table 2:** Number of Strengths and Difficulties Questionnaire (SDQ) dimensions identified by exploratory graph analysis (EGA).

	**LSIC**					**SAABC**
	**Wave 3 cohort K**	**Wave 4 cohort K**	**Wave 6 cohorts B and K**	**Wave 8 cohorts B and K**	**Wave 10 cohort B**	**Wave 5**
**Number of dimensions**	**3**	**4**	**5**	**3**	**4**	**3**
**Number of dimensions**	**Percentages (%)**	**Percentages (%)**	**Percentages (%)**	**Percentages (%)**	**Percentages (%)**	**Percentages (%)**
2	0.3	0.1	3.6	7.6	15.0	0.0
3	84.1	17.6	54.2	59.8	55.1	51.8
4	14.1	32.0	13.4	20.3	28.5	35.4
5	1.3	24.8	15.1	7.7	1.3	10.8
6	0.2	15.2	13.6	4.1	0.1	1.8
7	0.0	6.1	0.1	0.5	0.0	0.2
8	0.0	2.3	0.0	0.0	0.0	0.0
9	0.0	1.2	0.0	0.0	0.0	0.0
10	0.0	0.7	0.0	0.0	0.0	0.0

*The “study samples” row indicates the number of dimensions identified by EGA. The “bootstrap samples” rows indicate the percentage of times that each dimension was identified over the 2,500 bootstrap samples*.

The evaluation of the bootstrap samples showed that three- and four-dimensional structures were the most frequent in all study samples (the only exception being LSIC Wave 4K). The identification of three-dimensional structures ranged from 51.8% of all bootstrap samples from SAABC Wave 5 to 84.1% of all bootstrap samples from LSIC Wave 3K. The identification of four-dimensional structures ranged from 14.1% of all bootstrap samples from Wave 3K to 35.4% of all bootstrap samples from SAABC Wave 5. The five-dimensional structures were relatively less frequent, and their identification was ranged from 15.1% of all bootstrap samples from LSIC Wave 6 to 24.8% of all bootstrap samples from LSIC Wave 4K. Other structures, such as 2-dimensional, 6-dimensional, and 7-dimensional structures, and higher dimensional structures appeared more rarely.

The sensitivity analysis showed the number of identified dimensions using EGA and EGA with the TMFG estimation (Supplementary Table 3) concurred in LSIC Wave 3K (*n* = 3), LSIC Wave 8 (*n* = 3), and LSIC Wave 10B (*n* = 4). The evaluation of the EGA bootstrap samples after TMFG estimation showed that three- and four-dimensional structures were again the most frequent structures and support for the occurrence of five-dimensional structures in <1% of the bootstrap samples from all study samples in total.

### Item Stability

The item stability across all bootstrap samples is displayed in Table 3. Table 3 indicates the dimension to which each item belonged according to EGA and the proportion of times the clustering of each item with the *same* dimension. For example, Item 1 “Considerate of other people's feelings” (*considerate*) belonged to the dimension number three across all studies, clustering with items such as *shares, kind to kids, good friend*, etc. Please note that the dimension number is arbitrary and is used only to indicate which items are clustered together in a specific sample. Moreover, Item 1 was clustered with the same dimension (dimension number three) in 92% of all bootstrap samples in LSIC Wave 4K and up to 100% of all bootstrap samples in LSIC Wave 10B. The average stability of Item 1 was 97% since it clustered with the EGA identified dimension (dimension number three) in 97% of all bootstraps samples when the six study samples are considered. Table 3 also shows that Item 1 original SDQ dimension was “Prosocial Behavior.”

**Table 3:** Item stability of SDQ items.

	**Short name**	**LSIC**					**SAABC**	**Average stability (proportion)**	**Original dimension**
		**Wave 3K**	**Wave 4K**	**Wave 6**	**Wave 8**	**Wave 10B**	**Wave 5**		
Item 6	Solitary	2 (82%)	2 (24%)	1 (54%)	3 (91%)	1 (84%)	1 (83%)	70%	Peer problems
Item 19	Bullied	2 (42%)	1 (49%)	1 (40%)	1 (45%)	1 (98%)	1 (92%)	61%	Peer problems
Item 23	Prefer adults	1 (93%)	2 (23%)	1 (57%)	1 (60%)	1 (99%)	1 (93%)	71%	Peer problems
Item 5	Tempers	1 (94%)	1 (40%)	4 (88%)	1 (22%)	2 (18%)	1 (47%)	52%	Conduct problems
Item 12	Fights	1 (94%)	1 (47%)	4 (94%)	2 (92%)	1 (89%)	1 (56%)	79%	Conduct problems
Item 18	Lies	1 (94%)	1 (57%)	4 (94%)	2 (92%)	1 (98%)	1 (75%)	85%	Conduct problems
Item 22	Steals	1 (94%)	1 (57%)	4 (94%)	2 (92%)	1 (98%)	1 (75%)	85%	Conduct problems
Item 3	Somatic	2 (97%)	1 (42%)	1 (97%)	1 (92%)	2 (55%)	1 (94%)	79%	Emotional problems
Item 8	Worries	2 (99%)	1 (42%)	1 (97%)	1 (87%)	2 (55%)	1 (87%)	78%	Emotional problems
Item 13	Unhappy	2 (99%)	1 (42%)	1 (96%)	1 (80%)	2 (25%)	1 (91%)	72%	Emotional problems
Item 16	Clingy	2 (90%)	2 (55%)	1 (97%)	1 (82%)	2 (55%)	1 (75%)	76%	Emotional problems
Item 24	Fears	1 (99%)	1 (39%)	1 (97%)	1 (82%)	2 (55%)	1 (75%)	75%	Emotional problems
Item 2	Restless	1 (94%)	4 (99%)	2 (36%)	1 (21%)	4 (58%)	2 (98%)	68%	Hyperactivity
Item 10	Fidgety	3 (94%)	4 (99%)	2 (36%)	1 (22%)	4 (58%)	2 (98%)	68%	Hyperactivity
Item 15	Distractible	3 (93%)	4 (99%)	2 (28%)	1 (21%)	4 (58%)	2 (98%)	68%	Hyperactivity
Item 21	Reflective	2 (99%)	3 (98%)	5 (28%)	3 (96%)	3 (97%)	2 (68%)	81%	Hyperactivity
Item 25	Persistent	2 (99%)	3 (98%)	5 (30%)	3 (96%)	3 (97%)	3 (66%)	81%	Hyperactivity
Item 1	Considerate	3 (99%)	3 (92%)	3 (99%)	3 (99%)	3 (100%)	3 (99%)	97%	Prosocial behavior
Item 4	Shares	3 (99%)	3 (92%)	3 (95%)	3 (99%)	3 (100%)	3 (97%)	97%	Prosocial behavior
Item 20	Caring	3 (99%)	3 (92%)	3 (100%)	3 (99%)	3 (100%)	3 (99%)	98%	Prosocial behavior
Item 17	Kind to kids	3 (99%)	3 (92%)	3 (100%)	3 (99%)	3 (100%)	3 (98%)	98%	Prosocial behavior
Item 9	Helps out	3 (99%)	3 (94%)	3 (100%)	3 (100%)	3 (98%)	3 (84%)	96%	Prosocial behavior
Item 11	Good friend	3 (99%)	3 (92%)	3 (99%)	3 (95%)	3 (100%)	3 (99%)	97%	Peer problems
Item 14	Popular	3 (99%)	2 (7%)	3 (99%)	3 (99%)	3 (100%)	3 (99%)	84%	Peer problems
Item 7	Obedient	3 (95%)	3 (91%)	5 (30%)	3 (99%)	3 (97%)	3 (70%)	80%	Conduct problems

*The integers indicate which dimension identified by EGA the item belonged to. The percentages indicate the proportion of times the item clustered with the EGA-identified dimension over the 2,500 bootstrap samples. The colored boxed indicate a four-factor structure based on the most common dimensions identified across all samples*.

The items with lower stability were *tempers* (52%) from the Conduct Problems scale, *bullied* (61%), *solitary* (70%), and *prefer adults* (71%) from the Peer Problems scale and *restless* (68%), *fidgety* (68%), and *distractible* (68%) from the Hyperactivity scale. That is, these items were clustered with different dimensions across different bootstrap samples. The low stability of these items makes it harder for clear SDQ dimensions to be consistently identified as dimensions will be frequently composed of a different set of items. Thus, although three- and four-dimensional structures were the most frequently identified structures in our study (Table 2), the items belonging to each of these dimensions were different across the six samples (Table 3).

The items with higher stability were six items, the five items *considerate, shares, caring, kind to kids, helps out*, and *good friend* were from the Prosocial Behaviours scale and the item good friend from the Peer Problems scale. These six items achieved the value close to optimal 100% stability.

### Network Loadings

The investigation of network loadings indicated the problems associated with several items (Supplementary Tables 4–9). The item *solitary* had only small and/or non-substantive network loadings across all samples, indicating that this item is weakly contributing to the dimensions of the networks. For example, the *solitary* item had the network loadings of 0.06, 0.09 and 0.00 in the three dimensions identified by EGA in LSIC Wave 8. Other items, which consistently had only small and/or non-substantive network loadings, were *tempers* (LSIC Wave 3K, Wave 4K, and Wave 6) and *prefer adults* (LSIC Wave 4K, LSIC Wave 6, and LSIC Wave 8). The weak network loadings are possibly one of the reasons why *tempers* (52%)*, solitary* (70%), and *prefer adults* (71%) were among the items with the lowest stability as these items in most cases were not moderately or strongly associated with any dimension and, consequently, clustered with distinct dimensions across the study and bootstrap samples.

Moreover, in LSIC Wave 6, there was a high number of items with only small and/or non-substantive network loadings, such as *solitary, temps, prefer adults, helps, bullied*, and *obedient*. Once again, the weak network loadings are possibly related to the low stability of the items *solitary* (54%), *tempers* (88%), *prefer adults* (57%), *bullied* (40%), and *obedient* (30%) observed in LSIC Wave 6. In addition, the item persistent displayed cross-loading in LSIC Wave 6 as it had moderate network loadings to two dimensions.

### Proposed Factorial Structure

Considering that a unique dimensional structure was not found across the six samples (there was no “unique solution” for the SDQ dimensionality), we investigated based on the EGA results what was the most similar structure across all samples and whether this would be a suitable factorial structure for Aboriginal and/or Torres Strait Islander children aged 4–10 years. We identified a four-factor structure based on the EGA structures found across all six-study samples.

The inspection of the EGA results indicated that certain items were clustered together most of the time. For instance, one set of 10 items including *reflective, persistent, considerate, shares, caring, kind to kids, helps out, good friend, popular*, and *obedient* belonged to the same dimension (i.e., dimension number three) across all samples, with a few exceptions (e.g., *popular* on LSIC Wave 4K; Table 4). These items constituted the first dimension. Moreover, another set of 5 items containing *somatic, worries, unhappy, clingy* and *fears* also consistently clustered together with only two exceptions (*fears* on LSIC Wave 3K and *clingy* on LSIC Wave 4K) and constituted the second dimension. These five items correspond to the original SDQ “Emotional Problems” dimension.

**Table 4:** Model fit comparison of factor and network models of SDQ.

**Model**	**TEFIvn**	**χ^**2**^**	***df***	***p-*value**	**Baseline χ^**2**^**	**RMSEA**	**90% CI**	**CFI**	**SRMR**
**LSIC WAVE 3K**									
**Factor structures**									
5-factor structure	−19.072	2265.74	265	<0.001	5586.30	0.121	[0.116, 0.125]	0.622	0.126
5-factor structure (unrestricted)	−21.855	1083.25	185	<0.001	5586.30	0.097	[0.091, 0.102]	0.830	0.048
3-factor structure	−15.838	2927.74	272	<0.001	5586.30	0.137	[0.133, 0.142]	0.498	0.129
3-factor structure (unrestricted)	−22.628	1079.55	228	<0.001	5586.30	0.112	[0.107, 0.117]	0.720	0.060
4-factor structure (proposed)	−15.936	2023.77	269	<0.001	5586.30	0.112	[0.108, 0.117]	0.668	0.087
**Network structures**									
EGA-identified structure	−23.265	579.5	159	<0.001	5586.30	0.071	[0.065, 0.078]	0.920	–
**LSIC WAVE 4K**									
**Factor structures**									
5-factor structure	−19.822	2145.52	265	<0.001	4846.10	0.120	[0.116, 0.125]	0.586	0.099
5-factor structure (unrestricted)	–	–	–	–	–	–	–	–	–
3-factor structure	−16.025	2493.66	272	<0.001	4846.10	0.129	[0.124, 0.134]	0.511	0.110
3-factor structure (unrestricted)	−20.349	1756.49	228	<0.001	4846.10	0.117	[0.112, 0.122]	0.664	0.068
4-factor structure (proposed)	−16.821	2011.37	269	<0.001	4846.10	0.115	[0.110, 0.120]	0.617	0.085
**Network structures**									
EGA-identified structure	−20.618	257.90	113	<0.001	4846.10	0.051	[0.043, 0.059]	0.968	–
**LSIC WAVE 6**									
**Factor structures**									
5-factor structure	−18.671	2944.67	265	<0.001	10498.49	0.097	[0.094, 0.100]	0.737	0.083
5-factor structure (unrestricted)	−18.035	1611.62	185	<0.001	10498.49	0.085	[0.081, 0089]	0.860	0.038
3-factor structure	−17.340	3797.76	272	<0.001	10498.49	0.110	[0.107, 0.113]	0.654	0.093
3-factor structure (unrestricted)	−20.137	2384.93	228	<0.001	10498.49	0.094	[0.091, 0.097]	0.789	0.051
4-factor structure (proposed)	−14.964	2958.02	269	<0.001	10498.49	0.097	[0.093, 0.100]	0.736	0.077
**Network structures**									
EGA-identified structure	−18.829	593.33	141	<0.001	10498.49	0.055	[0.050, 0.059]	0.956	–
**LSIC WAVE 8**									
**Factor structures**									
5-factor structure	−19.435	3074.89	265	<0.001	9737.90	0.104	[0.100, 0.107]	0.702	0.097
5-factor structure (unrestricted)	−18.692	1507.65	185	<0.001	9737.90	0.085	[0.081, 0.089]	0.860	0.042
3-factor structure	−17.238	3937.24	272	<0.001	9737.90	0.117	[0.114, 0.120]	0.612	0.106
3-factor structure (unrestricted)	−20.603	2495.17	228	<0.001	9737.90	0.100	[0.097, 0.104]	0.760	0.057
4-factor structure (proposed)	−16.973	2888.03	269	<0.001	9737.90	0.099	[0.096, 0.103]	0.722	0.079
**Network structures**									
EGA-identified structure	−20.729	557.6	150	<0.001	9737.90	0.052	[0.048, 0.057]	0.957	–
**LSIC WAVE 10B**									
**Factor structures**									
5-factor structure	−17.912	3015.81	265	<0.001	9300.14	0.119	[0.116, 0.123]	0.694	0.126
5-factor structure (unrestricted)	−20.750	1266.99	185	<0.001	9300.14	0.090	[0.085, 0.094]	0.880	0.034
3-factor structure	−18.116	3787.19	272	<0.001	9300.14	0.133	[0.130, 0.137]	0.609	0.130
3-factor structure (unrestricted)	−22.271	2073.85	228	<0.001	9300.14	0.106	[0.101, 0.110]	0.759	0.053
4-factor structure (proposed)	−16.711	2455.24	269	<0.001	9300.14	0.106	[0.102, 0.110]	0.757	0.083
**Network structures**									
EGA-identified structure	−23.770	370.153	134	<0.001	9300.14	0.049	[0.043, 0.055]	0.974	–
**SAABC WAVE 5**									
**Factor structures**									
5-factor structure	−16.648	1505.48	265	<0.001	3944.88	0.137	[0.130, 0.144]	0.660	0.106
5-factor structure (unrestricted)	–	–	–	–	–	–	–	–	–
3-factor structure	−18.509	1709.66	272	<0.001	3944.88	0.145	[0.139, 0.152]	0.606	0.108
3-factor structure (unrestricted)	−21.743	1213.54	228	<0.001	3944.88	0.131	[0.124, 0.139]	0.730	0.062
4-factor structure (proposed)	−15.064	1504.11	269	<0.001	3944.88	0.136	[0.129, 0.142]	0.661	0.090
**Network structures**									
EGA-identified structure	−21.564	669.065	184	<0.001	3944.88	0.103	[0.094, 0.111]	0.867	–

*The original five-factor structure was suggested by Goodman et al. (1998). The three-factor structure was suggested by Goodman et al. (2010). The standardized root mean square residual (SRMR) was not available for the network models*.

The third dimension was composed of items *restless, fidgety* and *distractible*, which also clustered together across all samples with only one exception (*restless* on LSIC Wave 3K). Finally, the remaining seven items including *solitary, bullied, prefer adults, tempers, fights, lies*, and *steals* constituted the fourth dimension.

**Figure 1:**
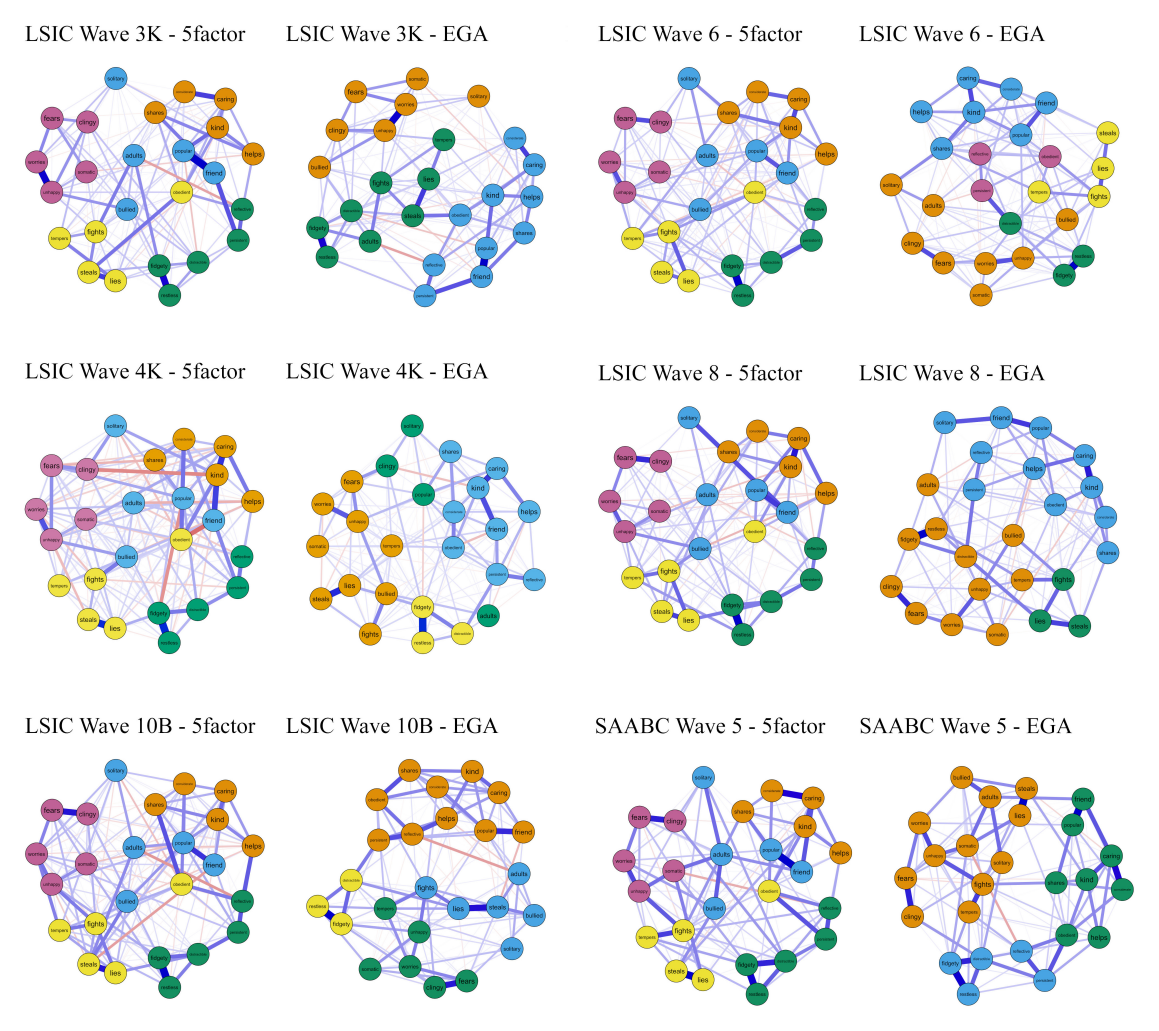
Networks of the caregiver-informant SDQ version 4 to 10 years in Aboriginal and/or Torres Strait Islander children. Note. Nodes were colored according to the SDQ five-factor structure (left column) and EGA identified structure (right column). SDQ, Strengths and Difficulties Questionnaire.

Figure 1 displays the estimated network with nodes colored according to the original five-factor SDQ structure (first and third columns) and the estimated network with nodes colored according to the dimensions identified by EGA (second and fourth columns).

For example, examining the SAABC Wave 5 in Figure 1, EGA indicated that the items *fidgety, distractible, restless, persistent*, and *reflective* were clustered together (third row fourth column, blue nodes) and this cluster was similar to their original SDQ dimension Hyperactivity (third row third column, green nodes). However, the items *solitary, adults, bullied, popular*, and *friends* clustered with two distinct dimensions (third row fourth column, orange and green nodes), differently from the original five-factor SDQ structure in which these items clustered together and constituted the Peer Problems dimension (third row third column, blue nodes). That is, a visual inspection of the networks provides further evidence that the SDQ items did not cluster according to the original five-dimensional structure. Notably, the Peer Problems items (first and third columns, blue nodes) were scattered across the networks and connected with other dimensions. Three- or four-dimensional structures (second and fourth columns) were identified by EGA in most samples.

### Model Fit

The model fit of the *factor models* [including the original five-factor structure (Goodman et al., 1998), the three-factor structure suggested by Dickey and Blumberg (2004) and Goodman et al. (2010) and the four-factor structure after rearranging the items based on EGA] and *network models* are displayed in Table 4.

The unrestricted five-factor model could not be estimated in LSIC Wave 4K and SAABC Wave 5 as convergence was not achieved. One problem with the unrestricted factor model is that, while they are preferred to the restricted factor model in the presence of cross-loadings, convergence cannot always be achieved (Lorenzo-Seva and Ferrando, 2021).

TEFIvn indicated that the network models had a better *relative fit* compared to the factor models across all samples, except for LSIC Wave 6 (the restricted three-factor structure was preferable) and SAABC Wave 5 (the unrestricted three-factor structure was preferable). However, when the *absolute fit* was evaluated, it was clear that all factor models had a poor fit while the network models had an acceptable or a good fit. For instance, the network models displayed acceptable (<0.07) or good (<0.05) RMSEA and CFI (>0.950) values across all samples. The only exception was LSIC Wave 3K (CFI = 0.920). The poor fit observed in SAABC Wave 5 of both factor and network models is potentially a result of the original polychoric correlation matrix being non-positive definite. While the application of a straight smoothing algorithm seems to improve the assessment of model-data fit, goodness-of-fit tests in a non-positive definite polychoric correlation matrix are potentially biased (Lorenzo-Seva and Ferrando, 2021). Therefore, the goodness-of-fit indices of the SAABC Wave 5K should be interpreted with caution. Finally, the fit of the factor models estimated using WLSMV was also poor (Supplementary Table 10). In the factor models estimated using WLSMV, while the RMSEA for the proposed four-factor structure was acceptable (<0.07), and the CFI was poor in all samples (<0.950). These findings indicate that the poor fit of factor models was robust across estimation routines. The parameters (factor loadings and factor correlations) of all factor models are displayed in Supplementary Tables 11–38. Considering the poor fit of the factor models (i.e., configural model), we did not proceed to test any further levels of measurement (and longitudinal) invariance such as *metric* or *scalar* invariance.

### Item Redundancy

In the SAABC Wave 5, based on the wTO statistic, the redundant pairs in a decreasing order of magnitude were: *restless-distractible* (wTO = 0.32), *restless-fidgety* (wTO = 0.31), and *lies-steals* (wTO = 0.28). After the pairs *restless-fidgety* and *lies-steals* were combined, EGA indicated a three-dimensional structure and an improvement in structural consistency as three-dimensional structures were identified in 65.7% of the bootstrap samples. In LSAC Wave 3K, the redundant pairs in a decreasing order of magnitude were: *worries-unhappy* (wTO = 0.34), *restless-fidgety* (wTO = 0.31), and *friend-popular* (wTO = 0.31). After the pair *restless-fidgety* was combined, EGA indicated a three-dimensional structure and high structural consistency as three-dimensional structures were identified in 95.7% of the bootstrap samples. In LSAC Wave 4K, the redundant pairs in a decreasing order of magnitude were: *restless-fidgety* (wTO = 0.31), *lies-steals* (wTO = 0.28), and *reflective-persistent* (wTO = 0.18). After the pairs *restless-fidgety* and *lies-steals* were combined, EGA indicated a two-dimensional structure. However, structural consistency remained low as three-dimensional structures were identified in 49.6% of the bootstrap samples.

In LSAC Wave 6, the redundant pairs in a decreasing order of magnitude were: *restless-fidgety* (wTO = 0.33), *caring-kind* (wTO = 0.22), and *clingy-fears* (wTO = 0.21). After the pair *restless-fidgety* was combined, EGA indicated a three-dimensional structure and structural consistency improved as three-dimensional structures were identified in 69.9% of the bootstrap samples. In LSAC Wave 8, the redundant pairs in a decreasing order of magnitude were: *restless-fidgety* (wTO = 0.31), *clingy-fears* (wTO = 0.26), and *caring-kind* (wTO = 0.22). After the pair *restless-fidgety* was combined, EGA indicated a three-dimensional structure and an improvement in structural consistency as three-dimensional structures were identified in 66.9% of the bootstrap samples. In LSAC Wave 10B, the redundant pairs in a decreasing order of magnitude were: *restless-fidgety* (wTO = 0.30), *clingy-fears* (wTO = 0.25), and *lies-steals* (wTO = 0.22). After the pairs *restless-fidgety* and *lies-steals* were combined, EGA indicated a two-dimensional structure and high structural consistency as two-dimensional structures were identified in 97.8% of the bootstrap samples.

### Reliability

The reliabilities of all SDQ subscales are displayed in Table 5.

**Table 5:** Reliability of the SDQ subscales.

**5-factor structure**	**Conduct problems**	**Hyperactivity**	**Peer problems**	**Emotional symptoms**	**Prosocial behaviors**
LSIC wave 3K	0.62 (0.56–0.68)	0.64 (0.58–0.69)	0.28 (0.17–0.39)	0.57 (0.50–0.65)	0.67 (0.60–0.73)
LSIC wave 4K	0.54 (0.46–0.61)	0.68 (0.63–0.73)	0.39 (0.28–0.49)	0.56 (0.49–0.62)	0.65 (0.57–0.72)
LSIC wave 6	0.68 (0.64–0.71)	0.71 (0.69–0.74)	0.41 (0.31–0.52)	0.61 (0.57–0.65)	0.61 (0.56–0.67)
LSIC wave 8	0.65 (0.61–0.69)	0.72 (0.69–0.74)	0.39 (0.30–0.49)	0.63 (0.59–0.67)	0.62 (0.57–0.66)
LSIC wave 10B	0.67 (0.63–0.71)	0.74 (0.71–0.78)	0.41 (0.33–0.49)	0.71 (0.67–0.75)	0.73 (0.68–0.77)
SAABC wave 5	0.68 (0.62–0.74)	0.84 (0.81–0.87)	0.62 (0.51–0.72)	0.68 (0.62–0.74)	0.75 (0.70–0.80)
**3-factor structure**	**Internalizing**	**Externalizing**	**Prosocial behaviors**		
LSIC wave 3K	0.62 (0.55–0.68)	0.71 (0.67–0.75)	0.67 (0.60–0.73)		
LSIC wave 4K	0.59 (0.53–0.66)	0.71 (0.66–0.75)	0.65 (0.57–0.72)		
LSIC wave 6	0.65 (0.62–0.68)	0.79 (0.77–0.81)	0.61 (0.56–0.67)		
LSIC wave 8	0.65 (0.62–0.69)	0.77 (0.75–0.79)	0.62 (0.57–0.66)		
LSIC wave 10B	0.72 (0.69–0.75)	0.80 (0.78–0.82)	0.73 (0.68–0.77)		
SAABC wave 5	0.76 (0.70–0.81)	0.68 (0.60–0.76)	0.75 (0.70–0.80)		
**Proposed 4-factor structure**	**Factor 1**	**Factor 2**	**Factor 3**	**Factor 4**	
LSIC wave 3K	0.76 (0.73–0.80)	0.57 (0.50–0.65)	0.68 (0.63–0.73)	0.61 (0.55–0.66)	
LSIC wave 4K	0.72 (0.67–0.75)	0.56 (0.49–0.62)	0.71 (0.67–0.76)	0.53 (0.46–0.60)	
LSIC wave 6	0.72 (0.69–0.76)	0.61 (0.57–0.65)	0.72 (0.69–0.75)	0.59 (0.55–0.54)	
LSIC wave 8	0.71 (0.68–0.75)	0.63 (0.59–0.67)	0.73 (0.70–0.77)	0.61 (0.56–0.65)	
LSIC wave 10B	0.81 (0.78–0.84)	0.71 (0.67–0.75)	0.77 (0.74–0.80)	0.68 (0.64–0.72)	
SAABC wave 5	0.83 (0.80–0.87)	0.68 (0.60–0.76)	0.83 (0.80–0.87)	0.71 (0.65–0.76)	

*The first section (first six rows) displays the reliability of the original five-factor SDQ structure. The second section displays the reliability of the SDQ three-factor structure. The third section displays the reliability of the four-factor structure based on the most commonly identified dimensions*.

The reliability of the theoretical five SDQ subscales was adequate only for the Hyperactivity subscale, which achieved the reliability higher than 0.70 in most samples (but not in LSIC Wave 3K and LSIC Wave 4K). The reliability of all other subscales (Conduct Problems, Peer Problems, Emotional Symptoms and Prosocial Behaviors) was poor (<0.70) with a few exceptions (emotional symptoms in LSIC Wave 10B and prosocial behaviors in LSIC Wave 10B and SAABC Wave 5).

In the three-factor SDQ structure, the externalizing subscale displayed adequate reliability (>0.70) across all samples (except for SAABC Wave 5). On the other hand, the internalizing subscale and prosocial behaviors subscale displayed poor reliability in most samples, displaying adequate reliability only in LSIC Wave 10B and SAABC Wave 5. Reliability of the proposed 4-dimensional structure was mixed, since Factor 1 and 3 displayed adequate reliability across most samples, while Factor 2 and 4 displayed poor reliability.

## Discussion

The present study aimed to identify the SDQ dimensionality for Aboriginal and/or Torres Strait Islander children aged 4–10 years in a large national sample and a smaller regional sample. We used a novel psychometric technique, EGA, to investigate the SDQ factorial structure in two independent studies, LSIC and SAABC. We also investigate whether a factor or a network structure would better explain the SDQ item responses for Aboriginal and/or Torres Strait Islander children.

Our findings indicated that five- or three-factor SDQ structures were not replicated and that a unique dimensionality across all samples could not be found. We proceeded then to investigate whether a factor model based on the *most common dimensions* identified across all samples could constitute an alternative structure suitable for Aboriginal and/or Torres Strait Islander children. However, a proposed four-factor structure (i.e., the most similar structure based on all samples) also displayed a poor fit and was not appropriate for Aboriginal and/or Torres Strait Islander children. The findings showed instead that, compared to a traditional factor structure, a network structure better explained the SDQ item responses for Aboriginal and/or Torres Strait Islander children. Implications for future applications of the caregiver-informed SDQ version age 4-10 years in Aboriginal and/or Torres Strait Islander children are provided below.

### Problems With the Peer Problems Scale

The SDQ dimensionality was not consistent, and different structures were found across the samples. One reason for the different structures was the poor stability of certain items, including *bullied, solitary*, and *prefer adults* from the Peer Problems scale. The problems with the Peer Problems scale have been extensively reported for non-Indigenous and Indigenous children. For instance, in non-Indigenous children, a systematic review by Kersten et al. (2016) showed that the Peer Problems scale had the worst internal consistency of all scales with a weighted average Cronbach's α of 0.49 (*SD* = 0.20) and displayed “unacceptable” convergent validity with other psychological measures (Kersten et al., 2016). Another systematic review by Stone et al. (2010) showed that the Peer Problems had poor discriminant validity with a weighted area under the curve (AUC) just above 0.5 and the ability of the scale “to distinguish between children with diagnoses, and those without it, is just above chance level.”

Problems with the Peer Problems scale were also reported for Aboriginal and/or Torres Strait Islander children in Australia. The same two items that showed low stability in our study, *bullied* and *prefer adults*, had unacceptably low factor loadings in the two previous validation studies (Zubrick et al., 2006; Williamson et al., 2014). In our findings, a visual inspection of the networks revealed that the items of Peer Problems did not cluster together but were scattered to combine with other dimensions. For instance, the items *bullied, prefer adults* and *solitary* clustered with items *tempers, fights, lies* and *steals* from the Conduct Problems scale, while the items *popular* and *good friend* (also from the Peer Problems scale) clustered with items such as *kind to kids* and *caring* from the Prosocial scale. These findings are consistent with Williamson et al. (2014), which reported that the Peer Problems scale was highly correlated with the Prosocial Behaviours scale in a sample of Aboriginal and/or Torres Strait Islander children living in New South Wales, suggesting that “aboriginal parents may think of ‘getting along well with others' as a single factor that incorporates the elements of both scales.” In summary, our findings provide further evidence the Peer Problems scale does not seem to capture a specific domain of the SEWB of Aboriginal and/or Torres Strait Islander children (Zubrick et al., 2006; Williamson et al., 2014).

### Comparison Between Factor and Network Models

A unique SDQ dimensionality could not be found across all samples. Our findings are in accordance with Williamson et al. (2014) that “the SDQ does not have a ‘clean' internal factorial structure” for Aboriginal and/or Torres Strait Islander children. Additionally, previous cross-cultural studies noted that the SDQ structure has been difficult to replicate and was not invariant across distinct ethnicities/cultural groups (Stevanovic et al., 2015). Stevanovic et al. (2015) discussed how “especially the items of the Peer Problems and Hyperactivity factors were perceived differently across the countries and they could be regarded as strongly influenced by specific factors—*culture-dependent* items.” Based on the findings from the current study, there was robust evidence against the construct validity of the original five-factor SDQ structure (or the three-factor structure) for Aboriginal and/or Torres Strait Islander children. These findings are consistent with a previous study by Williamson et al. (2014) that also raised concerns regarding the use of SDQ among Aboriginal and/or Torres Strait Islander children. Moreover, the inadequacy of Western-developed psychological instruments for Aboriginal and/or Torres Strait Islander populations and the need for culturally specific versions have been documented in instruments to measure personal control (Santiago et al., 2020a), stress (Santiago et al., 2019), among many others (Kowal et al., 2007).

Among our study samples, the most frequent structures were three- and four-dimensional. Considering that a unique solution could not be found, we investigated whether an alternative four-factor structure based on the most common dimensions identified across all samples would be adequate for Aboriginal and/or Torres Strait Islander children. However, the proposed four-factor structure also displayed an unacceptable fit. That is, we were unable to “find an alternative structure that would force a rearrangement of items onto alternative subscale” suitable for Aboriginal and/or Torres Strait Islander children (Mellor and Stokes, 2007).

There are several reasons why a unique dimensional structure was not found among Aboriginal and/or Torres Strait Islander children. Certain behaviors evaluated by SDQ in Aboriginal and/or Torres Strait Islander children such as *persistent* did not consistently cluster with a single dimension as they also established connections with behaviors from other clusters (i.e., network cross-loadings). Additionally, in many samples, behaviors such as *tempers, solitary*, and *prefer adults* were not strongly associated with any particular cluster (i.e., weak network loadings). Because these items did not consistently cluster with the same cluster of items (i.e., low item stability), multiple distinct SDQ dimensionalities were observed for Aboriginal and/or Torres Strait Islander Australian children across the LSIC/SAABC samples. In these cases, factor models, which require items to cluster exclusively with a specific dimension and to have no associations with other items given the dimension (i.e., local independence), will be inadequate and display a poor fit. This was observed in our study as all factor models displayed an unacceptable fit and only a network structure better explained the SDQ item responses.

The failure of factor models to explain the caregiver-informant SDQ item responses in Aboriginal and/or Torres Strait Islander children indicates that items did not consistently cluster into unique dimensions and, consequently, the summation of these items into a sum score representing a dimension (for example, sum score for the dimension “Hyperactivity”) is problematic. This phenomenon has been observed, for instance, regarding the items measuring depression (Fried et al., 2014). Fried et al. (2014) discussed how the calculation of subscale scores for items measuring major depressive disorder (MDD) “may obfuscate crucial information about the nature of depression symptoms and causes” as individual depression symptoms have distinct risk factors. For example, one main finding reported by Fried et al. (2014) was that women were more likely to report the worsening of sleep, whereas men were more likely to report increased suicidal ideation. Naively summing these items (i.e., sleep problems and suicidal ideation) into a sum score of depression, instead of evaluating these items individually, would conceal this information. Further evidence that the summation of the caregiver-informant SDQ items into subscale sum-scores is inappropriate for Aboriginal and/or Torres Strait Islander children is the low reliability observed in our study of all SDQ subscales (except the Hyperactivity scale in certain samples). The low reliability indicates that the subscale sum score is severely influenced by a measurement error and, consequently, clinical screening based on these subscale scores is subject to a strong misclassification (Charter and Feldt, 2001). Hence, due to low reliability, our evidence indicates that the use of the caregiver-informant SDQ subscale scores for clinical screening in which important decisions will be made regarding Aboriginal and/or Torres Strait Islander children should be discouraged (Charter, 2003).

Instead of a factor structure, our findings indicated that a network structure appropriately described the caregiver-informant SDQ items in the Aboriginal and/or Torres Strait Islander population. For instance, considering the “Conduct Problems” subscale, the assumption behind a (common cause) factor model is that a *latent trait* of “conduct problems” is responsible for the children to fight, lie, steal, lose temper, and disobey. On the other hand, a network model assumes the reciprocally reinforcing causal relations between the behaviors of fighting, lying, stealing, losing temper, and disobeying of children that can be *labeled* as “conduct problems” (Borsboom and Cramer, 2013). Network models seem particularly suitable to understand SEWB among Indigenous people as their well-being is holistic (Kendall et al., 2019) and influenced by a complex interplay of structural, contextual, and individual factors, such as colonization, historical trauma, resilience, and discrimination (Soares et al., 2020). For instance, previous studies have emphasized the importance of network models to describe the health of Aboriginal and/or Torres Strait Islander by comparing the unique causal components of a psychological network (nodes) and their associations (edges) with Aboriginal and/or Torres Strait Islanders most distinctive art style, *dot painting* (Soares et al., 2021).

Additionally, the use of network models (instead of factor model) to evaluate behaviours believed to constitute reciprocally reinforcing causal relations instead of factor models (that assumes a latent trait as the common cause of these behaviors) has recently generated debate in several psychological areas such as intelligence (Schmank et al., 2021), loneliness (Chvojková, 2021) or concentration, and empathy of children (Golino et al., 2021). For example, in the field of intelligence, Kan et al. (2020) and Schmank et al. (2021) showed that network models better explained item responses to intelligence tests than factor models and were more aligned with modern theories of intelligence, such as *mutualism* and *process overlap theory* (Kovacs and Conway, 2016). In summary, the use of network models and a comparison with latent variable models answer for calls that “matching theoretical and statistical models is necessary to bring data to bear on theories” in psychology (Fried, 2020).

Finally, a psychological network should be composed of autonomous causal components. However, our redundancy analysis indicated that not all network components were autonomous among Aboriginal and/or Torres Strait Islander children. For instance, the SDQ items *restless-fidgety* and *lies-steals* were redundant and needed to be combined. While redundancies between other pairs of items were found, we combined the items *restless-fidgety* (Percy et al., 2008; Van Roy et al., 2008; Sanne et al., 2009; van de Looij-Jansen et al., 2011; Ortuño-Sierra et al., 2015a,b; Bøe et al., 2016; Lehmann et al., 2017; Garrido et al., 2018; Keller and Langmeyer, 2019; McCarron et al., 2020) and *lies-steals* (McCarron et al., 2020) as the redundancies have a theoretical base, which is previously documented in the SDQ literature. For example, van de Looij-Jansen et al. (2011) showed that the items *restless* and *fidgety* form a “minor factor” termed as *restlessness* within the broader SDQ dimensions of hyperactivity. The redundancy of these items should be considered in future analysis using independent SDQ item responses from Aboriginal and/or Torres Strait Islander children.

### Implications for Practice

Considering that responses to the caregiver-informant SDQ for children aged 4–10 years have already been collected for Aboriginal and/or Torres Strait Islander children in national surveys (Department of Families, 2009) and longitudinal cohorts in Australia (Jamieson et al., 2021), one main implication of the network structure of the caregiver-informant SDQ in Aboriginal and/or Torres Strait Islander children is that, instead of summing items into subscale scores, the SDQ items should be considered individually. For example, the item *bullied* (“picked on or bullied by other children”) can be used to inform Aboriginal and/or Torres Strait Islander children's experience of victimization without needing to be summed with the other Peer Problems items to create a subscale score (Ribeiro Santiago et al., 2021a). Thus, based on our findings from more than 4,000 caregiver-informant SDQ responses, we do not recommend the summation of items into subscale scores (e.g., “Hyperactivity,” “Emotional Symptoms,” “Conduct Problems,” “Peer Problems,” and “Prosocial Behavior” subscales) for Aboriginal and/or Torres Strait Islander children. Moreover, due to the low reliability of the SDQ subscales indicating that the subscale sum scores are strongly influenced by a measurement error, we also discourage the use of the SDQ caregiver-informer subscales for clinical screening among Aboriginal and/or Torres Strait Islander children. We believe that additional improvements on the psychometric properties of SDQ do not implicate further rearrangement of items, and future research should treat the caregiver-informant SDQ items as independent variables (and removing them from the calculation of subscale sum scores) to measure these behaviors (e.g., *restless* or *popular*) among Aboriginal and/or Torres Strait Islander children.

Another important consideration is the development of culturally specific psychological instruments to measure SEWB among Aboriginal and/or Torres Strait Islander children. The need for culturally specific instruments has been recommended by many leading researchers in the field including Westerman (2002, 2004), Kowal et al. (2007), Brown et al. (2016), and Santiago et al. (2020a). One culturally appropriate instrument that has been recently proposed for Aboriginal and/or Torres Strait Islanders is the Strong Souls index (Thomas et al., 2010). The Strong Souls was originally developed for Aboriginal and/or Torres Strait Islanders adolescents aged 16–21 years based on an ongoing consultative process with Aboriginal community members and mental health experts (Thomas et al., 2010; Thurber et al., 2019). As pointed out by Thurber et al. (2019), the instrument was named as Strong Souls “in recognition that the concept of ‘soul' encompasses the physical, emotional, social and spiritual being of a person and was therefore synonymous with SEWB” (Thomas et al., 2010). In the last few years, the instrument has been refined and have recently been applied for the first time to children aged 11–13 years in LSIC (Thurber et al., 2019). The instrument has also been applied in the SAABC 9-years-old follow-up (Jamieson et al., 2021). The psychometric properties of Strong Souls' have yet to be systematically assessed among Aboriginal and/or Torres Strait Islander children, and this is an agenda for future research.

### Theoretical Contributions and Limitations

The current study provided the most comprehensive examination of the SDQ dimensionality among Aboriginal and/or Torres Strait Islander Australians. We investigated the dimensionality using a national and a regional sample from two independent longitudinal studies, across several waves, including more than 4,000 responses to the caregiver-informed SDQ version 4–10 years. We replicated the previous findings by Williamson et al. (2014) suggesting the original five-factor SDQ structure does not seem to be entirely adequate for Aboriginal and/or Torres Strait Islander Australians and Zubrick et al. (2006) who also reported the problems with the Peer Problems factor. Hence, the findings from the current study provide evidence against the construct validity of the original five-factor structure for the caregiver-informed SDQ version 4–10 years for Aboriginal and/or Torres Strait Islander Australian children. The network analysis provided new insights into the SDQ functioning, such as how the Peer Problems items were scattered across the network clustering with other dimensions and how a network structure should be preferred than a factorial structure for Aboriginal and/or Torres Strait Islander children. While (Ribeiro Santiago et al., 2021a) recently compared factorial structures identified from EGA with traditional SDQ factorial structures in Australian children, to the best of our knowledge, this is the first study to directly compare network structures with traditional SDQ factorial structures across any population. While network models have been recently shown to be superior to factor models when evaluating intelligence (Schmank et al., 2021) and even the concentration and empathy of children (Golino et al., 2021), to the best of our knowledge, this is also the first study to compare network and factor models to evaluate the SEWB of children. Finally, these results also expand beyond Aboriginal and/or Torres Strait Islander children by suggesting that the construct validity of the five-factor SDQ structure for non-Western cultures is not given, especially for Indigenous groups in which the western concepts of “mental health” are a poor representation of SEWB (Nelson and Wilson, 2017).

There were several limitations to the current study. One limitation was that, due to the longitudinal nature of the data, the LSIC waves were not independent. Hence, it is possible that, despite the differences due to child development, the network structure remained more consistent across LSIC waves than in case of considering completely independent samples. While we evaluated in our study *cross-sectional psychological networks* using distinct waves of longitudinal studies, methods have also been recently proposed to estimate *longitudinal psychological networks* using longitudinal or time-series data (McNally, 2020). Recent methodological implementations include multilevel vector autoregressive (MVAR) models (Bringmann et al., 2015), impulse-response function (Bos et al., 2017), and dynamic EGA (Golino et al., 2020a). However, these methods are still under development and were not implemented in the current study due to this reason.

The second limitation was that we did not consider measurement (and longitudinal) invariance among the network models. The investigation of measurement invariance is important as LSIC and SAABC comprised children with distinct characteristics, such as children belonging to different gender and age groups and/or residing in different regions. Moreover, Aboriginal and/or Torres Strait Islanders comprise a highly culturally heterogeneous group (Hunt, 2013), a heterogeneity referred by the term “Aboriginal cultures” (Reay, 1988). Thus, while we evaluated the responses of the caregiver-informant SDQ networks for children from each study wave, future studies should evaluate whether there were differences based on the children characteristics (e.g., gender, age, region, and cultural group). In terms of the factor models evaluated in our study, as the factor models had a poor fit and the factorial structures were different across samples (i.e., configural invariance was not achieved), there was no reason to evaluate further levels of measurement invariance, such as metric or scalar invariance (Meredith, 1993). Notwithstanding, future research should evaluate *measurement invariance among the network models* to indicate whether the caregiver-informant SDQ networks in Aboriginal and/or Torres Strait Islander children differ according to characteristics (e.g., gender and region) and/or remain stable across distinct ages (i.e., longitudinal invariance). The evaluation of network loadings *metric invariance* is theoretically possible (Christensen et al., 2020b); however, these methods are currently under development and are still not available in statistical software.

The third limitation was that the fit of both factor models (restricted and unrestricted) were examined in the same sample that the models were estimated and can potentially be overestimated (Fokkema and Greiff, 2017). However, we decided not to split the samples into training and cross-validation data sets as the LSIC and SAABC samples were of medium size. That is, we aimed to retain the maximum statistical power for model estimation, especially considering that we estimated complex models such as unrestricted factor models (Asparouhov and Muthén, 2009). The same consideration applies to the reason of initially not dividing our sample according to distinct characteristics of children (e.g., the region of living and gender) and evaluate the entire sample. Finally, despite these considerations, our study had a substantive sample size compared to the other studies validating the instruments for First Nation people (McCuish et al., 2018), especially considering several challenges in recruiting minority populations such as First Nation people (Mhurchu et al., 2009; Fox et al., 2010).

One point that needs to be considered when interpreting the findings is the representativeness and two potential sources of bias in the two longitudinal cohorts. Firstly, because the LSIC sampling strategy was non-random purposive sampling, the LSIC sample is not nationally representative. At baseline, LSIC comprised 5–10% of all Aboriginal and/or Torres Strait Islander children in their respective age and sufficiently reflected the distribution of Aboriginal and/or Torres Strait Islander aged between 0 and 5 years at study baseline, considering states and territories among urban, regional and remote areas. In the follow-ups, the LSIC retention rates (i.e., counting the percentage of respondents from the previous waves successfully reinterviewed) ranged from 85.9% at Wave 2 to 87.9% at Wave 10. Some families could not be reinterviewed as “they could not be located, had moved substantial distances, refused interviews, or could not be interviewed for other reasons” (Department of Social Services, 2019). In the SAABC, the initial sample includes two-thirds of participants eligible for the study and was representative of age and socioeconomic position in South Australia (Jamieson et al., 2018). In the 5-year follow-up, the SAABC retained 68.6% of the eligible participants from baseline (Jamieson et al., 2019). In both longitudinal studies, it is possible that participants who could not be followed were not missed completely at random and were different from the participants that were followed. These differences can lead to distorted caregiver-informant SDQ scores in case of the children with no follow-up having worst (or best) behavioral or emotional difficulties compared to the children with follow-ups. While representativeness is desirable to calculate item means and develop population norms, the lack of representativeness does not entail that model (item) parameters are biased (Richiardi et al., 2013; Santiago et al., 2020b). Secondly, there are also possible concerns regarding self-selection bias as, in both studies, families needed to agree to participate in the study (Dodson et al., 2012). Once again, it is possible that Aboriginal and/or Torres Strait Islander children from the families who did not enroll in the studies had better (or worse) behavioral or emotional difficulties. Future studies should investigate the caregiver-informed SDQ psychometric properties for Aboriginal and/or Torres Strait Islander children in other samples to provide further evidence on our findings.

Another important point is that, while our findings provided robust evidence against the construct validity of the five-factor SDQ structure derived in Western children to Aboriginal and/or Torres Strait Islander children, we did not directly compare Aboriginal and/or Torres Strait Islander and non-Aboriginal and/or Torres Strait Islander Australian children to evaluate *cross-cultural validity* in terms of the caregiver-informed SDQ scores. Cross-cultural validation would be required for the *comparison* of scores between Aboriginal and/or Torres Strait Islander and non-Aboriginal and/or Torres Strait Islander children. Previous studies have demonstrated that *cross-cultural validity* between Aboriginal and/or Torres Strait Islander and non-Aboriginal and/or Torres Strait Islander children was not achieved for certain instruments, such as the Sense of Personal Control scale, posing challenges to a direct comparison of the test score between these groups (Santiago et al., 2020a). While conducting a cross-cultural validation of the caregiver-informed SDQ version 4–10 years between Aboriginal and/or Torres Strait Islander and non-Aboriginal and/or Torres Strait Islander Australian children was beyond the scope of this study, this is another agenda for future research.

Finally, one main limitation of our study is that, while the interpretation of findings and proposed guidelines received oversight from and were developed in collaboration with the Deputy Director or Research and Senior Aboriginal Research Officer at the IOHU, we did not conduct a direct external validation of the findings with Aboriginal and/or Torres Strait Islander parents/carers and community groups. Future qualitative studies should directly validate these findings with Aboriginal and/or Torres Strait Islander parents/carers and community groups to hear their perspective on the findings suggesting that traditional caregiver-informant SDQ dimensions (e.g., “Hyperactivity,” “Emotional Symptoms,” “Conduct Problems,” “Peer Problems,” and “Prosocial behaviors”) were insufficient to describe the SEWB of their Aboriginal and/or Torres Strait Islander children. Subsequent studies together with Aboriginal and/or Torres Strait Islander parents/carers and communities will provide further guidance regarding the application of SDQ among Aboriginal and/or Torres Strait Islander children and inform how the SDQ test scores should (or not) be used and interpreted in the future in Australia.

## Conclusions

Strengths and Difficulties Questionnaire is one of the main instruments used to evaluate the social and emotional well-being of Aboriginal and/or Torres Strait Islander children. The rigorous measurement of the SEWB in Aboriginal and/or Torres Strait Islander children is fundamental to the development and evaluation of prevention and treatment programmes. We conducted the largest evaluation to date using two independent longitudinal studies. The findings indicated robust evidence against the construct validity of the original five-factor SDQ structure (or the three-factor structure) for Aboriginal and/or Torres Strait Islander children. We recommend that item summation into subscale scores should be discouraged (and clinical screening based on subscale scores) and item information should be considered individually in future studies using the caregiver-informant SDQ in Aboriginal and/or Torres Strait Islander children. These findings, however, need to undergo an external validation with Aboriginal and/or Torres Strait Islander parents/carers and community groups to provide further and definitive recommendations regarding the use of caregiver-informed SDQ version 4–10 years among Aboriginal and/or Torres Strait Islander children. Finally, a future research agenda concerns the development and the investigation of culturally appropriate measure for measuring SEWB among Aboriginal and/or Torres Strait Islander children.

## Data Availability Statement

The data analyzed in this study was subject to the following licenses/restrictions: data from the Longitudinal Study of Indigenous Children (LSIC) are owned by a third party, the Australian Government Department of Social Services. There are security and confidentiality protocols for accessing LSIC data. Interested parties must submit an application and sign a deed of license. Information can be found on the LSIC webpage: http://www.dss.gov.au/lsic. Data from the South Australian Aboriginal Birth Cohort cannot be shared publicly because of its sensitive nature. The study participants constituted a significant proportion of the Aboriginal and Torres Strait Islander community in South Australia and the release of data could lead to the participants' identification. Data are available from the Aboriginal Research Advisory Committee of the Indigenous Oral Health Unit (Email: iohu@adelaide.edu.au. Phone: +61 8 8313 4611) for researchers who meet the criteria for access to confidential data. Requests to access these datasets should be directed to Aboriginal Research Advisory Committee of the Indigenous Oral Health Unit (Email: iohu@adelaide.edu.au. Phone: +61 8 8313 4611).

## Ethics Statement

Ethical approval for the LSIC was received from the Australian Government Department of Health Departmental Ethics Committee and local Human Research Ethics Committees. Ethical approval for the SAABC was obtained from the University of Adelaide Human Research Ethics Committee and the Aboriginal Health Council of South Australia. Written informed consent to participate in this study 1898 was provided by the participants' legal guardian/next of kin.

## Author Contributions

PS, DM, RR, LS, and LJ conceptualized the project. PS conducted the formal analysis. LJ provided resources and funding acquisition. PS, DM, and DH interpreted the results. PS and DM wrote the initial version of the manuscript. DH, LS, RR, JH, and LJ provided theoretical and statistical supervision. All authors reviewed and edited the final draft.

## Conflict of Interest

The authors declare that the research was conducted in the absence of any commercial or financial relationships that could be construed as a potential conflict of interest.

## Publisher's Note

All claims expressed in this article are solely those of the authors and do not necessarily represent those of their affiliated organizations, or those of the publisher, the editors and the reviewers. Any product that may be evaluated in this article, or claim that may be made by its manufacturer, is not guaranteed or endorsed by the publisher.
