# Erratum: Exploratory Graph Analysis of the Strengths and Difficulties Questionnaire for Aboriginal and/or Torres Strait Islander Children

**DOI:** 10.3389/fpsyg.2021.777519

**Published:** 2021-10-12

**Authors:** Frontiers Production Office

**Affiliations:** Frontiers Media SA, Lausanne, Switzerland

**Keywords:** exploratory graph analysis, network psychometrics, strengths and difficulties questionnaire, dimensionality, Aboriginal and/or Torres Strait Islanders

Due to a production error, a line number was inserted in the text; moreover, some terms in the text had their first letters wrongly uncapitalized; this has now been corrected and the original article has been updated. The publisher apologizes for this mistake.

The original article has been updated.

