# Supplementary material for: Exploratory Graph Analysis of the Strengths and Difficulties Questionnaire for Aboriginal and/or Torres Strait Islander Children
Source: Front Psychol. 2021 Aug 18;12:573825. doi: 10.3389/fpsyg.2021.573825 (PMC8416422; doi:10.3389/fpsyg.2021.573825)
Supplement: Supplementary file 1 [file Table_1.DOCX]

##############################################################################################################################

# Network analysis of the Strength and Difficulties Questionnaire among Aboriginal and/or Torres Strait Islander children #

# Pedro Henrique Ribeiro Santiago ######

# Davi Manzini Macedo ######

# Dandara Haag ######

# Rachel Roberts ######

# Lisa Gaye Smithers ######

# Jo-Hedges ######

# Lisa Jamieson ######

# 01-07-2021 ######

##############################################################################################################################

### Loading required R packages ###

library(NetworkToolbox)

library(qgraph)

library(foreign)

library(readxl)

library(psych)

library(bootnet)

library(EGAnet)

library(igraph)

library(ape)

library(pegas)

library(tnet)

library(psycho)

library(Rgraphviz)

library(haven)

library(readxl)

library(lavaan)

library(networktools)

library(plyr)

library(dplyr)

library(data.table)

library(psychonetrics)

library(tidyverse)

library(semTable)

library(fungible)

#######################################################

### SOUTH AUSTRALIAN ABORIGINAL BIRTH COHORT STUDY ###

#######################################################

### Opening the dataset ###

dataset <- read_excel("BTT_5yrs_SDQ.xlsx")

newdata <- cbind(dataset[,2], dataset[,5], dataset[,10], dataset[,18], dataset[,21],

dataset[,7], dataset[,12], dataset[,15], dataset[,20], dataset[,24],

dataset[,3], dataset[,11], dataset[,16], dataset[,22], dataset[,26],

dataset[,6], dataset[,8], dataset[,13], dataset[,19], dataset[,23],

dataset[,4], dataset[,9], dataset[,14], dataset[,17], dataset[,25])

names(newdata) <- c("Item_1", "Item_4", "Item_9", "Item_17", "Item_20",

"Item_6", "Item_11", "Item_14", "Item_19", "Item_23",

"Item_2", "Item_10", "Item_15", "Item_21", "Item_25",

"Item_5", "Item_7", "Item_12", "Item_18", "Item_22",

"Item_3", "Item_8", "Item_13", "Item_16", "Item_24")

newdata[newdata == 6]<- NA

keysSDQ <- c(-1, -1, -1, -1, -1,

1, -1, -1, 1, 1,

1, 1, 1, -1, -1,

1, -1, 1, 1, 1,

1, 1, 1, 1, 1)

newdata <- reverse.code(keysSDQ, newdata)

newdata <- as.data.frame(newdata)

newdatasetcc <- newdata[complete.cases(newdata),]

newdataset <- newdatasetcc

names(newdataset) <- c("considerate", "shares", "caring", "kind", "helps",

"solitary", "friend", "popular", "bullied", "adults",

"restless", "fidgety", "distractible", "reflective", "persistent",

"tempers", "obedient", "fights", "lies", "steals",

"somatic", "worries", "unhappy", "clingy", "fears")

##############################

### Preparing for analysis ###

##############################

CorMat <- qgraph::cor_auto(newdataset[,1:25],forcePD = TRUE)

fivescales <- list("Prosocial" = c(1:5), "Hyper"=c(6:10), "Emotion" = c(11:15),

"Conduct"=c(16:20), "Peer"=c(21:25))

CorFin <- CorMat

CorFin2 <- qgraph::cor_auto(newdataset[,1:25])

CorFin2smooth <- smoothBY(CorFin2)$RBY

########################

### Network Analysis ###

########################

#Network estimation and plot#

network1 <- EBICglasso(CorFin, n=nrow(newdataset))

png("networkBTT.png", res=300, height=5000, width=5000)

networkplot <- qgraph(network1, layout="spring", cut=0,

theme="colorblind", vsize=7.5, esize=18,

legend=FALSE, groups=fivescales,

labels=colnames(newdataset[1:25]))

dev.off()

#Dimensionality#

ndim <- EGAnet::EGA(CorFin, n=nrow(newdataset))

bootdimen <- EGAnet::bootEGA(newdataset, model="glasso", n=2500, ncores=2)

bootdimen$summary.table

bootdimen$frequency

dimstab <- dimStability(bootdimen, orig.wc =ndim$wc)

png("networkBTTEGA.png", res=300, height=5000, width=5000)

ndimplot <- plot(ndim, theme="colorblind", vsize=7.5, esize=18, layout="spring", legend=FALSE,

cut=0, labels=colnames(newdataset[1:25]))

dev.off()

###########################

### REDUNDANCY ANALYSIS ###

###########################

# Round 1 #

redSAABC <- EGAnet::UVA(newdataset[,1:25], method="wTO", type="adapt", reduce=FALSE,

reduce.method="latent", adhoc=FALSE)

redSAABC$redundancy$descriptives$centralTendency

redSAABC <- EGAnet::UVA(newdataset[,1:25], method="wTO", type="adapt", reduce=TRUE,

reduce.method="latent", adhoc=TRUE)

egaredSAABC <- EGA(redSAABC$reduced$data, algorithm = "walktrap")

bootdimenLSACegaredSAABC <- EGAnet::bootEGA(redSAABC$reduced$data, model="glasso", n=2500, ncores=2) #bootstraps the number of dimensions

bootdimenLSACegaredSAABC$summary.table #Summary table

bootdimenLSACegaredSAABC$frequency #Frequency dimensions

###################################

### DETERMINE FACTOR OR NETWORK ###

###################################

tradSDQ <- 'pro =~ considerate + shares + caring + kind + helps

peer =~ solitary + friend + popular + bullied + adults

hyper =~ restless + fidgety + distractible + reflective + persistent

behav =~ tempers + obedient + fights + lies + steals

emo =~ somatic + worries + unhappy + clingy + fears'

threeSDQ <- 'pro =~ considerate + shares + caring + kind + helps

int =~ solitary + friend + popular + bullied + adults +

somatic + worries + unhappy + clingy + fears

ext =~ restless + fidgety + distractible + reflective + persistent +

tempers + obedient + fights + lies + steals'

propSDQ <- 'pro =~ considerate + shares + caring + kind + helps + obedient + friend + popular

+ reflective + persistent

int =~ solitary + bullied + adults + tempers + fights + steals + lies

ext =~ unhappy + clingy + fears + worries + somatic

hyp =~ restless + fidgety + distractible '

ordervar <- c("considerate","shares","caring","kind","helps","solitary","friend",

"popular", "bullied", "adults", "restless", "fidgety",

"distractible","reflective","persistent","tempers","obedient","fights",

"steals","lies","somatic","worries","unhappy","clingy","fears")

tradSDQstruc <- as.vector(c(rep(1,5), rep(2,5), rep(3,5), rep(4,5), rep(5,5)))

names(tradSDQstruc) <- c("considerate", "shares", "caring", "kind", "helps",

"restless", "fidgety", "distractible", "reflective", "persistent",

"somatic", "worries", "unhappy", "clingy", "fears",

"tempers", "obedient", "fights", "lies", "steals",

"solitary", "friend", "popular", "bullied", "adults")

threeSDQstruc <- as.vector(c(rep(1,5), rep(2,5), rep(3,5), rep(2,5), rep(3,5)))

names(threeSDQstruc) <- c("considerate", "shares", "caring", "kind", "helps",

"restless", "fidgety", "distractible", "reflective", "persistent",

"somatic", "worries", "unhappy", "clingy", "fears",

"tempers", "obedient", "fights", "lies", "steals",

"solitary", "friend", "popular", "bullied", "adults")

propSDQstruc <- as.vector(c(rep(1,5), rep(2,3), rep(1,2), rep(3,5), rep(4,1),

rep(1,1), rep(4,3), rep(4,1), rep(1,2), rep(4,2)))

names(propSDQstruc) <- c("considerate", "shares", "caring", "kind", "helps",

"restless", "fidgety", "distractible", "reflective", "persistent",

"somatic", "worries", "unhappy", "clingy", "fears",

"tempers", "obedient", "fights", "lies", "steals",

"solitary", "friend", "popular", "bullied", "adults")

a <- c(1,0,0,0,0)

b <- c(0,1,0,0,0)

c <- c(0,0,1,0,0)

d <- c(0,0,0,1,0)

e <- c(0,0,0,0,1)

n <- 5

sdqfacstruc <- rbind(

as.data.frame(do.call("rbind", replicate(n, a, simplify = FALSE))),

as.data.frame(do.call("rbind", replicate(n, b, simplify = FALSE))),

as.data.frame(do.call("rbind", replicate(n, c, simplify = FALSE))),

as.data.frame(do.call("rbind", replicate(n, d, simplify = FALSE))),

as.data.frame(do.call("rbind", replicate(n, e, simplify = FALSE))))

sdqfacstruc <- as.matrix(sdqfacstruc)

# Fit of the factor model 5-factor structure #

tradSDQnewdataset <- cfa(tradSDQ, sample.cov =CorFin2smooth,

sample.nobs =nrow(newdataset),

estimator = "ML",

std.lv=TRUE)

round(c(fitmeasures(tradSDQnewdataset)[3:7],

fitmeasures(tradSDQnewdataset)[9],

fitmeasures(tradSDQnewdataset)[23:25],

fitmeasures(tradSDQnewdataset)[29]), digits=3)

EGAnet::tefi(CorFin2smooth,tradSDQstruc) #Lower values suggest better fit

# Fit of the factor model 3-factor structure #

threeSDQnewdataset <- cfa(threeSDQ, sample.cov =CorFin2smooth,

sample.nobs =nrow(newdataset[,1:25]),

estimator = "ML",

std.lv=TRUE)

round(c(fitmeasures(threeSDQnewdataset)[3:7],

fitmeasures(threeSDQnewdataset)[9],

fitmeasures(threeSDQnewdataset)[23:25],

fitmeasures(threeSDQnewdataset)[29]), digits=3)

EGAnet::tefi(CorFin2smooth,threeSDQstruc) #Lower values suggest better fit

# Fit of the proposed 4-factor structure #

propSDQnewdataset <- cfa(propSDQ, sample.cov =CorFin2smooth,

sample.nobs =nrow(newdataset[,1:25]),

estimator = "ML",

std.lv=TRUE)

round(c(fitmeasures(propSDQnewdataset)[3:7],

fitmeasures(propSDQnewdataset)[9],

fitmeasures(propSDQnewdataset)[23:25],

fitmeasures(propSDQnewdataset)[29]), digits=3)

EGAnet::tefi(CorFin2smooth,propSDQstruc) #Lower values suggest better fit

# Fit of the network model #

adj <- 1*(network1!=0)

nwModelcross <- ggm(covs = CorFin2smooth, corinput=TRUE,

omega = adj,

nobs = nrow(newdataset[,1:25]))

results_nwModelcross <- nwModelcross %>% runmodel

fitnetwork3K <- fit(results_nwModelcross)

fitnetwork3K[c(7,9:11,21:24),c(1:2)] %>%

mutate_if(is.numeric, round, digits=3)

EGAnet::tefi(CorFin2smooth,ndim$wc) #Lower values suggest better fit

#########################

### UNCONSTRAINED CFA ###

#########################

#CFA (unrestricted) 3-factor structure

threeSDQunres_efa <- fa(newdataset[,1:25], cor="poly", nfactors=3, rotate = "geominQ", fm = 'ml', delta = .5)

threeSDQunres_loadings <- data.table(matrix(round(threeSDQunres_efa$loadings, 3),

nrow = 25, ncol = 3))

names(threeSDQunres_loadings) <- c("F1","F2","F3")

threeSDQunres_loadings$item <- paste0(colnames(newdataset[,1:25]))

threeSDQunres_loadings <- melt(threeSDQunres_loadings, "item", variable.name = "latent")

anchors <- NA

for (l in 1:length(unique(threeSDQunres_loadings$latent))) {

anchors[l] <- threeSDQunres_loadings[threeSDQunres_loadings$value==max(threeSDQunres_loadings$value[threeSDQunres_loadings$latent==unique(threeSDQunres_loadings$latent)[l]]),][[1]]

}

#make model

threeSDQunres_model <- make_esem_model(threeSDQunres_loadings, anchors)

#print model

writeLines(threeSDQunres_model)

threeSDQunres_fitSAABC <- cfa(threeSDQunres_model, sample.cov =CorFin2smooth,

sample.nobs =nrow(newdataset[,1:25]), std.lv=T, estimator = "ML")

summary(threeSDQunres_fitSAABC, fit.measures = T, standardized = T)

round(c(fitmeasures(threeSDQunres_fitSAABC)[3:7],

fitmeasures(threeSDQunres_fitSAABC)[9],

fitmeasures(threeSDQunres_fitSAABC)[23:25],

fitmeasures(threeSDQunres_fitSAABC)[29]), digits=3)

#Get simple structure for TEFIvn

structurethreeSDQunres<-NA

for (l in 1:length(colnames(newdataset[,1:25]))){

structurethreeSDQunres[l]=substr(names(which(coef(threeSDQunres_fitSAABC)==

max(coef(threeSDQunres_fitSAABC)[1:(which(names(coef(threeSDQunres_fitSAABC))=="considerate~~considerate")-1)]

[str_detect(names(coef(threeSDQunres_fitSAABC)

[1:(which(names(coef(threeSDQunres_fitSAABC))=="considerate~~considerate")-1)]),

colnames(newdataset[,1:25])[l])==TRUE]))),

start=2, stop=2)

}

structurethreeSDQunres <- as.numeric(structurethreeSDQunres)

names(structurethreeSDQunres) <- colnames(newdataset[,1:25])

unique(structurethreeSDQunres)

TEFIvnSAABCthreeunres <- EGAnet::tefi(CorFin2smooth,structurethreeSDQunres) #Lower values suggest better fit

TEFIvnSAABCthreeunres

#CFA (unrestricted) 5-factor structure

tradSDQunres_efa <- fa(newdataset[,1:25], cor="poly", nfactors=5, rotate = "geominQ", fm = 'ml', delta = .5)

tradSDQunres_loadings <- data.table(matrix(round(tradSDQunres_efa$loadings, 5),

nrow = 25, ncol = 5))

names(tradSDQunres_loadings) <- c("F1","F2","F3", "F4", "F5")

tradSDQunres_loadings$item <- paste0(colnames(newdataset[,1:25]))

tradSDQunres_loadings <- melt(tradSDQunres_loadings, "item", variable.name = "latent")

anchors <- NA

for (l in 1:length(unique(tradSDQunres_loadings$latent))) {

anchors[l] <- tradSDQunres_loadings[tradSDQunres_loadings$value==max(tradSDQunres_loadings$value[tradSDQunres_loadings$latent==unique(tradSDQunres_loadings$latent)[l]]),][[1]]

}

names(anchors) <- c("F1","F2","F3", "F4", "F5")

#make model

tradSDQunres_model <- make_esem_model(tradSDQunres_loadings, anchors)

#print model

writeLines(tradSDQunres_model)

tradSDQunres_fitSAABC <- cfa(tradSDQunres_model, sample.cov =CorFin2smooth,

sample.nobs =nrow(newdataset[,1:25]), std.lv=T, estimator = "ML")

summary(threeSDQunres_fitSAABC, fit.measures = T, standardized = T)

round(c(fitmeasures(tradSDQunres_fitSAABC)[3:7],

fitmeasures(tradSDQunres_fitSAABC)[9],

fitmeasures(tradSDQunres_fitSAABC)[23:25],

fitmeasures(tradSDQunres_fitSAABC)[29]), digits=3)

#Get simple structure for TEFIvn

structuretradSDQunres<-NA

for (l in 1:length(colnames(newdataset[,1:25]))){

structuretradSDQunres[l]=substr(names(which(coef(tradSDQunres_fitSAABC)==

max(coef(tradSDQunres_fitSAABC)[1:(which(names(coef(tradSDQunres_fitSAABC))=="considerate|t1")-1)]

[str_detect(names(coef(tradSDQunres_fitSAABC)

[1:(which(names(coef(tradSDQunres_fitSAABC))=="considerate|t1")-1)]),

colnames(newdataset[,1:25])[l])==TRUE]))),

start=2, stop=2)

}

structuretradSDQunres <- as.numeric(structuretradSDQunres)

names(structuretradSDQunres) <- colnames(newdataset[,1:25])

unique(structuretradSDQunres)

TEFIvnSAABCtradunres <- EGAnet::tefi(newdataset[,1:25],structuretradSDQunres) #Lower values suggest better fit

TEFIvnSAABCtradunres

###############################

###############################

### LSIC - WAVE 3K ###

### CHILDREN AGED 4 to 10 ###

###############################

###############################

### Opening the dataset ###

dfwave3 <- read_sav("lsiccombinedw3_100c.sav")

nrow(dfwave3)

dfwave3 <- as.data.frame(dfwave3)

dfwave3K <- dfwave3[dfwave3$xcohort==2,]

dfwave3K <- as.data.frame(dfwave3K)

dfwave3K$age <- ifelse(dfwave3K$cascagem==-9,dfwave3K$cascagem,floor(dfwave3K$cascagem/12))

c(mean(dfwave3K$age),sd(dfwave3K$age),min(subset(dfwave3K$age,dfwave3K$age!=-9)),max(dfwave3K$age))

(length(which(dfwave3K$age==-9))/nrow(dfwave3K))*100

newdataLSICwave3K <- cbind(dfwave3K$casq2_1, dfwave3K$casq2_4, dfwave3K$casq2_9, dfwave3K$casq2_17, dfwave3K$casq2_20,

dfwave3K$casq2_6, dfwave3K$casq2_11, dfwave3K$casq2_14, dfwave3K$casq2_19, dfwave3K$casq2_23,

dfwave3K$casq2_2, dfwave3K$casq2_10, dfwave3K$casq2_15, dfwave3K$casq2_21, dfwave3K$casq2_25,

dfwave3K$casq2_5, dfwave3K$casq2_7, dfwave3K$casq2_12, dfwave3K$casq2_18, dfwave3K$casq2_22,

dfwave3K$casq2_3, dfwave3K$casq2_8, dfwave3K$casq2_13, dfwave3K$casq2_16, dfwave3K$casq2_24)

newdataLSICwave3K <- as.data.frame(newdataLSICwave3K)

names(newdataLSICwave3K) <- c("Item_1", "Item_4", "Item_9", "Item_17", "Item_20",

"Item_6", "Item_11", "Item_14", "Item_19", "Item_23",

"Item_2", "Item_10", "Item_15", "Item_21", "Item_25",

"Item_5", "Item_7", "Item_12", "Item_18", "Item_22",

"Item_3", "Item_8", "Item_13", "Item_16", "Item_24")

newdataLSICwave3K[newdataLSICwave3K == -9]<- NA

newdataLSICwave3K[newdataLSICwave3K == -6]<- NA

newdataLSICwave3K[newdataLSICwave3K == -3]<- NA

newdataLSICwave3K[newdataLSICwave3K == -2]<- NA

newdataLSICwave3K <- reverse.code(keysSDQ, newdataLSICwave3K)

newdatasetLSICwave3Kcc <- newdataLSICwave3K[complete.cases(newdataLSICwave3K),]

nrow(newdatasetLSICwave3Kcc)

newdatasetLSICwave3K <- newdatasetLSICwave3Kcc

newdatasetLSICwave3K <- as.data.frame(newdatasetLSICwave3K)

names(newdatasetLSICwave3K) <- c("considerate", "shares", "caring", "kind", "helps",

"solitary", "friend", "popular", "bullied", "adults",

"restless", "fidgety", "distractible", "reflective", "persistent",

"tempers", "obedient", "fights", "lies", "steals",

"somatic", "worries", "unhappy", "clingy", "fears")

##############################

### Preparing for analysis ###

##############################

CorMatLSICwave3K <- qgraph::cor_auto(newdatasetLSICwave3K[,1:25], npn.SKEPTIC =FALSE)

fivescalesLSICwave3K <- list("Prosocial" = c(1:5), "Hyper"=c(6:10), "Emotion" = c(11:15),

"Conduct"=c(16:20), "Peer"=c(21:25))

CorFinLSICwave3K <- CorMatLSICwave3K

########################

### Network Analysis ###

########################

#Network estimation and plot#

network1LSICwave3K <- EBICglasso(CorFinLSICwave3K, n=nrow(newdatasetLSICwave3K))

png("networkLSICwave3Kimp.png", res=300, height=5000, width=5000)

networkplotLSICwave3K <- qgraph(network1LSICwave3K, layout="spring", cut=0,

theme="colorblind", vsize=7.5, esize=18,

groups=fivescales, legend=FALSE,

labels=colnames(newdataset[1:25]))

dev.off()

#Dimensionality#

ndimLSICwave3K <- EGAnet::EGA(CorFinLSICwave3K, n=nrow(newdatasetLSICwave3K))

bootdimenLSICwave3K <- EGAnet::bootEGA(newdatasetLSICwave3K[,1:25], model="glasso", n=2500, ncores=2)

bootdimenLSICwave3K$frequency

png("networkLSICwave3KEGA.png", res=300, height=5000, width=5000)

ndimplotLSICwave3K <- plot(ndimLSICwave3K, theme="colorblind", vsize=7.5, esize=18, layout="spring", legend=FALSE,

cut=0, labels=colnames(newdataset[1:25]))

dev.off()

dimstabLSICwave3K <- dimStability(bootdimenLSICwave3K, orig.wc =ndimLSICwave3K$wc)

###########################

### REDUNDANCY ANALYSIS ###

###########################

# Round 1 #

redLSICWave3K <- EGAnet::UVA(newdatasetLSICwave3K[,1:25], method="wTO", type="adapt", reduce=FALSE,

reduce.method="latent", adhoc=FALSE)

redLSICWave3K$redundancy$descriptives$centralTendency

redLSICWave3K <- EGAnet::UVA(newdatasetLSICwave3K[,1:25], method="wTO", type="adapt", reduce=TRUE,

reduce.method="latent", adhoc=TRUE)

egaredLSICWave3K <- EGA(redLSICWave3K$reduced$data, algorithm = "walktrap")

bootdimenLSACegaredLSICWave3K <- EGAnet::bootEGA(redLSICWave3K$reduced$data, model="glasso", n=2500, ncores=2) #bootstraps the number of dimensions

bootdimenLSACegaredLSICWave3K$summary.table #Summary table

bootdimenLSACegaredLSICWave3K$frequency #Frequency dimensions

###################################

### DETERMINE FACTOR OR NETWORK ###

###################################

# Fit of the factor model 5-factor structure #

tradSDQnewdatasetLSICwave3K <- cfa(tradSDQ, sample.cov =CorFinLSICwave3K,

sample.nobs =nrow(newdatasetLSICwave3K[,1:25]),

estimator = "ML",

std.lv=TRUE)

round(c(fitmeasures(tradSDQnewdatasetLSICwave3K)[3:7],

fitmeasures(tradSDQnewdatasetLSICwave3K)[9],

fitmeasures(tradSDQnewdatasetLSICwave3K)[23:25],

fitmeasures(tradSDQnewdatasetLSICwave3K)[29]), digits=3)

EGAnet::tefi(CorFinLSICwave3K,tradSDQstruc) #Lower values suggest better fit

# Fit of the factor model 3-factor structure #

threeSDQnewdatasetLSICwave3K <- cfa(threeSDQ, sample.cov =CorFinLSICwave3K,

sample.nobs =nrow(newdatasetLSICwave3K[,1:25]),

estimator = "ML",

std.lv=TRUE)

round(c(fitmeasures(threeSDQnewdatasetLSICwave3K)[3:7],

fitmeasures(threeSDQnewdatasetLSICwave3K)[9],

fitmeasures(threeSDQnewdatasetLSICwave3K)[23:25],

fitmeasures(threeSDQnewdatasetLSICwave3K)[29]), digits=3)

EGAnet::tefi(CorFinLSICwave3K,threeSDQstruc) #Lower values suggest better fit

# Fit of the proposed 4-factor structure #

propSDQnewdatasetLSICwave3K <- cfa(propSDQ, sample.cov =CorFinLSICwave3K,

sample.nobs =nrow(newdatasetLSICwave3K[,1:25]),

estimator = "ML",

std.lv=TRUE)

round(c(fitmeasures(propSDQnewdatasetLSICwave3K)[3:7],

fitmeasures(propSDQnewdatasetLSICwave3K)[9],

fitmeasures(propSDQnewdatasetLSICwave3K)[23:25],

fitmeasures(propSDQnewdatasetLSICwave3K)[29]), digits=3)

EGAnet::tefi(CorFinLSICwave3K,propSDQstruc) #Lower values suggest better fit

# Fit of the network model #

adj <- 1*(network1LSICwave3K!=0)

nwModelcross <- ggm(covs = CorFinLSICwave3K, corinput=TRUE,

omega = adj,

nobs = nrow(newdatasetLSICwave3K[,1:25]))

results_nwModelcross <- nwModelcross %>% runmodel

fitnetwork3K <- fit(results_nwModelcross)

fitnetwork3K[c(7,9:11,21:24),c(1:2)] %>%

mutate_if(is.numeric, round, digits=3)

EGAnet::tefi(CorFinLSICwave3K,ndimLSICwave3K$wc) #Lower values suggest better fit

#########################

### UNCONSTRAINED CFA ###

#########################

#CFA (unrestricted) 3-factor structure

threeSDQunres_efa <- fa(newdatasetLSICwave3K[,1:25], cor="poly", nfactors=3, rotate = "geominQ", fm = 'ml', delta = .5)

threeSDQunres_loadings <- data.table(matrix(round(threeSDQunres_efa$loadings, 3),

nrow = 25, ncol = 3))

names(threeSDQunres_loadings) <- c("F1","F2","F3")

threeSDQunres_loadings$item <- paste0(colnames(newdatasetLSICwave3K))

threeSDQunres_loadings <- melt(threeSDQunres_loadings, "item", variable.name = "latent")

anchors <- NA

for (l in 1:length(unique(threeSDQunres_loadings$latent))) {

anchors[l] <- threeSDQunres_loadings[threeSDQunres_loadings$value==max(threeSDQunres_loadings$value[threeSDQunres_loadings$latent==unique(threeSDQunres_loadings$latent)[l]]),][[1]]

}

#make model

threeSDQunres_model <- make_esem_model(threeSDQunres_loadings, anchors)

#print model

writeLines(threeSDQunres_model)

threeSDQunres_fit3K <- cfa(threeSDQunres_model, sample.cov =CorFinLSICwave3K,

sample.nobs =nrow(newdatasetLSICwave3K[,1:25]), std.lv=T, estimator = "ML")

summary(threeSDQunres_fit3K, fit.measures = T, standardized = T)

round(c(fitmeasures(threeSDQunres_fit3K)[3:7],

fitmeasures(threeSDQunres_fit3K)[9],

fitmeasures(threeSDQunres_fit3K)[23:25],

fitmeasures(threeSDQunres_fit3K)[29]), digits=3)

#Get simple structure for TEFIvn

structurethreeSDQunres<-NA

for (l in 1:length(colnames(newdatasetLSICwave3K[,1:25]))){

structurethreeSDQunres[l]=substr(names(which(coef(threeSDQunres_fit3K)==

max(coef(threeSDQunres_fit3K)[1:(which(names(coef(threeSDQunres_fit3K))=="considerate~~considerate")-1)]

[str_detect(names(coef(threeSDQunres_fit3K)

[1:(which(names(coef(threeSDQunres_fit3K))=="considerate~~considerate")-1)]),

colnames(newdatasetLSICwave3K[,1:25])[l])==TRUE]))),

start=2, stop=2)

}

structurethreeSDQunres <- as.numeric(structurethreeSDQunres)

names(structurethreeSDQunres) <- colnames(newdatasetLSICwave3K[,1:25])

unique(structurethreeSDQunres)

TEFIvn3kthreeunres <- EGAnet::tefi(newdatasetLSICwave3K[,1:25],structurethreeSDQunres) #Lower values suggest better fit

TEFIvn3kthreeunres

#CFA (unrestricted) 5-factor structure

tradSDQunres_efa <- fa(newdatasetLSICwave3K, cor="poly", nfactors=5, rotate = "geominQ", fm = 'ml', delta = .5)

tradSDQunres_loadings <- data.table(matrix(round(tradSDQunres_efa$loadings, 5),

nrow = 25, ncol = 5))

names(tradSDQunres_loadings) <- c("F1","F2","F3", "F4", "F5")

tradSDQunres_loadings$item <- paste0(colnames(newdatasetLSICwave3K))

tradSDQunres_loadings <- melt(tradSDQunres_loadings, "item", variable.name = "latent")

anchors <- NA

for (l in 1:length(unique(tradSDQunres_loadings$latent))) {

anchors[l] <- tradSDQunres_loadings[tradSDQunres_loadings$value==max(tradSDQunres_loadings$value[tradSDQunres_loadings$latent==unique(tradSDQunres_loadings$latent)[l]]),][[1]]

}

names(anchors) <- c("F1","F2","F3", "F4", "F5")

#make model

tradSDQunres_model <- make_esem_model(tradSDQunres_loadings, anchors)

#print model

writeLines(tradSDQunres_model)

tradSDQunres_fit3K <- cfa(tradSDQunres_model, sample.cov =CorFinLSICwave3K,

sample.nobs =nrow(newdatasetLSICwave3K[,1:25]), std.lv=T, estimator = "ML")

summary(tradSDQunres_fit3K, fit.measures = T, standardized = T)

round(c(fitmeasures(tradSDQunres_fit3K)[3:7],

fitmeasures(tradSDQunres_fit3K)[9],

fitmeasures(tradSDQunres_fit3K)[23:25],

fitmeasures(tradSDQunres_fit3K)[29]), digits=3)

#Get simple structure for TEFIvn

structuretradSDQunres<-NA

for (l in 1:length(colnames(newdatasetLSICwave3K[,1:25]))){

structuretradSDQunres[l]=substr(names(which(coef(tradSDQunres_fit3K)==

max(coef(tradSDQunres_fit3K)[1:(which(names(coef(tradSDQunres_fit3K))=="considerate~~considerate")-1)]

[str_detect(names(coef(tradSDQunres_fit3K)

[1:(which(names(coef(tradSDQunres_fit3K))=="considerate~~considerate")-1)]),

colnames(newdatasetLSICwave3K[,1:25])[l])==TRUE]))),

start=2, stop=2)

}

structuretradSDQunres <- as.numeric(structuretradSDQunres)

names(structuretradSDQunres) <- colnames(newdatasetLSICwave3K[,1:25])

unique(structuretradSDQunres)

TEFIvn3ktradunres <- EGAnet::tefi(newdatasetLSICwave3K[,1:25],structuretradSDQunres) #Lower values suggest better fit

TEFIvn3ktradunres

###############################

###############################

### LSIC - WAVE 4K ###

### CHILDREN AGED 4 to 10 ###

###############################

###############################

dfwave4 <- read_sav("lsiccombinedw4_100c.sav")

nrow(dfwave4)

dfwave4K <- dfwave4[dfwave4$xcohort==2,]

dfwave4K <- as.data.frame(dfwave4K)

dfwave4K$age <- ifelse(dfwave4K$dascagem==-9,dfwave4K$dascagem,floor(dfwave4K$dascagem/12))

c(mean(dfwave4K$age),sd(dfwave4K$age),min(subset(dfwave4K$age,dfwave4K$age!=-9)),max(dfwave4K$age))

(length(which(dfwave4K$age==-9))/nrow(dfwave4K))*100

newdataLSICwave4K <- cbind(dfwave4K$dasq2_1, dfwave4K$dasq2_4, dfwave4K$dasq2_9, dfwave4K$dasq2_17, dfwave4K$dasq2_20,

dfwave4K$dasq2_6, dfwave4K$dasq2_11, dfwave4K$dasq2_14, dfwave4K$dasq2_19, dfwave4K$dasq2_23,

dfwave4K$dasq2_2, dfwave4K$dasq2_10, dfwave4K$dasq2_15, dfwave4K$dasq2_21, dfwave4K$dasq2_25,

dfwave4K$dasq2_5, dfwave4K$dasq2_7, dfwave4K$dasq2_12, dfwave4K$dasq2_18, dfwave4K$dasq2_22,

dfwave4K$dasq2_3, dfwave4K$dasq2_8, dfwave4K$dasq2_13, dfwave4K$dasq2_16, dfwave4K$dasq2_24)

newdataLSICwave4K <- as.data.frame(newdataLSICwave4K)

names(newdataLSICwave4K) <- c("Item_1", "Item_4", "Item_9", "Item_17", "Item_20",

"Item_6", "Item_11", "Item_14", "Item_19", "Item_23",

"Item_2", "Item_10", "Item_15", "Item_21", "Item_25",

"Item_5", "Item_7", "Item_12", "Item_18", "Item_22",

"Item_3", "Item_8", "Item_13", "Item_16", "Item_24")

newdataLSICwave4K[newdataLSICwave4K == -9]<- NA

newdataLSICwave4K[newdataLSICwave4K == -6]<- NA

newdataLSICwave4K[newdataLSICwave4K == -3]<- NA

newdataLSICwave4K[newdataLSICwave4K == -2]<- NA

newdataLSICwave4K <- reverse.code(keysSDQ, newdataLSICwave4K)

newdataLSICwave4K <- as.data.frame(newdataLSICwave4K)

newdatasetLSICwave4Kcc <- newdataLSICwave4K[complete.cases(newdataLSICwave4K),]

newdatasetLSICwave4K <- newdatasetLSICwave4Kcc

names(newdatasetLSICwave4K) <- c("considerate", "shares", "caring", "kind", "helps",

"solitary", "friend", "popular", "bullied", "adults",

"restless", "fidgety", "distractible", "reflective", "persistent",

"tempers", "obedient", "fights", "lies", "steals",

"somatic", "worries", "unhappy", "clingy", "fears")

##############################

### Preparing for analysis ###

##############################

CorMatLSICwave4K <- qgraph::cor_auto(newdatasetLSICwave4K[,1:25], npn.SKEPTIC=FALSE)

fivescalesLSICwave4K <- list("Prosocial" = c(1:5), "Hyper"=c(6:10), "Emotion" = c(11:15),

"Conduct"=c(16:20), "Peer"=c(21:25))

CorFinLSICwave4K <- CorMatLSICwave4K

########################

### Network Analysis ###

########################

#Network estimation and plot#

network1LSICwave4K <- EBICglasso(CorFinLSICwave4K, n=nrow(newdatasetLSICwave4K))

png("networkLSICwave4K.png", res=300, height=5000, width=5000)

networkplotLSICwave4K <- qgraph(network1LSICwave4K, layout="spring", cut=0,

theme="colorblind", vsize=7.5, esize=18,

legend=FALSE, groups=fivescales,

labels=colnames(newdataset[1:25]))

dev.off()

#Dimensionality#

ndimLSICwave4K <- EGAnet::EGA(CorFinLSICwave4K, n=nrow(newdatasetLSICwave4K))

bootdimenLSICwave4K <- EGAnet::bootEGA(newdatasetLSICwave4K[,1:25], model="glasso", n=2500, ncores=2)

bootdimenLSICwave4K$summary.table

bootdimenLSICwave4K$frequency

png("networkLSICwave4KEGA.png", res=300, height=5000, width=5000)

ndimplotLSICwave4K <- plot(ndimLSICwave4K, theme="colorblind", vsize=7.5, esize=18, layout="spring", legend=FALSE,

cut=0, labels=colnames(newdataset[1:25]))

dev.off()

dimstabLSICwave4K <- dimStability(bootdimenLSICwave4K, orig.wc =ndimLSICwave4K$wc)

###########################

### REDUNDANCY ANALYSIS ###

###########################

# Round 1 #

redLSICWave4K <- EGAnet::UVA(newdatasetLSICwave4K[,1:25], method="wTO", type="adapt", reduce=FALSE,

reduce.method="latent", adhoc=FALSE)

head(redLSICWave4K$redundancy$descriptives$centralTendency)

redLSICWave4K <- EGAnet::UVA(newdatasetLSICwave4K[,1:25], method="wTO", type="adapt", reduce=TRUE,

reduce.method="latent", adhoc=TRUE)

egaredLSICWave4K <- EGA(redLSICWave4K$reduced$data, algorithm = "walktrap")

bootdimenLSACegaredLSICWave4K <- EGAnet::bootEGA(redLSICWave4K$reduced$data, model="glasso", n=2500, ncores=2) #bootstraps the number of dimensions

bootdimenLSACegaredLSICWave4K$summary.table #Summary table

bootdimenLSACegaredLSICWave4K$frequency #Frequency dimensions

###################################

### DETERMINE FACTOR OR NETWORK ###

###################################

# Fit of the factor model 5-factor structure #

tradSDQnewdatasetLSICwave4K <- cfa(tradSDQ, sample.cov =CorFinLSICwave4K,

sample.nobs =nrow(newdatasetLSICwave4K[,1:25]),

estimator = "ML",

std.lv=TRUE)

round(c(fitmeasures(tradSDQnewdatasetLSICwave4K)[3:7],

fitmeasures(tradSDQnewdatasetLSICwave4K)[9],

fitmeasures(tradSDQnewdatasetLSICwave4K)[23:25],

fitmeasures(tradSDQnewdatasetLSICwave4K)[29]), digits=3)

EGAnet::tefi(CorFinLSICwave4K,tradSDQstruc) #Lower values suggest better fit

# Fit of the factor model 3-factor structure #

threeSDQnewdatasetLSICwave4K <- cfa(threeSDQ, sample.cov =CorFinLSICwave4K,

sample.nobs =nrow(newdatasetLSICwave4K[,1:25]),

estimator = "ML",

std.lv=TRUE)

round(c(fitmeasures(threeSDQnewdatasetLSICwave4K)[3:7],

fitmeasures(threeSDQnewdatasetLSICwave4K)[9],

fitmeasures(threeSDQnewdatasetLSICwave4K)[23:25],

fitmeasures(threeSDQnewdatasetLSICwave4K)[29]), digits=3)

EGAnet::tefi(CorFinLSICwave4K,threeSDQstruc) #Lower values suggest better fit

# Fit of the proposed 4-factor structure #

propSDQnewdatasetLSICwave4K <- cfa(propSDQ, sample.cov =CorFinLSICwave4K,

sample.nobs =nrow(newdatasetLSICwave4K[,1:25]),

estimator = "ML",

std.lv=TRUE)

round(c(fitmeasures(propSDQnewdatasetLSICwave4K)[3:7],

fitmeasures(propSDQnewdatasetLSICwave4K)[9],

fitmeasures(propSDQnewdatasetLSICwave4K)[23:25],

fitmeasures(propSDQnewdatasetLSICwave4K)[29]), digits=3)

EGAnet::tefi(CorFinLSICwave4K,propSDQstruc) #Lower values suggest better fit

# Fit of the network model #

adj <- 1*(network1LSICwave4K!=0)

nwModelcross <- ggm(covs = CorFinLSICwave4K, corinput=TRUE,

omega = adj,

nobs = nrow(newdatasetLSICwave4K[,1:25]))

results_nwModelcross <- nwModelcross %>% runmodel

fitnetwork4K <- fit(results_nwModelcross)

fitnetwork4K[c(7,9:11,21:24),c(1:2)] %>%

mutate_if(is.numeric, round, digits=3)

EGAnet::tefi(CorFinLSICwave4K,ndimLSICwave4K$wc) #Lower values suggest better fit

#########################

### UNCONSTRAINED CFA ###

#########################

#CFA (unrestricted) 3-factor structure

threeSDQunres_efa <- fa(newdatasetLSICwave4K[,1:25], cor="poly", nfactors=3, rotate = "geominQ", fm = 'ml', delta = .5)

threeSDQunres_loadings <- data.table(matrix(round(threeSDQunres_efa$loadings, 3),

nrow = 25, ncol = 3))

names(threeSDQunres_loadings) <- c("F1","F2","F3")

threeSDQunres_loadings$item <- paste0(colnames(newdatasetLSICwave4K))

threeSDQunres_loadings <- melt(threeSDQunres_loadings, "item", variable.name = "latent")

anchors <- NA

for (l in 1:length(unique(threeSDQunres_loadings$latent))) {

anchors[l] <- threeSDQunres_loadings[threeSDQunres_loadings$value==max(threeSDQunres_loadings$value[threeSDQunres_loadings$latent==unique(threeSDQunres_loadings$latent)[l]]),][[1]]

}

#make model

threeSDQunres_model <- make_esem_model(threeSDQunres_loadings, anchors)

#print model

writeLines(threeSDQunres_model)

threeSDQunres_fit4K <- cfa(threeSDQunres_model, sample.cov =CorFinLSICwave4K,

sample.nobs =nrow(newdatasetLSICwave4K[,1:25]), std.lv=T, estimator = "ML")

summary(threeSDQunres_fit4K, fit.measures = T, standardized = T)

round(c(fitmeasures(threeSDQunres_fit4K)[3:7],

fitmeasures(threeSDQunres_fit4K)[9],

fitmeasures(threeSDQunres_fit4K)[23:25],

fitmeasures(threeSDQunres_fit4K)[29]), digits=3)

#Get simple structure for TEFIvn

structurethreeSDQunres<-NA

for (l in 1:length(colnames(newdatasetLSICwave4K[,1:25]))){

structurethreeSDQunres[l]=substr(names(which(coef(threeSDQunres_fit4K)==

max(coef(threeSDQunres_fit4K)[1:(which(names(coef(threeSDQunres_fit4K))=="considerate~~considerate")-1)]

[str_detect(names(coef(threeSDQunres_fit4K)

[1:(which(names(coef(threeSDQunres_fit4K))=="considerate~~considerate")-1)]),

colnames(newdatasetLSICwave4K[,1:25])[l])==TRUE]))),

start=2, stop=2)

}

structurethreeSDQunres <- as.numeric(structurethreeSDQunres)

names(structurethreeSDQunres) <- colnames(newdatasetLSICwave4K[,1:25])

unique(structurethreeSDQunres)

TEFIvn4kthreeunres <- EGAnet::tefi(newdatasetLSICwave4K[,1:25],structurethreeSDQunres) #Lower values suggest better fit

TEFIvn4kthreeunres

#CFA (unrestricted) 5-factor structure

tradSDQunres_efa <- fa(newdatasetLSICwave4K, cor="poly", nfactors=5, rotate = "geominQ", fm = 'ml', delta = 0.5)

tradSDQunres_loadings <- data.table(matrix(round(tradSDQunres_efa$loadings, 5),

nrow = 25, ncol = 5))

names(tradSDQunres_loadings) <- c("F1","F2","F3", "F4", "F5")

tradSDQunres_loadings$item <- paste0(colnames(newdatasetLSICwave4K))

tradSDQunres_loadings <- melt(tradSDQunres_loadings, "item", variable.name = "latent")

anchors <- NA

for (l in 1:length(unique(tradSDQunres_loadings$latent))) {

anchors[l] <- tradSDQunres_loadings[tradSDQunres_loadings$value==max(tradSDQunres_loadings$value[tradSDQunres_loadings$latent==unique(tradSDQunres_loadings$latent)[l]]),][[1]]

}

names(anchors) <- c("F1","F2","F3", "F4", "F5")

#make model

tradSDQunres_model <- make_esem_model(tradSDQunres_loadings, anchors)

#print model

writeLines(tradSDQunres_model)

tradSDQunres_fit4K <- cfa(tradSDQunres_model, sample.cov = CorFinLSICwave4K,

sample.nobs =nrow(newdatasetLSICwave4K), std.lv=T, estimator = "ML")

#Get simple structure for TEFIvn

structuretradSDQunres<-NA

for (l in 1:length(colnames(newdatasetLSICwave4K[,1:25]))){

structuretradSDQunres[l]=substr(names(which(coef(tradSDQunres_fit4K)==

max(coef(tradSDQunres_fit4K)[1:(which(names(coef(tradSDQunres_fit4K))=="considerate|t1")-1)]

[str_detect(names(coef(tradSDQunres_fit4K)

[1:(which(names(coef(tradSDQunres_fit4K))=="considerate|t1")-1)]),

colnames(newdatasetLSICwave4K[,1:25])[l])==TRUE]))),

start=2, stop=2)

}

structuretradSDQunres <- as.numeric(structuretradSDQunres)

names(structuretradSDQunres) <- colnames(newdatasetLSICwave4K[,1:25])

unique(structuretradSDQunres)

TEFIvn4ktradunres <- EGAnet::tefi(newdatasetLSICwave4K[,1:25],structuretradSDQunres) #Lower values suggest better fit

TEFIvn4ktradunres

###############################

### LSIC - WAVE 6 ###

### CHILDREN AGED 4 to 10 ###

###############################

###############################

dfwave6 <- read_sav("lsiccombinedw6_100c.sav")

dfwave6 <- as.data.frame(dfwave6)

dfwave6$age <- ifelse(dfwave6$fascagem==-9,dfwave6$fascagem,floor(dfwave6$fascagem/12))

c(mean(dfwave6$age),sd(dfwave6$age),min(subset(dfwave6$age,dfwave6$age!=-9)),max(dfwave6$age))

(length(which(dfwave6$age==-9))/nrow(dfwave6))*100

newdataLSICwave6 <- cbind(dfwave6$fasq2_1, dfwave6$fasq2_4, dfwave6$fasq2_9, dfwave6$fasq2_17, dfwave6$fasq2_20,

dfwave6$fasq2_6, dfwave6$fasq2_11, dfwave6$fasq2_14, dfwave6$fasq2_19, dfwave6$fasq2_23,

dfwave6$fasq2_2, dfwave6$fasq2_10, dfwave6$fasq2_15, dfwave6$fasq2_21, dfwave6$fasq2_25,

dfwave6$fasq2_5, dfwave6$fasq2_7, dfwave6$fasq2_12, dfwave6$fasq2_18, dfwave6$fasq2_22,

dfwave6$fasq2_3, dfwave6$fasq2_8, dfwave6$fasq2_13, dfwave6$fasq2_16, dfwave6$fasq2_24)

newdataLSICwave6 <- as.data.frame(newdataLSICwave6)

names(newdataLSICwave6) <- c("Item_1", "Item_4", "Item_9", "Item_17", "Item_20",

"Item_6", "Item_11", "Item_14", "Item_19", "Item_23",

"Item_2", "Item_10", "Item_15", "Item_21", "Item_25",

"Item_5", "Item_7", "Item_12", "Item_18", "Item_22",

"Item_3", "Item_8", "Item_13", "Item_16", "Item_24")

newdataLSICwave6[newdataLSICwave6 == -9]<- NA

newdataLSICwave6[newdataLSICwave6 == -6]<- NA

newdataLSICwave6[newdataLSICwave6 == -3]<- NA

newdataLSICwave6[newdataLSICwave6 == -2]<- NA

newdataLSICwave6 <- reverse.code(keysSDQ, newdataLSICwave6)

newdataLSICwave6 <- as.data.frame(newdataLSICwave6)

newdatasetLSICwave6cc <- newdataLSICwave6[complete.cases(newdataLSICwave6),]

newdatasetLSICwave6 <- newdatasetLSICwave6cc

names(newdatasetLSICwave6) <- c("considerate", "shares", "caring", "kind", "helps",

"solitary", "friend", "popular", "bullied", "adults",

"restless", "fidgety", "distractible", "reflective", "persistent",

"tempers", "obedient", "fights", "lies", "steals",

"somatic", "worries", "unhappy", "clingy", "fears")

##############################

### Preparing for analysis ###

##############################

CorMatLSICwave6 <- qgraph::cor_auto(newdatasetLSICwave6[,1:25], npn.SKEPTIC=FALSE)

fivescalesLSICwave6 <- list("Prosocial" = c(1:5), "Hyper"=c(6:10), "Emotion" = c(11:15),

"Conduct"=c(16:20), "Peer"=c(21:25))

CorFinLSICwave6 <- CorMatLSICwave6

########################

### Network Analysis ###

########################

#Network estimation and plot#

network1LSICwave6 <- EBICglasso(CorFinLSICwave6, n=nrow(newdatasetLSICwave6))

png("networkLSICwave6.png", res=300, height=5000, width=5000)

networkplotLSICwave6 <- qgraph(network1LSICwave6, layout="spring", cut=0,

theme="colorblind", vsize=7.5, esize=18,

legend=FALSE, groups=fivescales,

labels=colnames(newdataset[1:25]))

dev.off()

#Dimensionality#

ndimLSICwave6 <- EGAnet::EGA(CorFinLSICwave6, n=nrow(newdatasetLSICwave6))

bootdimenLSICwave6 <- EGAnet::bootEGA(newdatasetLSICwave6[,1:25], model="glasso", n=2500, ncores=2)

bootdimenLSICwave6$summary.table

bootdimenLSICwave6$frequency

png("networkLSICwave6EGA.png", res=300, height=5000, width=5000)

ndimplotLSICwave6 <- plot(ndimLSICwave6, theme="colorblind", vsize=7.5, esize=18, layout="spring", legend=FALSE,

cut=0, labels=colnames(newdataset[1:25]))

dev.off()

dimstabLSICwave6 <- dimStability(bootdimenLSICwave6, orig.wc =ndimLSICwave6$wc)

###########################

### REDUNDANCY ANALYSIS ###

###########################

# Round 1 #

redLSICWave6 <- EGAnet::UVA(newdatasetLSICwave6[,1:25], method="wTO", type="adapt", reduce=FALSE,

reduce.method="latent", adhoc=FALSE)

head(redLSICWave6$redundancy$descriptives$centralTendency)

redLSICWave6 <- EGAnet::UVA(newdatasetLSICwave6[,1:25], method="wTO", type="adapt", reduce=TRUE,

reduce.method="latent", adhoc=TRUE)

egaredLSICWave6 <- EGA(redLSICWave6$reduced$data, algorithm = "walktrap")

bootdimenLSACegaredLSICWave6 <- EGAnet::bootEGA(redLSICWave6$reduced$data, model="glasso", n=2500, ncores=2) #bootstraps the number of dimensions

bootdimenLSACegaredLSICWave6$summary.table #Summary table

bootdimenLSACegaredLSICWave6$frequency #Frequency dimensions [3 dim - 0.6992]

###################################

### DETERMINE FACTOR OR NETWORK ###

###################################

# Fit of the factor model 5-factor structure #

tradSDQnewdatasetLSICwave6 <- cfa(tradSDQ, sample.cov =CorFinLSICwave6,

sample.nobs =nrow(newdatasetLSICwave6[,1:25]),

estimator = "ML",

std.lv=TRUE)

round(c(fitmeasures(tradSDQnewdatasetLSICwave6)[3:7],

fitmeasures(tradSDQnewdatasetLSICwave6)[9],

fitmeasures(tradSDQnewdatasetLSICwave6)[23:25],

fitmeasures(tradSDQnewdatasetLSICwave6)[29]), digits=3)

EGAnet::tefi(CorFinLSICwave6,tradSDQstruc) #Lower values suggest better fit

# Fit of the factor model 3-factor structure #

threeSDQnewdatasetLSICwave6 <- cfa(threeSDQ, sample.cov =CorFinLSICwave6,

sample.nobs =nrow(newdatasetLSICwave6[,1:25]),

estimator = "ML",

std.lv=TRUE)

round(c(fitmeasures(threeSDQnewdatasetLSICwave6)[3:7],

fitmeasures(threeSDQnewdatasetLSICwave6)[9],

fitmeasures(threeSDQnewdatasetLSICwave6)[23:25],

fitmeasures(threeSDQnewdatasetLSICwave6)[29]), digits=3)

EGAnet::tefi(CorFinLSICwave6,threeSDQstruc) #Lower values suggest better fit

# Fit of the proposed 4-factor structure #

propSDQnewdatasetLSICwave6 <- cfa(propSDQ, sample.cov =CorFinLSICwave6,

sample.nobs =nrow(newdatasetLSICwave6[,1:25]),

estimator = "ML",

std.lv=TRUE)

round(c(fitmeasures(propSDQnewdatasetLSICwave6)[3:7],

fitmeasures(propSDQnewdatasetLSICwave6)[9],

fitmeasures(propSDQnewdatasetLSICwave6)[23:25],

fitmeasures(propSDQnewdatasetLSICwave6)[29]), digits=3)

EGAnet::tefi(CorFinLSICwave6,propSDQstruc) #Lower values suggest better fit

# Fit of the network model #

adj <- 1*(network1LSICwave6!=0)

nwModelcross <- ggm(covs = CorFinLSICwave6, corinput=TRUE,

omega = adj,

nobs = nrow(newdatasetLSICwave6[,1:25]))

results_nwModelcross <- nwModelcross %>% runmodel

fitnetwork6 <- fit(results_nwModelcross)

fitnetwork6[c(7,9:11,21:24),c(1:2)] %>%

mutate_if(is.numeric, round, digits=3)

EGAnet::tefi(CorFinLSICwave6,ndimLSICwave6$wc) #Lower values suggest better fit

#########################

### UNCONSTRAINED CFA ###

#########################

#CFA (unrestricted) 3-factor structure

threeSDQunres_efa <- fa(newdatasetLSICwave6, cor="poly", nfactors=3, rotate = "geominQ", fm = 'ml', delta = .5)

threeSDQunres_loadings <- data.table(matrix(round(threeSDQunres_efa$loadings, 3),

nrow = 25, ncol = 3))

names(threeSDQunres_loadings) <- c("F1","F2","F3")

threeSDQunres_loadings$item <- paste0(colnames(newdatasetLSICwave6))

threeSDQunres_loadings <- melt(threeSDQunres_loadings, "item", variable.name = "latent")

anchors <- NA

for (l in 1:length(unique(threeSDQunres_loadings$latent))) {

anchors[l] <- threeSDQunres_loadings[threeSDQunres_loadings$value==max(threeSDQunres_loadings$value[threeSDQunres_loadings$latent==unique(threeSDQunres_loadings$latent)[l]]),][[1]]

}

#make model

threeSDQunres_model <- make_esem_model(threeSDQunres_loadings, anchors)

#print model

writeLines(threeSDQunres_model)

threeSDQunres_fit6 <- cfa(threeSDQunres_model, sample.cov =CorFinLSICwave6,

sample.nobs =nrow(newdatasetLSICwave6[,1:25]), std.lv=T, estimator = "ML")

summary(threeSDQunres_fit6, fit.measures = T, standardized = T)

round(c(fitmeasures(threeSDQunres_fit6)[3:7],

fitmeasures(threeSDQunres_fit6)[9],

fitmeasures(threeSDQunres_fit6)[23:25],

fitmeasures(threeSDQunres_fit6)[29]), digits=3)

#Get simple structure for TEFIvn

structurethreeSDQunres<-NA

for (l in 1:length(colnames(newdatasetLSICwave6[,1:25]))){

structurethreeSDQunres[l]=substr(names(which(coef(threeSDQunres_fit6)==

max(coef(threeSDQunres_fit6)[1:(which(names(coef(threeSDQunres_fit6))=="considerate~~considerate")-1)]

[str_detect(names(coef(threeSDQunres_fit6)

[1:(which(names(coef(threeSDQunres_fit6))=="considerate~~considerate")-1)]),

colnames(newdatasetLSICwave6[,1:25])[l])==TRUE]))),

start=2, stop=2)

}

structurethreeSDQunres <- as.numeric(structurethreeSDQunres)

names(structurethreeSDQunres) <- colnames(newdatasetLSICwave6[,1:25])

unique(structurethreeSDQunres)

TEFIvn6threeunres <- EGAnet::tefi(newdatasetLSICwave6[,1:25],structurethreeSDQunres) #Lower values suggest better fit

TEFIvn6threeunres

#CFA (unrestricted) 5-factor structure

tradSDQunres_efa <- fa(newdatasetLSICwave6, cor="poly", nfactors=5, rotate = "geominQ", fm = 'ml', delta = .5)

tradSDQunres_loadings <- data.table(matrix(round(tradSDQunres_efa$loadings, 5),

nrow = 25, ncol = 5))

names(tradSDQunres_loadings) <- c("F1","F2","F3", "F4", "F5")

tradSDQunres_loadings$item <- paste0(colnames(newdatasetLSICwave6))

tradSDQunres_loadings <- melt(tradSDQunres_loadings, "item", variable.name = "latent")

anchors <- NA

for (l in 1:length(unique(tradSDQunres_loadings$latent))) {

anchors[l] <- tradSDQunres_loadings[tradSDQunres_loadings$value==max(tradSDQunres_loadings$value[tradSDQunres_loadings$latent==unique(tradSDQunres_loadings$latent)[l]]),][[1]]

}

names(anchors) <- c("F1","F2","F3", "F4", "F5")

#make model

tradSDQunres_model <- make_esem_model(tradSDQunres_loadings, anchors)

#print model

writeLines(tradSDQunres_model)

tradSDQunres_fit6 <- cfa(tradSDQunres_model, sample.cov =CorFinLSICwave6,

sample.nobs =nrow(newdatasetLSICwave6[,1:25]), std.lv=T, estimator = "ML")

summary(tradSDQunres_fit6, fit.measures = T, standardized = T)

round(c(fitmeasures(tradSDQunres_fit6)[3:7],

fitmeasures(tradSDQunres_fit6)[9],

fitmeasures(tradSDQunres_fit6)[23:25],

fitmeasures(tradSDQunres_fit6)[29]), digits=3)

#Get simple structure for TEFIvn

structuretradSDQunres<-NA

for (l in 1:length(colnames(newdatasetLSICwave6[,1:25]))){

structuretradSDQunres[l]=substr(names(which(coef(tradSDQunres_fit6)==

max(coef(tradSDQunres_fit6)[1:(which(names(coef(tradSDQunres_fit6))=="considerate~~considerate")-1)]

[str_detect(names(coef(tradSDQunres_fit6)

[1:(which(names(coef(tradSDQunres_fit6))=="considerate~~considerate")-1)]),

colnames(newdatasetLSICwave6[,1:25])[l])==TRUE]))),

start=2, stop=2)

}

structuretradSDQunres <- as.numeric(structuretradSDQunres)

names(structuretradSDQunres) <- colnames(newdatasetLSICwave6[,1:25])

unique(structuretradSDQunres)

TEFIvn2ktradunres <- EGAnet::tefi(newdatasetLSICwave6[,1:25],structuretradSDQunres) #Lower values suggest better fit

TEFIvn2ktradunres

###############################

### LSIC - WAVE 8 ###

### CHILDREN AGED 4 to 10 ###

###############################

###############################

dfwave8 <- read_sav("lsiccombinedw8_100c.sav")

dfwave8 <- as.data.frame(dfwave8)

dfwave8$age <- ifelse(dfwave8$hascagem==-9,dfwave8$hascagem,floor(dfwave8$hascagem/12))

dfwave8 <- dfwave8[!(dfwave8$age==11 | dfwave8$age==12),]

c(mean(dfwave8$age),sd(dfwave8$age),min(subset(dfwave8$age,dfwave8$age!=-9)),max(dfwave8$age))

(length(which(dfwave8$age==-9))/nrow(dfwave8))*100

newdataLSICwave8 <- cbind(dfwave8$hasq2_1, dfwave8$hasq2_4, dfwave8$hasq2_9, dfwave8$hasq2_17, dfwave8$hasq2_20,

dfwave8$hasq2_6, dfwave8$hasq2_11, dfwave8$hasq2_14, dfwave8$hasq2_19, dfwave8$hasq2_23,

dfwave8$hasq2_2, dfwave8$hasq2_10, dfwave8$hasq2_15, dfwave8$hasq2_21, dfwave8$hasq2_25,

dfwave8$hasq2_5, dfwave8$hasq2_7, dfwave8$hasq2_12, dfwave8$hasq2_18, dfwave8$hasq2_22,

dfwave8$hasq2_3, dfwave8$hasq2_8, dfwave8$hasq2_13, dfwave8$hasq2_16, dfwave8$hasq2_24)

newdataLSICwave8 <- as.data.frame(newdataLSICwave8)

names(newdataLSICwave8) <- c("Item_1", "Item_4", "Item_9", "Item_17", "Item_20",

"Item_6", "Item_11", "Item_14", "Item_19", "Item_23",

"Item_2", "Item_10", "Item_15", "Item_21", "Item_25",

"Item_5", "Item_7", "Item_12", "Item_18", "Item_22",

"Item_3", "Item_8", "Item_13", "Item_16", "Item_24")

newdataLSICwave8[newdataLSICwave8 == -9]<- NA

newdataLSICwave8[newdataLSICwave8 == -6]<- NA

newdataLSICwave8[newdataLSICwave8 == -3]<- NA

newdataLSICwave8[newdataLSICwave8 == -4]<- NA

newdataLSICwave8[newdataLSICwave8 == -2]<- NA

newdataLSICwave8 <- reverse.code(keysSDQ, newdataLSICwave8)

newdataLSICwave8 <- as.data.frame(newdataLSICwave8)

newdatasetLSICwave8cc <- newdataLSICwave8[complete.cases(newdataLSICwave8),]

newdatasetLSICwave8 <- newdatasetLSICwave8cc

names(newdatasetLSICwave8) <- c("considerate", "shares", "caring", "kind", "helps",

"solitary", "friend", "popular", "bullied", "adults",

"restless", "fidgety", "distractible", "reflective", "persistent",

"tempers", "obedient", "fights", "lies", "steals",

"somatic", "worries", "unhappy", "clingy", "fears")

##############################

### Preparing for analysis ###

##############################

CorMatLSICwave8 <- qgraph::cor_auto(newdatasetLSICwave8[,1:25], npn.SKEPTIC=FALSE)

fivescalesLSICwave8 <- list("Prosocial" = c(1:5), "Hyper"=c(6:10), "Emotion" = c(11:15),

"Conduct"=c(16:20), "Peer"=c(21:25))

CorFinLSICwave8 <- CorMatLSICwave8

########################

### Network Analysis ###

########################

#Network estimation and plot#

network1LSICwave8 <- EBICglasso(CorFinLSICwave8, n=nrow(newdatasetLSICwave8))

png("networkLSICwave8.png", res=300, height=5000, width=5000)

networkplotLSICwave8 <- qgraph(network1LSICwave8, layout="spring", cut=0,

theme="colorblind", vsize=7.5, esize=18,

legend=FALSE, groups=fivescales,

labels=colnames(newdataset[1:25]))

dev.off()

#Dimensionality#

ndimLSICwave8 <- EGAnet::EGA(CorFinLSICwave8, n=nrow(newdatasetLSICwave8))

bootdimenLSICwave8 <- EGAnet::bootEGA(newdatasetLSICwave8[,1:25], model="glasso", n=2500, ncores=2)

bootdimenLSICwave8$summary.table

bootdimenLSICwave8$frequency

png("networkLSICwave8EGA.png", res=300, height=5000, width=5000)

ndimplotLSICwave8 <- plot(ndimLSICwave8, theme="colorblind", vsize=7.5, esize=18, layout="spring", legend=FALSE,

cut=0, labels=colnames(newdataset[1:25]))

dev.off()

dimstabLSICwave8 <- dimStability(bootdimenLSICwave8, orig.wc =ndimLSICwave8$wc)

###########################

### REDUNDANCY ANALYSIS ###

###########################

# Round 1 #

redLSICWave8 <- EGAnet::UVA(newdatasetLSICwave8[,1:25], method="wTO", type="adapt", reduce=FALSE,

reduce.method="latent", adhoc=FALSE)

head(redLSICWave8$redundancy$descriptives$centralTendency)

redLSICWave8 <- EGAnet::UVA(newdatasetLSICwave8[,1:25], method="wTO", type="adapt", reduce=TRUE,

reduce.method="latent", adhoc=TRUE)

egaredLSICWave8 <- EGA(redLSICWave8$reduced$data, algorithm = "walktrap")

bootdimenLSACegaredLSICWave8 <- EGAnet::bootEGA(redLSICWave8$reduced$data, model="glasso", n=2500, ncores=2) #bootstraps the number of dimensions

bootdimenLSACegaredLSICWave8$summary.table #Summary table

bootdimenLSACegaredLSICWave8$frequency #Frequency dimensions [3 dim - 0.6688]

###################################

### DETERMINE FACTOR OR NETWORK ###

###################################

# Fit of the factor model 5-factor structure #

tradSDQnewdatasetLSICwave8 <- cfa(tradSDQ, sample.cov =CorFinLSICwave8,

sample.nobs =nrow(newdatasetLSICwave8[,1:25]),

estimator = "ML",

std.lv=TRUE)

round(c(fitmeasures(tradSDQnewdatasetLSICwave8)[3:7],

fitmeasures(tradSDQnewdatasetLSICwave8)[9],

fitmeasures(tradSDQnewdatasetLSICwave8)[23:25],

fitmeasures(tradSDQnewdatasetLSICwave8)[29]), digits=3)

EGAnet::tefi(CorFinLSICwave8,tradSDQstruc) #Lower values suggest better fit

# Fit of the factor model 3-factor structure #

threeSDQnewdatasetLSICwave8 <- cfa(threeSDQ, sample.cov =CorFinLSICwave8,

sample.nobs =nrow(newdatasetLSICwave8[,1:25]),

estimator = "ML",

std.lv=TRUE)

round(c(fitmeasures(threeSDQnewdatasetLSICwave8)[3:7],

fitmeasures(threeSDQnewdatasetLSICwave8)[9],

fitmeasures(threeSDQnewdatasetLSICwave8)[23:25],

fitmeasures(threeSDQnewdatasetLSICwave8)[29]), digits=3)

EGAnet::tefi(CorFinLSICwave8,threeSDQstruc) #Lower values suggest better fit

# Fit of the proposed 4-factor structure #

propSDQnewdatasetLSICwave8 <- cfa(propSDQ, sample.cov =CorFinLSICwave8,

sample.nobs =nrow(newdatasetLSICwave8[,1:25]),

estimator = "ML",

std.lv=TRUE)

round(c(fitmeasures(propSDQnewdatasetLSICwave8)[3:7],

fitmeasures(propSDQnewdatasetLSICwave8)[9],

fitmeasures(propSDQnewdatasetLSICwave8)[23:25],

fitmeasures(propSDQnewdatasetLSICwave8)[29]), digits=3)

EGAnet::tefi(CorFinLSICwave8,propSDQstruc) #Lower values suggest better fit

# Fit of the network model #

adj <- 1*(network1LSICwave8!=0)

nwModelcross <- ggm(covs = CorFinLSICwave8, corinput=TRUE,

omega = adj,

nobs = nrow(newdatasetLSICwave8[,1:25]))

results_nwModelcross <- nwModelcross %>% runmodel

fitnetwork8 <- fit(results_nwModelcross)

fitnetwork8[c(7,9:11,21:24),c(1:2)] %>%

mutate_if(is.numeric, round, digits=3)

EGAnet::tefi(CorFinLSICwave8,ndimLSICwave8$wc) #Lower values suggest better fit

#########################

### UNCONSTRAINED CFA ###

#########################

#CFA (unrestricted) 3-factor structure

threeSDQunres_efa <- fa(newdatasetLSICwave8, cor="poly", nfactors=3, rotate = "geominQ", fm = 'ml', delta = .5)

threeSDQunres_loadings <- data.table(matrix(round(threeSDQunres_efa$loadings, 3),

nrow = 25, ncol = 3))

names(threeSDQunres_loadings) <- c("F1","F2","F3")

threeSDQunres_loadings$item <- paste0(colnames(newdatasetLSICwave8))

threeSDQunres_loadings <- melt(threeSDQunres_loadings, "item", variable.name = "latent")

anchors <- NA

for (l in 1:length(unique(threeSDQunres_loadings$latent))) {

anchors[l] <- threeSDQunres_loadings[threeSDQunres_loadings$value==max(threeSDQunres_loadings$value[threeSDQunres_loadings$latent==unique(threeSDQunres_loadings$latent)[l]]),][[1]]

}

#make model

threeSDQunres_model <- make_esem_model(threeSDQunres_loadings, anchors)

#print model

writeLines(threeSDQunres_model)

threeSDQunres_fit8 <- cfa(threeSDQunres_model,sample.cov =CorFinLSICwave8,

sample.nobs =nrow(newdatasetLSICwave8[,1:25]), std.lv=T, estimator = "ML")

summary(threeSDQunres_fit8, fit.measures = T, standardized = T)

round(c(fitmeasures(threeSDQunres_fit8)[3:7],

fitmeasures(threeSDQunres_fit8)[9],

fitmeasures(threeSDQunres_fit8)[23:25],

fitmeasures(threeSDQunres_fit8)[29]), digits=3)

#Get simple structure for TEFIvn

structurethreeSDQunres<-NA

for (l in 1:length(colnames(newdatasetLSICwave8[,1:25]))){

structurethreeSDQunres[l]=substr(names(which(coef(threeSDQunres_fit8)==

max(coef(threeSDQunres_fit8)[1:(which(names(coef(threeSDQunres_fit8))=="considerate~~considerate")-1)]

[str_detect(names(coef(threeSDQunres_fit8)

[1:(which(names(coef(threeSDQunres_fit8))=="considerate~~considerate")-1)]),

colnames(newdatasetLSICwave8[,1:25])[l])==TRUE]))),

start=2, stop=2)

}

structurethreeSDQunres <- as.numeric(structurethreeSDQunres)

names(structurethreeSDQunres) <- colnames(newdatasetLSICwave8[,1:25])

unique(structurethreeSDQunres)

TEFIvn8threeunres <- EGAnet::tefi(newdatasetLSICwave8[,1:25],structurethreeSDQunres) #Lower values suggest better fit

TEFIvn8threeunres

#CFA (unrestricted) 5-factor structure

tradSDQunres_efa <- fa(newdatasetLSICwave8, cor="poly", nfactors=5, rotate = "geominQ", fm = 'ml', delta = .5)

tradSDQunres_loadings <- data.table(matrix(round(tradSDQunres_efa$loadings, 5),

nrow = 25, ncol = 5))

names(tradSDQunres_loadings) <- c("F1","F2","F3", "F4", "F5")

tradSDQunres_loadings$item <- paste0(colnames(newdatasetLSICwave8))

tradSDQunres_loadings <- melt(tradSDQunres_loadings, "item", variable.name = "latent")

anchors <- NA

for (l in 1:length(unique(tradSDQunres_loadings$latent))) {

anchors[l] <- tradSDQunres_loadings[tradSDQunres_loadings$value==max(tradSDQunres_loadings$value[tradSDQunres_loadings$latent==unique(tradSDQunres_loadings$latent)[l]]),][[1]]

}

names(anchors) <- c("F1","F2","F3", "F4", "F5")

#make model

tradSDQunres_model <- make_esem_model(tradSDQunres_loadings, anchors)

#print model

writeLines(tradSDQunres_model)

tradSDQunres_fit8 <- cfa(tradSDQunres_model, sample.cov =CorFinLSICwave8,

sample.nobs =nrow(newdatasetLSICwave8[,1:25]), std.lv=T, estimator = "ML")

summary(tradSDQunres_fit8, fit.measures = T, standardized = T)

round(c(fitmeasures(tradSDQunres_fit8)[3:7],

fitmeasures(tradSDQunres_fit8)[9],

fitmeasures(tradSDQunres_fit8)[23:25],

fitmeasures(tradSDQunres_fit8)[29]), digits=3)

#Get simple structure for TEFIvn

structuretradSDQunres<-NA

for (l in 1:length(colnames(newdatasetLSICwave8[,1:25]))){

structuretradSDQunres[l]=substr(names(which(coef(tradSDQunres_fit8)==

max(coef(tradSDQunres_fit8)[1:(which(names(coef(tradSDQunres_fit8))=="considerate~~considerate")-1)]

[str_detect(names(coef(tradSDQunres_fit8)

[1:(which(names(coef(tradSDQunres_fit8))=="considerate~~considerate")-1)]),

colnames(newdatasetLSICwave8[,1:25])[l])==TRUE]))),

start=2, stop=2)

}

structuretradSDQunres <- as.numeric(structuretradSDQunres)

names(structuretradSDQunres) <- colnames(newdatasetLSICwave8[,1:25])

unique(structuretradSDQunres)

TEFIvn8tradunres <- EGAnet::tefi(newdatasetLSICwave8[,1:25],structuretradSDQunres) #Lower values suggest better fit

TEFIvn8tradunres

###############################

### LSIC - WAVE 10B ###

### CHILDREN AGED 4 to 10 ###

###############################

###############################

dfwave10 <- read_sav("lsiccombinedw10_100c.sav")

dfwave10 <- as.data.frame(dfwave10)

dfwave10B <- dfwave10[dfwave10$jachtype==1,]

dfwave10B$age <- ifelse(dfwave10B$jascagem==-9,dfwave10B$jascagem,floor(dfwave10B$jascagem/12))

dfwave10B <- dfwave10B[!(dfwave10B$age==11),]

c(mean(dfwave10B$age),sd(dfwave10B$age),min(subset(dfwave10B$age,dfwave10B$age!=-9)),max(dfwave10B$age))

(length(which(dfwave10B$age==-9))/nrow(dfwave10B))*100

newdataLSICwave10B <- cbind(dfwave10B$jasq2_1, dfwave10B$jasq2_4, dfwave10B$jasq2_9, dfwave10B$jasq2_17, dfwave10B$jasq2_20,

dfwave10B$jasq2_6, dfwave10B$jasq2_11, dfwave10B$jasq2_14, dfwave10B$jasq2_19, dfwave10B$jasq2_23,

dfwave10B$jasq2_2, dfwave10B$jasq2_10, dfwave10B$jasq2_15, dfwave10B$jasq2_21, dfwave10B$jasq2_25,

dfwave10B$jasq2_5, dfwave10B$jasq2_7, dfwave10B$jasq2_12, dfwave10B$jasq2_18, dfwave10B$jasq2_22,

dfwave10B$jasq2_3, dfwave10B$jasq2_8, dfwave10B$jasq2_13, dfwave10B$jasq2_16, dfwave10B$jasq2_24)

newdataLSICwave10B <- as.data.frame(newdataLSICwave10B)

names(newdataLSICwave10B) <- c("Item_1", "Item_4", "Item_9", "Item_17", "Item_20",

"Item_6", "Item_11", "Item_14", "Item_19", "Item_23",

"Item_2", "Item_10", "Item_15", "Item_21", "Item_25",

"Item_5", "Item_7", "Item_12", "Item_18", "Item_22",

"Item_3", "Item_8", "Item_13", "Item_16", "Item_24")

newdataLSICwave10B[newdataLSICwave10B == -9]<- NA

newdataLSICwave10B[newdataLSICwave10B == -6]<- NA

newdataLSICwave10B[newdataLSICwave10B == -3]<- NA

newdataLSICwave10B[newdataLSICwave10B == -4]<- NA

newdataLSICwave10B[newdataLSICwave10B == -2]<- NA

newdataLSICwave10B <- reverse.code(keysSDQ, newdataLSICwave10B)

newdataLSICwave10B <- as.data.frame(newdataLSICwave10B)

newdatasetLSICwave10Bcc <- newdataLSICwave10B[complete.cases(newdataLSICwave10B),]

newdatasetLSICwave10B <- newdatasetLSICwave10Bcc

names(newdatasetLSICwave10B) <- c("considerate", "shares", "caring", "kind", "helps",

"solitary", "friend", "popular", "bullied", "adults",

"restless", "fidgety", "distractible", "reflective", "persistent",

"tempers", "obedient", "fights", "lies", "steals",

"somatic", "worries", "unhappy", "clingy", "fears")

##############################

### Preparing for analysis ###

##############################

CorMatLSICwave10B <- qgraph::cor_auto(newdatasetLSICwave10B[,1:25], npn.SKEPTIC=FALSE)

fivescalesLSICwave10B <- list("Prosocial" = c(1:5), "Hyper"=c(6:10), "Emotion" = c(11:15),

"Conduct"=c(16:20), "Peer"=c(21:25))

CorFinLSICwave10B <- CorMatLSICwave10B

########################

### Network Analysis ###

########################

#Network estimation and plot#

network1LSICwave10B <- EBICglasso(CorFinLSICwave10B, n=nrow(newdatasetLSICwave10B))

png("networkLSICwave10B.png", res=300, height=5000, width=5000)

networkplotLSICwave10B <- qgraph(network1LSICwave10B, layout="spring", cut=0,

theme="colorblind", vsize=7.5, esize=18,

legend=FALSE, groups=fivescales,

labels=colnames(newdataset[1:25]))

dev.off()

#Dimensionality#

ndimLSICwave10B <- EGAnet::EGA(CorFinLSICwave10B, n=nrow(newdatasetLSICwave10B))

newdatasetLSICwave10B <- as.data.frame(newdatasetLSICwave10B)

head(newdatasetLSICwave10B)

bootdimenLSICwave10B <- EGAnet::bootEGA(newdatasetLSICwave10B, model="glasso", n=2500, ncores=2)

bootdimenLSICwave10B$summary.table

bootdimenLSICwave10B$frequency

png("networkLSICwave10BEGA.png", res=300, height=5000, width=5000)

ndimplotLSICwave10B <- plot(ndimLSICwave10B, theme="colorblind", vsize=7.5, esize=18, layout="spring", legend=FALSE,

cut=0, labels=colnames(newdataset[1:25]))

dev.off()

dimstabLSICwave10B <- dimStability(bootdimenLSICwave10B, orig.wc =ndimLSICwave10B$wc)

###########################

### REDUNDANCY ANALYSIS ###

###########################

# Round 1 #

redLSICWave10B <- EGAnet::UVA(newdatasetLSICwave10B[,1:25], method="wTO", type="adapt", reduce=FALSE,

reduce.method="latent", adhoc=FALSE)

head(redLSICWave10B$redundancy$descriptives$centralTendency)

redLSICWave10B <- EGAnet::UVA(newdatasetLSICwave10B[,1:25], method="wTO", type="adapt", reduce=TRUE,

reduce.method="latent", adhoc=TRUE)

egaredLSICWave10B <- EGA(redLSICWave10B$reduced$data, algorithm = "walktrap")

bootdimenLSACegaredLSICWave10B <- EGAnet::bootEGA(redLSICWave10B$reduced$data, model="glasso", n=2500, ncores=2) #bootstraps the number of dimensions

bootdimenLSACegaredLSICWave10B$summary.table #Summary table

bootdimenLSACegaredLSICWave10B$frequency #Frequency dimensions [2 dim - 0.7916]

###################################

### DETERMINE FACTOR OR NETWORK ###

###################################

# Fit of the factor model 5-factor structure #

tradSDQnewdatasetLSICwave10B <- cfa(tradSDQ, sample.cov =CorFinLSICwave10B,

sample.nobs =nrow(newdatasetLSICwave10B[,1:25]),

estimator = "ML",

std.lv=TRUE)

round(c(fitmeasures(tradSDQnewdatasetLSICwave10B)[3:7],

fitmeasures(tradSDQnewdatasetLSICwave10B)[9],

fitmeasures(tradSDQnewdatasetLSICwave10B)[23:25],

fitmeasures(tradSDQnewdatasetLSICwave10B)[29]), digits=3)

EGAnet::tefi(CorFinLSICwave10B,tradSDQstruc) #Lower values suggest better fit

# Fit of the factor model 3-factor structure #

threeSDQnewdatasetLSICwave10B <- cfa(threeSDQ, sample.cov =CorFinLSICwave10B,

sample.nobs =nrow(newdatasetLSICwave10B[,1:25]),

estimator = "ML",

std.lv=TRUE)

round(c(fitmeasures(threeSDQnewdatasetLSICwave10B)[3:7],

fitmeasures(threeSDQnewdatasetLSICwave10B)[9],

fitmeasures(threeSDQnewdatasetLSICwave10B)[23:25],

fitmeasures(threeSDQnewdatasetLSICwave10B)[29]), digits=3)

EGAnet::tefi(CorFinLSICwave10B,threeSDQstruc) #Lower values suggest better fit

# Fit of the proposed 4-factor structure #

propSDQnewdatasetLSICwave10B <- cfa(propSDQ, sample.cov =CorFinLSICwave10B,

sample.nobs =nrow(newdatasetLSICwave10B[,1:25]),

estimator = "ML",

std.lv=TRUE)

round(c(fitmeasures(propSDQnewdatasetLSICwave10B)[3:7],

fitmeasures(propSDQnewdatasetLSICwave10B)[9],

fitmeasures(propSDQnewdatasetLSICwave10B)[23:25],

fitmeasures(propSDQnewdatasetLSICwave10B)[29]), digits=3)

EGAnet::tefi(CorFinLSICwave10B,propSDQstruc) #Lower values suggest better fit

# Fit of the network model #

adj <- 1*(network1LSICwave10B!=0)

nwModelcross <- ggm(covs = CorFinLSICwave10B, corinput=TRUE,

omega = adj,

nobs = nrow(newdatasetLSICwave10B[,1:25]))

results_nwModelcross <- nwModelcross %>% runmodel

fitnetwork10B <- fit(results_nwModelcross)

fitnetwork10B[c(7,9:11,21:24),c(1:2)] %>%

mutate_if(is.numeric, round, digits=3)

EGAnet::tefi(CorFinLSICwave10B,ndimLSICwave10B$wc) #Lower values suggest better fit

#########################

### UNCONSTRAINED CFA ###

#########################

#CFA (unrestricted) 3-factor structure

threeSDQunres_efa <- fa(newdatasetLSICwave10B, cor="poly", nfactors=3, rotate = "geominQ", fm = 'ml', delta = .5)

threeSDQunres_loadings <- data.table(matrix(round(threeSDQunres_efa$loadings, 3),

nrow = 25, ncol = 3))

names(threeSDQunres_loadings) <- c("F1","F2","F3")

threeSDQunres_loadings$item <- paste0(colnames(newdatasetLSICwave10B))

threeSDQunres_loadings <- melt(threeSDQunres_loadings, "item", variable.name = "latent")

anchors <- NA

for (l in 1:length(unique(threeSDQunres_loadings$latent))) {

anchors[l] <- threeSDQunres_loadings[threeSDQunres_loadings$value==max(threeSDQunres_loadings$value[threeSDQunres_loadings$latent==unique(threeSDQunres_loadings$latent)[l]]),][[1]]

}

#make model

threeSDQunres_model <- make_esem_model(threeSDQunres_loadings, anchors)

#print model

writeLines(threeSDQunres_model)

threeSDQunres_fit10B <- cfa(threeSDQunres_model, sample.cov =CorFinLSICwave10B,

sample.nobs =nrow(newdatasetLSICwave10B[,1:25]), std.lv=T, estimator = "ML")

summary(threeSDQunres_fit10B, fit.measures = T, standardized = T)

round(c(fitmeasures(threeSDQunres_fit10B)[3:7],

fitmeasures(threeSDQunres_fit10B)[9],

fitmeasures(threeSDQunres_fit10B)[23:25],

fitmeasures(threeSDQunres_fit10B)[29]), digits=3)

#Get simple structure for TEFIvn

structurethreeSDQunres<-NA

for (l in 1:length(colnames(newdatasetLSICwave10B[,1:25]))){

structurethreeSDQunres[l]=substr(names(which(coef(threeSDQunres_fit10B)==

max(coef(threeSDQunres_fit10B)[1:(which(names(coef(threeSDQunres_fit10B))=="considerate~~considerate")-1)]

[str_detect(names(coef(threeSDQunres_fit10B)

[1:(which(names(coef(threeSDQunres_fit10B))=="considerate~~considerate")-1)]),

colnames(newdatasetLSICwave10B[,1:25])[l])==TRUE]))),

start=2, stop=2)

}

structurethreeSDQunres <- as.numeric(structurethreeSDQunres)

names(structurethreeSDQunres) <- colnames(newdatasetLSICwave10B[,1:25])

unique(structurethreeSDQunres)

TEFIvn3kthreeunres <- EGAnet::tefi(newdatasetLSICwave10B[,1:25],structurethreeSDQunres) #Lower values suggest better fit

TEFIvn3kthreeunres

#CFA (unrestricted) 5-factor structure

tradSDQunres_efa <- fa(newdatasetLSICwave10B, cor="poly", nfactors=5, rotate = "geominQ", fm = 'ml', delta = .5)

tradSDQunres_loadings <- data.table(matrix(round(tradSDQunres_efa$loadings, 5),

nrow = 25, ncol = 5))

names(tradSDQunres_loadings) <- c("F1","F2","F3", "F4", "F5")

tradSDQunres_loadings$item <- paste0(colnames(newdatasetLSICwave10B))

tradSDQunres_loadings <- melt(tradSDQunres_loadings, "item", variable.name = "latent")

anchors <- NA

for (l in 1:length(unique(tradSDQunres_loadings$latent))) {

anchors[l] <- tradSDQunres_loadings[tradSDQunres_loadings$value==max(tradSDQunres_loadings$value[tradSDQunres_loadings$latent==unique(tradSDQunres_loadings$latent)[l]]),][[1]]

}

names(anchors) <- c("F1","F2","F3", "F4", "F5")

#make model

tradSDQunres_model <- make_esem_model(tradSDQunres_loadings, anchors)

#print model

writeLines(tradSDQunres_model)

tradSDQunres_fit10B <- cfa(tradSDQunres_model,sample.cov =CorFinLSICwave10B,

sample.nobs =nrow(newdatasetLSICwave10B[,1:25]), std.lv=T, estimator = "ML")

summary(tradSDQunres_fit10B, fit.measures = T, standardized = T)

round(c(fitmeasures(tradSDQunres_fit10B)[3:7],

fitmeasures(tradSDQunres_fit10B)[9],

fitmeasures(tradSDQunres_fit10B)[23:25],

fitmeasures(tradSDQunres_fit10B)[29]), digits=3)

#Get simple structure for TEFIvn

structuretradSDQunres<-NA

for (l in 1:length(colnames(newdatasetLSICwave10B[,1:25]))){

structuretradSDQunres[l]=substr(names(which(coef(tradSDQunres_fit10B)==

max(coef(tradSDQunres_fit10B)[1:(which(names(coef(tradSDQunres_fit10B))=="considerate~~considerate")-1)]

[str_detect(names(coef(tradSDQunres_fit10B)

[1:(which(names(coef(tradSDQunres_fit10B))=="considerate~~considerate")-1)]),

colnames(newdatasetLSICwave10B[,1:25])[l])==TRUE]))),

start=2, stop=2)

}

structuretradSDQunres <- as.numeric(structuretradSDQunres)

names(structuretradSDQunres) <- colnames(newdatasetLSICwave10B[,1:25])

unique(structuretradSDQunres)

TEFIvn2ktradunres <- EGAnet::tefi(newdatasetLSICwave10B[,1:25],structuretradSDQunres) #Lower values suggest better fit

TEFIvn2ktradunres

################################

### BASIC DESCRIPTIVE TABLES ###

################################

descitem <- cbind(

rbind(do.call(rbind.data.frame, lapply(newdatasetLSICwave3K, mean)), mean(rowSums(newdatasetLSICwave3K[,1:5])),

mean(rowSums(newdatasetLSICwave3K[,6:10])), mean(rowSums(newdatasetLSICwave3K[,11:15])),

mean(rowSums(newdatasetLSICwave3K[,16:20])), mean(rowSums(newdatasetLSICwave3K[,21:25]))),

rbind(do.call(rbind.data.frame, lapply(newdatasetLSICwave4K, mean)), mean(rowSums(newdatasetLSICwave4K[,1:5])),

mean(rowSums(newdatasetLSICwave4K[,6:10])), mean(rowSums(newdatasetLSICwave4K[,11:15])),

mean(rowSums(newdatasetLSICwave4K[,16:20])), mean(rowSums(newdatasetLSICwave4K[,21:25]))),

rbind(do.call(rbind.data.frame, lapply(newdatasetLSICwave6, mean)), mean(rowSums(newdatasetLSICwave6[,1:5])),

mean(rowSums(newdatasetLSICwave6[,6:10])), mean(rowSums(newdatasetLSICwave6[,11:15])),

mean(rowSums(newdatasetLSICwave6[,16:20])), mean(rowSums(newdatasetLSICwave6[,21:25]))),

rbind(do.call(rbind.data.frame, lapply(newdatasetLSICwave8, mean)), mean(rowSums(newdatasetLSICwave8[,1:5])),

mean(rowSums(newdatasetLSICwave8[,6:10])), mean(rowSums(newdatasetLSICwave8[,11:15])),

mean(rowSums(newdatasetLSICwave8[,16:20])), mean(rowSums(newdatasetLSICwave8[,21:25]))),

rbind(do.call(rbind.data.frame, lapply(newdatasetLSICwave10B, mean)), mean(rowSums(newdatasetLSICwave10B[,1:5])),

mean(rowSums(newdatasetLSICwave10B[,6:10])), mean(rowSums(newdatasetLSICwave10B[,11:15])),

mean(rowSums(newdatasetLSICwave10B[,16:20])), mean(rowSums(newdatasetLSICwave10B[,21:25]))),

rbind(do.call(rbind.data.frame, lapply(newdataset, mean)), mean(rowSums(newdataset[,1:5])),

mean(rowSums(newdataset[,6:10])), mean(rowSums(newdataset[,11:15])),

mean(rowSums(newdataset[,16:20])), mean(rowSums(newdataset[,21:25]))))

colnames(descitem) <- c("LSIC Wave 3K", "LSIC Wave 4K", "LSIC Wave 6", "LSIC Wave 8", "LSIC Wave 10B", "SAABC Wave 5")

descitem <- cbind(Row.Names = c(colnames(newdatasetLSICwave3K), "Prosocial Behaviour",

"Peer Problems", "Hyperactivity", "Conduct Problems", "Emotional Problems"), descitem)

descitem <- round(descitem[,2:7], digits=2)

descitemsd <- cbind(

rbind(do.call(rbind.data.frame, lapply(newdatasetLSICwave3K, sd)), sd(rowSums(newdatasetLSICwave3K[,1:5])),

sd(rowSums(newdatasetLSICwave3K[,6:10])), sd(rowSums(newdatasetLSICwave3K[,11:15])),

sd(rowSums(newdatasetLSICwave3K[,16:20])), sd(rowSums(newdatasetLSICwave3K[,21:25]))),

rbind(do.call(rbind.data.frame, lapply(newdatasetLSICwave4K, sd)), sd(rowSums(newdatasetLSICwave4K[,1:5])),

sd(rowSums(newdatasetLSICwave4K[,6:10])), sd(rowSums(newdatasetLSICwave4K[,11:15])),

sd(rowSums(newdatasetLSICwave4K[,16:20])), sd(rowSums(newdatasetLSICwave4K[,21:25]))),

rbind(do.call(rbind.data.frame, lapply(newdatasetLSICwave6, sd)), sd(rowSums(newdatasetLSICwave6[,1:5])),

sd(rowSums(newdatasetLSICwave6[,6:10])), sd(rowSums(newdatasetLSICwave6[,11:15])),

sd(rowSums(newdatasetLSICwave6[,16:20])), sd(rowSums(newdatasetLSICwave6[,21:25]))),

rbind(do.call(rbind.data.frame, lapply(newdatasetLSICwave8, sd)), sd(rowSums(newdatasetLSICwave8[,1:5])),

sd(rowSums(newdatasetLSICwave8[,6:10])), sd(rowSums(newdatasetLSICwave8[,11:15])),

sd(rowSums(newdatasetLSICwave8[,16:20])), sd(rowSums(newdatasetLSICwave8[,21:25]))),

rbind(do.call(rbind.data.frame, lapply(newdatasetLSICwave10B, sd)), sd(rowSums(newdatasetLSICwave10B[,1:5])),

sd(rowSums(newdatasetLSICwave10B[,6:10])), sd(rowSums(newdatasetLSICwave10B[,11:15])),

sd(rowSums(newdatasetLSICwave10B[,16:20])), sd(rowSums(newdatasetLSICwave10B[,21:25]))),

rbind(do.call(rbind.data.frame, lapply(newdataset, sd)), sd(rowSums(newdataset[,1:5])),

sd(rowSums(newdataset[,6:10])), sd(rowSums(newdataset[,11:15])),

sd(rowSums(newdataset[,16:20])), sd(rowSums(newdataset[,21:25]))))

colnames(descitemsd) <- c("LSIC Wave 3K (SD)", "LSIC Wave 4K (SD)", "LSIC Wave 6 (SD)", "LSIC Wave 8 (SD)", "LSIC Wave 10B (SD)",

"SAABC Wave 5 (SD)")

descitemsd <- cbind(Row.Names = c(colnames(newdatasetLSICwave3K), "Prosocial Behaviour",

"Peer Problems", "Hyperactivity", "Conduct Problems", "Emotional Problems"), descitemsd)

descitemsd <- round(descitemsd[,2:7], digits=2)

descitemall <- cbind.data.frame(paste(round(descitem[,1],digits=2), " (", round(descitemsd[,1],digits=2), ") ", sep = ""),

paste(round(descitem[,2],digits=2), " (", round(descitemsd[,2],digits=2), ") ", sep = ""),

paste(round(descitem[,3],digits=2), " (", round(descitemsd[,3],digits=2), ") ", sep = ""),

paste(round(descitem[,4],digits=2), " (", round(descitemsd[,4],digits=2), ") ", sep = ""),

paste(round(descitem[,5],digits=2), " (", round(descitemsd[,5],digits=2), ") ", sep = ""),

paste(round(descitem[,6],digits=2), " (", round(descitemsd[,6],digits=2), ") ", sep = ""))

colnames(descitemall) <- c("LSIC Wave 3K", "LSIC Wave 4K", "LSIC Wave 6", "LSIC Wave 8", "LSIC Wave 10B", "SAABC Wave 5")

descitemall <- cbind(Row.Names = c(colnames(newdatasetLSICwave3K), "Prosocial Behaviour",

"Peer Problems", "Hyperactivity", "Conduct Problems", "Emotional Problems"), descitemall)

options(max.print=1000000)

write(paste(utils::capture.output(descitemall),

collapse = "\n"), file = "DescriptiveAllDataset.csv")

###################

### FINAL PLOTS ###

###################

plotnormal <- averageLayout(networkplot, networkplotLSICwave3K, networkplotLSICwave4K,

networkplotLSICwave6, networkplotLSICwave8, networkplotLSICwave10B)

png("networkBTTnormal.png", res=300, height=5000, width=5000)

networkBTTnormal <- qgraph(network1, layout=plotnormal, cut=0,

theme="colorblind", vsize=7.5, esize=18,

legend=FALSE, groups=fivescales,

labels=colnames(newdataset[1:25]))

dev.off()

png("networkLSICwave3Knormal.png", res=300, height=5000, width=5000)

networkplotLSICwave3Knormal <- qgraph(network1LSICwave3K, layout=plotnormal, cut=0,

theme="colorblind", vsize=7.5, esize=18,

legend=FALSE, groups=fivescales,

labels=colnames(newdataset[1:25]))

dev.off()

png("networkLSICwave4Knormal.png", res=300, height=5000, width=5000)

networkplotLSICwave4Knormal <- qgraph(network1LSICwave4K, layout=plotnormal, cut=0,

theme="colorblind", vsize=7.5, esize=18,

legend=FALSE, groups=fivescales,

labels=colnames(newdataset[1:25]))

dev.off()

png("networkLSICwave6normal.png", res=300, height=5000, width=5000)

networkplotLSICwave6normal <- qgraph(network1LSICwave6, layout=plotnormal, cut=0,

theme="colorblind", vsize=7.5, esize=18,

legend=FALSE, groups=fivescales,

labels=colnames(newdataset[1:25]))

dev.off()

png("networkLSICwave8normal.png", res=300, height=5000, width=5000)

networkplotLSICwave8normal <- qgraph(network1LSICwave8, layout=plotnormal, cut=0,

theme="colorblind", vsize=7.5, esize=18,

legend=FALSE, groups=fivescales,

labels=colnames(newdataset[1:25]))

dev.off()

png("networkLSICwave10Bnormal.png", res=300, height=5000, width=5000)

networkplotLSICwave10Bnormal <- qgraph(network1LSICwave10B, layout=plotnormal, cut=0,

theme="colorblind", vsize=7.5, esize=18,

legend=FALSE, groups=fivescales,

labels=colnames(newdataset[1:25]))

dev.off()

########################

### FINAL RESULTS ###

########################

ndim[1]

ndimLSICwave3K[1]

ndimLSICwave4K[1]

ndimLSICwave6[1]

ndimLSICwave8[1]

ndimLSICwave10B[1]

bootdimen$frequency

bootdimenLSICwave3K$frequency

bootdimenLSICwave4K$frequency

bootdimenLSICwave6$frequency

bootdimenLSICwave8$frequency

bootdimenLSICwave10B$frequency

ndimr <- ndim

ndimLSICwave3Kr <- ndimLSICwave3K

ndimLSICwave4Kr<- ndimLSICwave4K

ndimLSICwave6r <- ndimLSICwave6

ndimLSICwave8r <- ndimLSICwave8

ndimLSICwave10Br <- ndimLSICwave10B

ndimLSICwave3Kr$dim.variables$dimension <- car::recode(ndimLSICwave3K$dim.variables$dimension, "3='1'; 2='3'; 1='2'")

ndimLSICwave4Kr$dim.variables$dimension <- car::recode(ndimLSICwave4K$dim.variables$dimension, "3='2'; 2='3'")

ndimLSICwave6r$dim.variables$dimension <- car::recode(ndimLSICwave6$dim.variables$dimension, "2='3'; 3='2'")

ndimLSICwave8r$dim.variables$dimension <- car::recode(ndimLSICwave8$dim.variables$dimension, "3='2'; 2='3'")

ndimLSICwave10Br$dim.variables$dimension <- car::recode(ndimLSICwave10B$dim.variables$dimension, "1='3'; 2='1'; 3='2'")

itemdimr <- join_all(list(ndimr$dim.variables,

ndimLSICwave3Kr$dim.variables,

ndimLSICwave4Kr$dim.variables,

ndimLSICwave6r$dim.variables,

ndimLSICwave8r$dim.variables,

ndimLSICwave10Br$dim.variables), by = "items")

itemdimr

########################

### NETWORK LOADINGS ###

########################

loadings3K <- EGAnet::net.loads(ndimLSICwave3K)

loadings3K <- loadings3K$std

loadings3K <- setDT(loadings3K, keep.rownames = TRUE)[]

loadings3K <- as.data.frame(loadings3K)

loadings3K <- loadings3K[order(match(loadings3K[,1],ordervar)),]

loadings3K[,2:ncol(loadings3K)] <- format(round(loadings3K[,2:ncol(loadings3K)], digits=2), digits=2)

loadings3K[(loadings3K==" NA")|(loadings3K==" NA")] <- ""

loadings4K <- EGAnet::net.loads(ndimLSICwave4K)

loadings4K <- loadings4K$std

loadings4K <- setDT(loadings4K, keep.rownames = TRUE)[]

loadings4K <- as.data.frame(loadings4K)

loadings4K <- loadings4K[order(match(loadings4K[,1],ordervar)),]

loadings4K[,2:ncol(loadings4K)] <- format(round(loadings4K[,2:ncol(loadings4K)], digits=2), digits=2)

loadings4K[(loadings4K==" NA")|(loadings4K==" NA")] <- ""

loadings6 <- EGAnet::net.loads(ndimLSICwave6)

loadings6 <- loadings6$std

loadings6 <- setDT(loadings6, keep.rownames = TRUE)[]

loadings6 <- as.data.frame(loadings6)

loadings6 <- loadings6[order(match(loadings6[,1],ordervar)),]

loadings6[,2:ncol(loadings6)] <- format(round(loadings6[,2:ncol(loadings6)], digits=2), digits=2)

loadings6[(loadings6==" NA")|(loadings6==" NA")] <- ""

loadings8 <- EGAnet::net.loads(ndimLSICwave8)

loadings8 <- loadings8$std

loadings8 <- setDT(loadings8, keep.rownames = TRUE)[]

loadings8 <- as.data.frame(loadings8)

loadings8 <- loadings8[order(match(loadings8[,1],ordervar)),]

loadings8[,2:ncol(loadings8)] <- format(round(loadings8[,2:ncol(loadings8)], digits=2), digits=2)

loadings8[(loadings8==" NA")|(loadings8==" NA")] <- ""

loadings10B <- EGAnet::net.loads(ndimLSICwave10B)

loadings10B <- loadings10B$std

loadings10B <- setDT(loadings10B, keep.rownames = TRUE)[]

loadings10B <- as.data.frame(loadings10B)

loadings10B <- loadings10B[order(match(loadings10B[,1],ordervar)),]

loadings10B[,2:ncol(loadings10B)] <- format(round(loadings10B[,2:ncol(loadings10B)], digits=2), digits=2)

loadings10B[(loadings10B==" NA")|(loadings10B==" NA")] <- ""

loadingsSAABC <- EGAnet::net.loads(ndim)

loadingsSAABC <- loadingsSAABC$std

loadingsSAABC <- setDT(loadingsSAABC, keep.rownames = TRUE)[]

loadingsSAABC <- as.data.frame(loadingsSAABC)

loadingsSAABC <- loadingsSAABC[order(match(loadingsSAABC[,1],ordervar)),]

loadingsSAABC[,2:ncol(loadingsSAABC)] <- format(round(loadingsSAABC[,2:ncol(loadingsSAABC)], digits=2), digits=2)

loadingsSAABC[(loadingsSAABC==" NA")|(loadingsSAABC==" NA")] <- ""

totloadings <- list(loadingsSAABC,

loadings3K,

loadings4K,

loadings6,

loadings8,

loadings10B)

write.table(format(loadingsSAABC, digits=2), file="loadingsSAABC.txt", quote=FALSE, sep=",")

write.table(format(loadings3K, digits=2), file="loadings3k.txt", quote=FALSE, sep=",")

write.table(format(loadings4K, digits=2), file="loadings4k.txt", quote=FALSE, sep=",")

write.table(format(loadings6, digits=2), file="loadings6.txt", quote=FALSE, sep=",")

write.table(format(loadings8, digits=2), file="loadings8.txt", quote=FALSE, sep=",")

write.table(format(loadings10B, digits=2), file="loadings10b.txt", quote=FALSE, sep=",")

####################

### FIT PRINTING ###

####################

write(paste(utils::capture.output(standardizedsolution(tradSDQnewdataset)),

collapse = "\n"), file = "TradSDQSAABC.csv")

write(paste(utils::capture.output(standardizedsolution(threeSDQnewdataset)),

collapse = "\n"), file = "ThreeSDQSAABC.csv")

write(paste(utils::capture.output(standardizedsolution(propSDQnewdataset)),

collapse = "\n"), file = "PropSDQSAABC.csv")

write(paste(utils::capture.output(standardizedsolution(tradSDQunres_fitSAABC)),

collapse = "\n"), file = "UnresTradSDQSAABC.csv")

write(paste(utils::capture.output(standardizedsolution(threeSDQunres_fitSAABC)),

collapse = "\n"), file = "UnresThreeSDQSAABC.csv")

write(paste(utils::capture.output(standardizedsolution(tradSDQnewdatasetLSICwave3K)),

collapse = "\n"), file = "TradSDQ3K.csv")

write(paste(utils::capture.output(standardizedsolution(threeSDQnewdatasetLSICwave3K)),

collapse = "\n"), file = "ThreeSDQ3K.csv")

write(paste(utils::capture.output(standardizedsolution(propSDQnewdatasetLSICwave3K)),

collapse = "\n"), file = "PropSDQ3K.csv")

write(paste(utils::capture.output(standardizedsolution(tradSDQunres_fit3K)),

collapse = "\n"), file = "UnresTradSDQ3K.csv")

write(paste(utils::capture.output(standardizedsolution(threeSDQunres_fit3K)),

collapse = "\n"), file = "UnresThreeSDQ3K.csv")

write(paste(utils::capture.output(standardizedsolution(tradSDQnewdatasetLSICwave4K)),

collapse = "\n"), file = "TradSDQ4K.csv")

write(paste(utils::capture.output(standardizedsolution(threeSDQnewdatasetLSICwave4K)),

collapse = "\n"), file = "ThreeSDQ4K.csv")

write(paste(utils::capture.output(standardizedsolution(propSDQnewdatasetLSICwave4K)),

collapse = "\n"), file = "PropSDQ4K.csv")

write(paste(utils::capture.output(standardizedsolution(tradSDQunres_fit4K)),

collapse = "\n"), file = "UnresTradSDQ4K.csv")

write(paste(utils::capture.output(standardizedsolution(threeSDQunres_fit4K)),

collapse = "\n"), file = "UnresThreeSDQ4K.csv")

write(paste(utils::capture.output(standardizedsolution(tradSDQnewdatasetLSICwave6)),

collapse = "\n"), file = "TradSDQ6.csv")

write(paste(utils::capture.output(standardizedsolution(threeSDQnewdatasetLSICwave6)),

collapse = "\n"), file = "ThreeSDQ6.csv")

write(paste(utils::capture.output(standardizedsolution(propSDQnewdatasetLSICwave6)),

collapse = "\n"), file = "PropSDQ6.csv")

write(paste(utils::capture.output(standardizedsolution(tradSDQunres_fit6)),

collapse = "\n"), file = "UnresTradSDQ6.csv")

write(paste(utils::capture.output(standardizedsolution(threeSDQunres_fit6)),

collapse = "\n"), file = "UnresThreeSDQ6.csv")

write(paste(utils::capture.output(standardizedsolution(tradSDQnewdatasetLSICwave8)),

collapse = "\n"), file = "TradSDQ8.csv")

write(paste(utils::capture.output(standardizedsolution(threeSDQnewdatasetLSICwave8)),

collapse = "\n"), file = "ThreeSDQ8.csv")

write(paste(utils::capture.output(standardizedsolution(propSDQnewdatasetLSICwave8)),

collapse = "\n"), file = "PropSDQ8.csv")

write(paste(utils::capture.output(standardizedsolution(tradSDQunres_fit8)),

collapse = "\n"), file = "UnresTradSDQ8.csv")

write(paste(utils::capture.output(standardizedsolution(threeSDQunres_fit8)),

collapse = "\n"), file = "UnresThreeSDQ8.csv")

write(paste(utils::capture.output(standardizedsolution(tradSDQnewdatasetLSICwave10B)),

collapse = "\n"), file = "TradSDQ10B.csv")

write(paste(utils::capture.output(standardizedsolution(threeSDQnewdatasetLSICwave10B)),

collapse = "\n"), file = "ThreeSDQ10B.csv")

write(paste(utils::capture.output(standardizedsolution(propSDQnewdatasetLSICwave10B)),

collapse = "\n"), file = "PropSDQ10B.csv")

write(paste(utils::capture.output(standardizedsolution(tradSDQunres_fit10B)),

collapse = "\n"), file = "UnresTradSDQ10B.csv")

write(paste(utils::capture.output(standardizedsolution(threeSDQunres_fit10B)),

collapse = "\n"), file = "UnresThreeSDQ10B.csv")

options(scipen=999)

fitnewdataset <- rbind(round(c(fitmeasures(tradSDQnewdataset)[6:8], fitmeasures(tradSDQnewdataset)[25],

fitmeasures(tradSDQnewdataset)[40:43], fitmeasures(tradSDQnewdataset)[50]), digits=3),

round(c(fitmeasures(threeSDQnewdataset)[6:8], fitmeasures(threeSDQnewdataset)[25],

fitmeasures(threeSDQnewdataset)[40:43], fitmeasures(threeSDQnewdataset)[50]), digits=3),

round(c(fitmeasures(propSDQnewdataset)[6:8], fitmeasures(propSDQnewdataset)[25],

fitmeasures(propSDQnewdataset)[40:43], fitmeasures(propSDQnewdataset)[50]), digits=3),

round(c(fitmeasures(tradSDQnewdatasetLSICwave3K)[6:8], fitmeasures(tradSDQnewdatasetLSICwave3K)[25],

fitmeasures(tradSDQnewdatasetLSICwave3K)[40:43], fitmeasures(tradSDQnewdatasetLSICwave3K)[50]), digits=3),

round(c(fitmeasures(threeSDQnewdatasetLSICwave3K)[6:8], fitmeasures(threeSDQnewdatasetLSICwave3K)[25],

fitmeasures(threeSDQnewdatasetLSICwave3K)[40:43], fitmeasures(threeSDQnewdatasetLSICwave3K)[50]), digits=3),

round(c(fitmeasures(propSDQnewdatasetLSICwave3K)[6:8], fitmeasures(propSDQnewdatasetLSICwave3K)[25],

fitmeasures(propSDQnewdatasetLSICwave3K)[40:43], fitmeasures(propSDQnewdatasetLSICwave3K)[50]), digits=3),

round(c(fitmeasures(tradSDQnewdatasetLSICwave4K)[6:8], fitmeasures(tradSDQnewdatasetLSICwave4K)[25],

fitmeasures(tradSDQnewdatasetLSICwave4K)[40:43], fitmeasures(tradSDQnewdatasetLSICwave4K)[50]), digits=3),

round(c(fitmeasures(threeSDQnewdatasetLSICwave4K)[6:8], fitmeasures(threeSDQnewdatasetLSICwave4K)[25],

fitmeasures(threeSDQnewdatasetLSICwave4K)[40:43], fitmeasures(threeSDQnewdatasetLSICwave4K)[50]), digits=3),

round(c(fitmeasures(propSDQnewdatasetLSICwave4K)[6:8], fitmeasures(propSDQnewdatasetLSICwave4K)[25],

fitmeasures(propSDQnewdatasetLSICwave4K)[40:43], fitmeasures(propSDQnewdatasetLSICwave4K)[50]), digits=3),

round(c(fitmeasures(tradSDQnewdatasetLSICwave6)[6:8], fitmeasures(tradSDQnewdatasetLSICwave6)[25],

fitmeasures(tradSDQnewdatasetLSICwave6)[40:43], fitmeasures(tradSDQnewdatasetLSICwave6)[50]), digits=3),

round(c(fitmeasures(threeSDQnewdatasetLSICwave6)[6:8], fitmeasures(threeSDQnewdatasetLSICwave6)[25],

fitmeasures(threeSDQnewdatasetLSICwave6)[40:43], fitmeasures(threeSDQnewdatasetLSICwave6)[50]), digits=3),

round(c(fitmeasures(propSDQnewdatasetLSICwave6)[6:8], fitmeasures(propSDQnewdatasetLSICwave6)[25],

fitmeasures(propSDQnewdatasetLSICwave6)[40:43], fitmeasures(propSDQnewdatasetLSICwave6)[50]), digits=3),

round(c(fitmeasures(tradSDQnewdatasetLSICwave8)[6:8], fitmeasures(tradSDQnewdatasetLSICwave8)[25],

fitmeasures(tradSDQnewdatasetLSICwave8)[40:43], fitmeasures(tradSDQnewdatasetLSICwave8)[50]), digits=3),

round(c(fitmeasures(threeSDQnewdatasetLSICwave8)[6:8], fitmeasures(threeSDQnewdatasetLSICwave8)[25],

fitmeasures(threeSDQnewdatasetLSICwave8)[40:43], fitmeasures(threeSDQnewdatasetLSICwave8)[50]), digits=3),

round(c(fitmeasures(propSDQnewdatasetLSICwave8)[6:8], fitmeasures(propSDQnewdatasetLSICwave8)[25],

fitmeasures(propSDQnewdatasetLSICwave8)[40:43], fitmeasures(propSDQnewdatasetLSICwave8)[50]), digits=3),

round(c(fitmeasures(tradSDQnewdatasetLSICwave10B)[6:8], fitmeasures(tradSDQnewdatasetLSICwave10B)[25],

fitmeasures(tradSDQnewdatasetLSICwave10B)[40:43], fitmeasures(tradSDQnewdatasetLSICwave10B)[50]), digits=3),

round(c(fitmeasures(threeSDQnewdatasetLSICwave10B)[6:8], fitmeasures(threeSDQnewdatasetLSICwave10B)[25],

fitmeasures(threeSDQnewdatasetLSICwave10B)[40:43], fitmeasures(threeSDQnewdatasetLSICwave10B)[50]), digits=3),

round(c(fitmeasures(propSDQnewdatasetLSICwave10B)[6:8], fitmeasures(propSDQnewdatasetLSICwave10B)[25],

fitmeasures(propSDQnewdatasetLSICwave10B)[40:43], fitmeasures(propSDQnewdatasetLSICwave10B)[50]), digits=3))

rownames(fitnewdataset) <- c("TradSDQnewdataset","ThreeSDQnewdataset","propSDQnewdataset",

"TradSDQnewdatasetLSICwave3K","ThreeSDQnewdatasetLSICwave3K","propSDQnewdatasetLSICwave3K",

"TradSDQnewdatasetLSICwave4K","ThreeSDQnewdatasetLSICwave4K","propSDQnewdatasetLSICwave4K",

"TradSDQnewdatasetLSICwave6","ThreeSDQnewdatasetLSICwave6","propSDQnewdatasetLSICwave6",

"TradSDQnewdatasetLSICwave8","ThreeSDQnewdatasetLSICwave8","propSDQnewdatasetLSICwave8",

"TradSDQnewdatasetLSICwave10B","ThreeSDQnewdatasetLSICwave10B","propSDQnewdatasetLSICwave10B")

###################

### RELIABILITY ###

###################

rbind(c(round(c((MBESS::ci.reliability(newdataset[,1:5], type="omega"))$est,

(MBESS::ci.reliability(newdataset[,1:5], type="omega"))$ci.lower,

(MBESS::ci.reliability(newdataset[,1:5], type="omega"))$ci.upper), digits=2), paste("Prosocial")),

c(round(c((MBESS::ci.reliability(newdataset[,6:10], type="omega"))$est,

(MBESS::ci.reliability(newdataset[,6:10], type="omega"))$ci.lower,

(MBESS::ci.reliability(newdataset[,6:10], type="omega"))$ci.upper), digits=2), paste("Peer Problems")),

c(round(c((MBESS::ci.reliability(newdataset[,11:15], type="omega"))$est,

(MBESS::ci.reliability(newdataset[,11:15], type="omega"))$ci.lower,

(MBESS::ci.reliability(newdataset[,11:15], type="omega"))$ci.upper), digits=2), paste("Hyperactivity")),

c(round(c((MBESS::ci.reliability(newdataset[,16:20], type="omega"))$est,

(MBESS::ci.reliability(newdataset[,16:20], type="omega"))$ci.lower,

(MBESS::ci.reliability(newdataset[,16:20], type="omega"))$ci.upper), digits=2), paste("Conduct Problems")),

c(round(c((MBESS::ci.reliability(newdataset[,21:25], type="omega"))$est,

(MBESS::ci.reliability(newdataset[,21:25], type="omega"))$ci.lower,

(MBESS::ci.reliability(newdataset[,21:25], type="omega"))$ci.upper), digits=2), paste("Emotional Problems")),

c(round(c((MBESS::ci.reliability(newdataset[,c(6:10,21:25)], type="omega"))$est,

(MBESS::ci.reliability(newdataset[,c(6:10,21:25)], type="omega"))$ci.lower,

(MBESS::ci.reliability(newdataset[,c(6:10,21:25)], type="omega"))$ci.upper), digits=2), paste("Internalizing")),

c(round(c((MBESS::ci.reliability(newdataset[,c(11:15,16:20)], type="omega"))$est,

(MBESS::ci.reliability(newdataset[,c(11:15,16:20)], type="omega"))$ci.lower,

(MBESS::ci.reliability(newdataset[,c(11:15,16:20)], type="omega"))$ci.upper), digits=2), paste("Externalizing")),

c(round(c((MBESS::ci.reliability(newdataset[,c(1:5,7:8,14:15,17)], type="omega"))$est,

(MBESS::ci.reliability(newdataset[,c(1:5,7:8,14:15,17)], type="omega"))$ci.lower,

(MBESS::ci.reliability(newdataset[,c(1:5,7:8,14:15,17)], type="omega"))$ci.upper), digits=2), paste("EGA factor 1")),

c(round(c((MBESS::ci.reliability(newdataset[,11:13], type="omega"))$est,

(MBESS::ci.reliability(newdataset[,11:13], type="omega"))$ci.lower,

(MBESS::ci.reliability(newdataset[,11:13], type="omega"))$ci.upper), digits=2), paste("EGA factor 3")),

c(round(c((MBESS::ci.reliability(newdataset[,21:25], type="omega"))$est,

(MBESS::ci.reliability(newdataset[,21:25], type="omega"))$ci.lower,

(MBESS::ci.reliability(newdataset[,21:25], type="omega"))$ci.upper), digits=2), paste("EGA factor 2")),

c(round(c((MBESS::ci.reliability(newdataset[,c(6,9:10,16,18:20)], type="omega"))$est,

(MBESS::ci.reliability(newdataset[,c(6,9:10,16,18:20)], type="omega"))$ci.lower,
