# Supplementary material for: Exploratory Graph Analysis of the Strengths and Difficulties Questionnaire for Aboriginal and/or Torres Strait Islander Children
Source: Front Psychol. 2021 Aug 18;12:573825. doi: 10.3389/fpsyg.2021.573825 (PMC8416422; doi:10.3389/fpsyg.2021.573825)
Supplement: Supplementary file 2 [file Table_2.DOCX]

Supplementary Table 1. *Item wording and item label*

| Item wording | Item label |
| --- | --- |
| Considerate of other people’s feelings | Considerate |
| Restless, overactive, cannot stay still for long | Restless |
| Often complains of headaches, stomach-aches or sickness | Somatic |
| Shares readily with other children (treats, toys, pencils etc) | Shares |
| Often loses temper | Temper |
| Rather solitary, tends to play alone | Solitary |
| Generally well behaved, usually does what adults request | Obedient |
| Many worries, often seems worried | Worries |
| Helpful if someone is hurt, upset or feeling ill | Caring |
| Constantly fidgeting or squirming | Fidgety |
| Has at least one good friend | Good friend |
| Often fights with other children or bullies them | Fights |
| Often unhappy, depressed or tearful | Unhappy |
| Generally liked by other children | Popular |
| Easily distracted, concentration wanders | Distractible |
| Nervous or clingy in new situations, easily loses confidence | Clingy |
| Kind to younger children | Kind to kids |
| Often lies or cheats | Lies |
| Picked on or bullied by other children | Bullied |
| Often volunteers to help others (parents, teachers, other children) | Helps out |
| Thinks things out before acting | Reflective |
| Steals from home, school, or elsewhere | Steals |
| Gets along better with adults than with other children | Prefer adults |
| Many fears, easily scared | Fears |
| Good attention span, see chores or homework through to the end | Persistent |

SDQ for Parents of 4-10 year olds, Australian Version. Copyright Robert Goodman, 1999, UK

Supplementary Table 2. *Item and subscale scores of caregiver-informant SDQ version 4 to 10 years*

| Item Scores | LSIC Wave 3K | LSIC Wave 4K | LSIC Wave 6 | LSIC Wave 8 | LSIC Wave 10B | SAABC Wave 5 |
| --- | --- | --- | --- | --- | --- | --- |
|  |  |  |  |  |  |  |
| considerate | 1.38 (0.56) | 1.34 (0.59) | 1.36 (0.58) | 1.38 (0.60) | 1.40 (0.58) | 1.58 (0.62) |
| shares | 1.36 (0.55) | 1.31 (0.55) | 1.33 (0.56) | 1.36 (0.56) | 1.43 (0.6) | 1.63 (0.60) |
| caring | 1.24 (0.49) | 1.17 (0.45) | 1.18 (0.46) | 1.20 (0.47) | 1.29 (0.53) | 1.43 (0.56) |
| kind | 1.22 (0.46) | 1.13 (0.37) | 1.12 (0.37) | 1.13 (0.36) | 1.20 (0.43) | 1.26 (0.47) |
| helps | 1.35 (0.59) | 1.29 (0.58) | 1.36 (0.62) | 1.37 (0.58) | 1.40 (0.58) | 1.63 (0.62) |
| solitary | 1.50 (0.68) | 1.62 (0.75) | 1.56 (0.73) | 1.60 (0.73) | 1.58 (0.68) | 1.62 (0.63) |
| friend | 1.16 (0.43) | 1.12 (0.44) | 1.10 (0.37) | 1.11 (0.38) | 1.21 (0.49) | 1.32 (0.55) |
| popular | 1.18 (0.41) | 1.10 (0.34) | 1.10 (0.35) | 1.16 (0.41) | 1.28 (0.50) | 1.28 (0.50) |
| bullied | 1.59 (0.71) | 1.65 (0.75) | 1.63 (0.77) | 1.61 (0.71) | 1.61 (0.71) | 1.42 (0.56) |
| prefer adults | 1.55 (0.70) | 1.65 (0.77) | 1.65 (0.79) | 1.63 (0.76) | 1.68 (0.71) | 1.44 (0.61) |
| restless | 2.02 (0.83) | 2.16 (0.84) | 2.04 (0.85) | 1.90 (0.83) | 1.82 (0.76) | 2.03 (0.72) |
| fidgety | 1.79 (0.81) | 1.90 (0.87) | 1.81 (0.87) | 1.75 (0.82) | 1.67 (0.76) | 1.78 (0.76) |
| distractible | 2.02 (0.77) | 2.08 (0.79) | 2.10 (0.81) | 2.07 (0.78) | 1.97 (0.77) | 1.96 (0.67) |
| reflective | 2.03 (0.74) | 1.89 (0.75) | 1.90 (0.76) | 1.88 (0.69) | 1.86 (0.64) | 2.04 (0.56) |
| persistent | 1.71 (0.75) | 1.61 (0.75) | 1.63 (0.74) | 1.70 (0.74) | 1.72 (0.70) | 1.90 (0.66) |
| tempers | 1.91 (0.75) | 2.03 (0.80) | 1.93 (0.77) | 1.82 (0.74) | 1.82 (0.70) | 1.98 (0.71) |
| obedient | 1.47 (0.59) | 1.40 (0.59) | 1.39 (0.59) | 1.36 (0.55) | 1.46 (0.59) | 1.68 (0.57) |
| fights | 1.36 (0.60) | 1.43 (0.68) | 1.34 (0.62) | 1.30 (0.56) | 1.28 (0.53) | 1.34 (0.57) |
| lies | 1.47 (0.61) | 1.61 (0.73) | 1.53 (0.68) | 1.49 (0.65) | 1.42 (0.60) | 1.35 (0.52) |
| steals | 1.12 (0.40) | 1.14 (0.45) | 1.13 (0.40) | 1.14 (0.43) | 1.15 (0.44) | 1.10 (0.33) |
| somatic | 1.46 (0.64) | 1.48 (0.69) | 1.43 (0.65) | 1.48 (0.67) | 1.51 (0.66) | 1.37 (0.58) |
| worries | 1.25 (0.53) | 1.30 (0.55) | 1.35 (0.61) | 1.42 (0.63) | 1.47 (0.63) | 1.35 (0.56) |
| unhappy | 1.24 (0.51) | 1.30 (0.55) | 1.28 (0.54) | 1.27 (0.53) | 1.32 (0.55) | 1.24 (0.47) |
| clingy | 1.75 (0.80) | 1.81 (0.83) | 1.76 (0.83) | 1.73 (0.79) | 1.67 (0.73) | 1.77 (0.72) |
| fears | 1.64 (0.74) | 1.76 (0.82) | 1.67 (0.78) | 1.63 (0.76) | 1.54 (0.69) | 1.70 (0.64) |
| Subscale Scores |  |  |  |  |  |  |
|  |  |  |  |  |  |  |
| Prosocial Behavior | 6.55 (1.74) | 6.24 (1.65) | 6.35 (1.65) | 6.45 (1.64) | 6.72 (1.89) | 7.53 (2.03) |
| Peer Problems | 6.99 (1.63) | 7.14 (1.69) | 7.04 (1.74) | 7.11 (1.71) | 7.35 (1.77) | 7.08 (1.82) |
| Hyperactivity | 9.57 (2.47) | 9.63 (2.62) | 9.48 (2.74) | 9.30 (2.62) | 9.04 (2.53) | 9.71 (2.62) |
| Conduct Problems | 7.34 (1.89) | 7.61 (1.94) | 7.33 (2.03) | 7.11 (1.90) | 7.13 (1.88) | 7.45 (1.80) |
| Emotional Problems | 7.34 (2.00) | 7.66 (2.09) | 7.49 (2.15) | 7.52 (2.16) | 7.51 (2.21) | 7.43 (1.98) |

Note. Mean and standard deviations are reported (Mean (SD)).

Supplementary Table 3. *Number of SDQ dimensions identified by Exploratory Graph Analysis with TMFG estimation*

|  | LSIC | | | | | | | SAABC |  |  |  |  |
| --- | --- | --- | --- | --- | --- | --- | --- | --- | --- | --- | --- | --- |
|  | Wave 3  Cohort K | | Wave 4  Cohort K | Wave 6  Cohorts B & K | Wave 8  Cohorts B & K | Wave 10  Cohort B | | Wave 5 |  |  |  |  |
|  |  |  | | | | |  | | |  |  | Wave 5 |
| Number of dimensions | 3 | | 5 | 3 | 3 | 4 | | 4 |  |  |  |  |
|  |  |  |  |  |  |  |  |  |  |  |  |  |
| Number of dimensions | Percentages (%) | | Percentages (%) | Percentages (%) | Percentages (%) | Percentages (%) | | Percentages (%) |  |  |  |  |
| 2 | 0.00 | | 0.00 | 0.00 | 0.00 | 0.00 | | 0.01 |  |  |  |  |
| 3 | 0.97 | | 0.72 | 0.73 | 0.90 | 0.30 | | 0.53 |  |  |  |  |
| 4 | 0.03 | | 0.27 | 0.26 | 0.09 | 0.70 | | 0.44 |  |  |  |  |
| 5 | 0.00 | | 0.01 | 0.01 | 0.01 | 0.00 | | 0.02 |  |  |  |  |

*Note*. The “Study samples” row indicates the number of dimensions identified by EGA. The “Bootstrap samples” rows indicate the percentage of times that each number of dimensions were identified over the 2500 bootstrap samples.

Supplementary Table 4. *Network loadings of SDQ items in LSIC Wave 3K*

| Item | Dimension 1 | Dimension 2 | Dimension 3 |
| --- | --- | --- | --- |
| considerate | 0.00 | **0.17** | 0.07 |
| shares | 0.03 | **0.22** | 0.08 |
| caring | 0.03 | **0.30** | 0.01 |
| kind | 0.04 | **0.29** | -0.05 |
| helps | 0.07 | **0.23** | 0.00 |
| solitary | 0.10 | 0.05 | 0.03 |
| friend | 0.02 | **0.34** | -0.05 |
| popular | 0.03 | **0.31** | -0.03 |
| bullied | 0.13 | 0.02 | **0.15** |
| prefer adults | 0.08 | 0.06 | **0.15** |
| restless | 0.03 | 0.05 | **0.24** |
| fidgety | 0.06 | -0.03 | **0.32** |
| distractible | 0.12 | 0.09 | **0.18** |
| reflective | 0.00 | **0.19** | 0.08 |
| persistent | 0.00 | **0.21** | 0.06 |
| tempers | 0.06 | 0.05 | 0.14 |
| obedient | 0.04 | **0.21** | 0.14 |
| fights | 0.12 | 0.05 | **0.24** |
| lies | 0.02 | 0.10 | **0.24** |
| steals | 0.00 | 0.07 | **0.27** |
| somatic | 0.14 | 0.04 | 0.05 |
| worries | **0.33** | -0.01 | 0.00 |
| unhappy | **0.40** | 0.06 | 0.04 |
| clingy | **0.20** | 0.00 | 0.11 |
| fears | **0.27** | -0.01 | 0.03 |

Note. The dimensions were identified by EGA. Network loadings higher than 0.15 (moderate loadings) were highlighted in bold.

Supplementary Table 5. *Network loadings of SDQ items in LSIC Wave 4K*

| Item | Dimension 1 | Dimension 2 | Dimension 3 | Dimension 4 |
| --- | --- | --- | --- | --- |
| considerate | 0.04 | **0.28** | 0.09 | 0.05 |
| shares | 0.03 | **0.17** | 0.10 | 0.00 |
| caring | 0.04 | **0.29** | 0.08 | 0.00 |
| kind | 0.02 | **0.33** | 0.14 | -0.01 |
| helps | 0.03 | **0.20** | 0.05 | 0.02 |
| solitary | 0.02 | 0.05 | 0.15 | -0.01 |
| friend | 0.04 | **0.26** | 0.05 | 0.00 |
| popular | 0.08 | **0.19** | 0.06 | -0.09 |
| bullied | **0.20** | -0.01 | 0.11 | 0.02 |
| prefer adults | 0.08 | 0.06 | 0.02 | 0.06 |
| restless | 0.04 | 0.03 | -0.02 | **0.34** |
| fidgety | 0.14 | 0.06 | 0.11 | **0.40** |
| distractible | 0.02 | 0.06 | 0.08 | **0.21** |
| reflective | 0.01 | **0.17** | -0.02 | 0.02 |
| persistent | 0.02 | **0.22** | 0.06 | 0.12 |
| tempers | 0.15 | 0.08 | 0.04 | 0.01 |
| obedient | 0.09 | **0.27** | 0.03 | 0.03 |
| fights | **0.20** | 0.04 | 0.02 | 0.07 |
| lies | **0.24** | 0.01 | 0.00 | 0.00 |
| steals | **0.27** | 0.04 | 0.00 | 0.10 |
| somatic | **0.16** | -0.04 | 0.02 | 0.02 |
| worries | **0.22** | 0.03 | 0.05 | 0.00 |
| unhappy | **0.32** | 0.04 | 0.08 | 0.03 |
| clingy | **0.13** | 0.05 | 0.08 | 0.06 |
| fears | **0.18** | -0.01 | 0.21 | 0.05 |

Note. The dimensions were identified by EGA. Network loadings higher than higher than 0.15 (moderate loadings) were highlighted in bold.

Supplementary Table 6. *Network loadings of SDQ items in LSIC Wave 6*

| Item | Dimension 1 | Dimension 2 | Dimension 3 | Dimension 4 | Dimension 5 |
| --- | --- | --- | --- | --- | --- |
| considerate | -0.01 | **0.21** | 0.00 | 0.10 | 0.11 |
| shares | 0.09 | **0.19** | 0.00 | 0.08 | 0.14 |
| caring | 0.00 | **0.29** | 0.00 | 0.00 | 0.01 |
| kind | -0.03 | **0.38** | 0.00 | 0.04 | 0.05 |
| helps | -0.03 | 0.15 | 0.00 | 0.00 | 0.10 |
| solitary | 0.13 | 0.08 | 0.00 | 0.00 | 0.00 |
| friend | 0.05 | **0.21** | 0.00 | -0.08 | 0.06 |
| popular | 0.12 | **0.27** | 0.00 | 0.02 | 0.07 |
| bullied | 0.12 | -0.06 | 0.04 | 0.10 | 0.00 |
| prefer adults | 0.15 | 0.04 | 0.06 | 0.01 | -0.05 |
| restless | 0.08 | 0.00 | **0.34** | 0.05 | 0.08 |
| fidgety | 0.10 | 0.00 | **0.35** | 0.09 | 0.02 |
| distractible | 0.06 | 0.00 | 0.14 | 0.07 | **0.24** |
| reflective | -0.02 | 0.12 | 0.07 | 0.01 | **0.16** |
| persistent | 0.03 | 0.08 | **0.19** | 0.00 | **0.17** |
| tempers | 0.08 | 0.08 | 0.09 | 0.14 | 0.09 |
| obedient | 0.00 | 0.14 | 0.07 | 0.15 | 0.14 |
| fights | 0.10 | 0.03 | 0.06 | **0.29** | 0.10 |
| lies | 0.05 | 0.08 | 0.00 | **0.19** | 0.00 |
| steals | 0.03 | 0.04 | 0.09 | **0.30** | 0.00 |
| somatic | **0.20** | -0.03 | 0.06 | 0.02 | -0.02 |
| worries | **0.22** | 0.02 | 0.01 | 0.07 | 0.01 |
| unhappy | **0.30** | 0.05 | 0.10 | 0.08 | 0.00 |
| clingy | **0.23** | -0.04 | 0.07 | 0.02 | 0.00 |
| fears | **0.25** | 0.00 | 0.01 | 0.04 | 0.00 |

Note. The dimensions were identified by EGA. Network loadings higher than 0.15 (moderate loadings) were highlighted in bold.

Supplementary Table 7. *Network loadings of SDQ items in LSIC Wave 8*

| Item | Dimension 1 | Dimension 2 | Dimension 3 |
| --- | --- | --- | --- |
| considerate | 0.06 | **0.18** | 0.08 |
| shares | 0.02 | **0.18** | 0.09 |
| caring | -0.02 | **0.32** | -0.01 |
| kind | 0.02 | **0.30** | 0.05 |
| helps | -0.07 | **0.25** | 0.02 |
| solitary | 0.06 | 0.09 | 0.00 |
| friend | -0.03 | **0.30** | 0.00 |
| popular | 0.04 | **0.28** | 0.02 |
| bullied | **0.16** | 0.06 | 0.11 |
| prefer adults | 0.12 | -0.10 | 0.02 |
| restless | **0.26** | 0.07 | 0.03 |
| fidgety | **0.32** | 0.02 | 0.03 |
| distractible | **0.17** | 0.07 | 0.12 |
| reflective | -0.06 | **0.19** | 0.00 |
| persistent | 0.10 | **0.18** | 0.00 |
| tempers | 0.15 | 0.06 | **0.16** |
| obedient | 0.05 | **0.21** | 0.06 |
| fights | **0.17** | 0.09 | 0.22 |
| lies | 0.05 | 0.06 | **0.25** |
| steals | 0.09 | 0.03 | **0.32** |
| somatic | **0.20** | -0.04 | -0.01 |
| worries | **0.23** | 0.04 | 0.00 |
| unhappy | **0.30** | 0.04 | 0.06 |
| clingy | **0.21** | 0.01 | 0.00 |
| fears | **0.22** | -0.02 | 0.03 |

Note. The dimensions were identified by EGA. Network loadings higher than 0.15 (moderate loadings) were highlighted in bold.

Supplementary Table 8. *Network loadings of SDQ items in LSIC Wave 10B*

| Item | Dimension 1 | Dimension 2 | Dimension 3 | Dimension 4 |
| --- | --- | --- | --- | --- |
| considerate | **0.24** | 0.06 | 0.00 | 0.00 |
| shares | **0.28** | 0.00 | 0.02 | 0.00 |
| caring | **0.29** | 0.04 | 0.00 | 0.00 |
| kind | **0.28** | 0.02 | 0.00 | 0.00 |
| helps | **0.28** | -0.04 | -0.03 | 0.00 |
| solitary | 0.04 | 0.05 | 0.12 | 0.00 |
| friend | **0.23** | 0.07 | 0.00 | 0.00 |
| popular | **0.30** | 0.09 | 0.05 | 0.00 |
| bullied | 0.04 | **0.18** | 0.08 | 0.05 |
| prefer adults | -0.10 | **0.16** | 0.05 | 0.01 |
| restless | 0.02 | 0.01 | 0.12 | **0.36** |
| fidgety | 0.02 | 0.05 | 0.10 | **0.38** |
| distractible | 0.06 | 0.09 | 0.06 | **0.24** |
| reflective | **0.23** | -0.06 | -0.01 | 0.03 |
| persistent | **0.22** | 0.00 | 0.02 | 0.14 |
| tempers | 0.03 | **0.16** | 0.10 | 0.11 |
| obedient | **0.25** | -0.05 | 0.04 | 0.00 |
| fights | 0.08 | 0.15 | **0.20** | 0.05 |
| lies | -0.05 | **0.34** | 0.04 | 0.01 |
| steals | 0.01 | **0.29** | 0.11 | 0.08 |
| somatic | -0.02 | 0.04 | **0.18** | 0.06 |
| worries | 0.02 | 0.04 | **0.29** | 0.03 |
| unhappy | 0.04 | 0.18 | **0.20** | 0.07 |
| clingy | 0.00 | 0.08 | **0.23** | 0.09 |
| fears | -0.01 | 0.09 | **0.28** | 0.02 |

Note. The dimensions were identified by EGA. Network loadings higher than 0.15 (moderate loadings) were highlighted in bold.

Supplementary Table 9. *Network loadings of SDQ items in SAABC*

| Item | Dimension 1 | Dimension 2 | Dimension 3 |
| --- | --- | --- | --- |
| considerate | 0.00 | 0.00 | **0.28** |
| shares | 0.04 | 0.03 | **0.22** |
| caring | 0.03 | 0.00 | **0.41** |
| kind | 0.05 | 0.00 | **0.29** |
| helps | 0.03 | 0.09 | 0.14 |
| solitary | 0.14 | 0.07 | 0.01 |
| friend | 0.06 | 0.01 | **0.25** |
| popular | 0.03 | 0.00 | **0.31** |
| bullied | **0.20** | 0.02 | 0.01 |
| prefer adults | **0.24** | 0.00 | 0.06 |
| restless | 0.04 | **0.29** | 0.02 |
| fidgety | 0.11 | **0.35** | 0.00 |
| distractible | 0.12 | **0.36** | 0.01 |
| reflective | 0.08 | **0.19** | 0.10 |
| persistent | 0.00 | **0.22** | **0.16** |
| tempers | **0.17** | 0.15 | 0.03 |
| obedient | 0.04 | **0.21** | **0.20** |
| fights | **0.25** | 0.04 | 0.10 |
| lies | **0.24** | 0.00 | 0.06 |
| steals | **0.23** | 0.01 | 0.04 |
| somatic | **0.22** | 0.10 | 0.00 |
| worries | **0.23** | 0.02 | 0.03 |
| unhappy | **0.30** | 0.02 | 0.01 |
| clingy | 0.15 | 0.07 | 0.00 |
| fears | **0.25** | 0.02 | -0.01 |

Note. The dimensions were identified by EGA. Network loadings higher than 0.15 (moderate loadings) were highlighted in bold.

Supplementary Table 10. *Model fit comparison of the original and proposed structure of the SDQ*

| **Model** | **χ^2^** | ***df*** | ***p-*value** | **RMSEA** | **90% CI** | **CFI** | **SRMR** |
| --- | --- | --- | --- | --- | --- | --- | --- |
| **LSIC Wave 3K** |  |  |  |  |  |  |  |
| ***Factor structures*** |  |  |  |  |  |  |  |
| *5-dimensional structure* | 981.2 | 265 | <0.001 | 0.077 | [0.067, 0.077] | 0.780 | 0.114 |
| *3 -dimensional structure* | 1161.9 | 272 | <0.001 | 0.070 | [0.075, 0.084] | 0.726 | 0.126 |
| *4-dimensional structure (proposed)* | 604.1 | 269 | <0.001 | 0.049 | [0.044, 0.054] | 0.897 | 0.091 |
|  |  |  |  |  |  |  |  |
| **LSIC Wave 4K** |  |  |  |  |  |  |  |
| ***Factor structures*** |  |  |  |  |  |  |  |
| *5-dimensional structure* | 603.6 | 265 | <0.001 | 0.051 | [0.046, 0.057] | 0.849 | 0.093 |
| *3 -dimensional structure* | 715.5 | 272 | <0.001 | 0.058 | [0.053, 0.063] | 0.803 | 0.105 |
| *4-dimensional structure (proposed)* | 483.9 | 269 | <0.001 | 0.040 | [0.035, 0.046] | 0.904 | 0.090 |
|  |  |  |  |  |  |  |  |
| **LSIC Wave 6** |  |  |  |  |  |  |  |
| ***Factor structures*** |  |  |  |  |  |  |  |
| *5-dimensional structure* | 1044.7 | 265 | <0.001 | 0.052 | [0.049, 0.056] | 0.890 | 0.077 |
| *3 -dimensional structure* | 1301.1 | 272 | <0.001 | 0.059 | [0.056, 0.063] | 0.855 | 0.091 |
| *4-dimensional structure (proposed)* | 930.6 | 269 | <0.001 | 0.048 | [0.045, 0.051] | 0.907 | 0.080 |
|  |  |  |  |  |  |  |  |
| **LSIC Wave 8** |  |  |  |  |  |  |  |
| ***Factor structures*** |  |  |  |  |  |  |  |
| *5-dimensional structure* | 1255.3 | 265 | <0.001 | 0.062 | [0.058, 0.065] | 0.855 | 0.089 |
| *3 -dimensional structure* | 1616.1 | 272 | <0.001 | 0.071 | [0.067, 0.074] | 0.803 | 0.103 |
| *4-dimensional structure (proposed)* | 977.6 | 269 | <0.001 | 0.052 | [0.048, 0.055] | 0.896 | 0.084 |
|  |  |  |  |  |  |  |  |
| **LSIC Wave 10B** |  |  |  |  |  |  |  |
| ***Factor structures*** |  |  |  |  |  |  |  |
| *5-dimensional structure* | 1614.2 | 265 | <0.001 | 0.084 | [0.080, 0.088] | 0.811 | 0.116 |
| *3 -dimensional structure* | 1914.7 | 272 | <0.001 | 0.091 | [0.087, 0.095] | 0.769 | 0.127 |
| *4-dimensional structure (proposed)* | 916.0 | 269 | <0.001 | 0.058 | [0.054, 0.062] | 0.909 | 0.084 |
|  |  |  |  |  |  |  |  |
| **SAABC Wave 5** |  |  |  |  |  |  |  |
| ***Factor structures*** |  |  |  |  |  |  |  |
| *5-dimensional structure* | 543.4 | 265 | <0.001 | 0.065 | [0.057, 0.073] | 0.918 | 0.099 |
| *3 -dimensional structure* | 641.8 | 272 | <0.001 | 0.074 | [0.067, 0.081] | 0.891 | 0.110 |
| *4-dimensional structure (proposed)* | 546.5 | 269 | <0.001 | 0.064 | [0.057, 0.072] | 0.918 | 0.099 |

*Note*. The original 5-dimensional structure was suggested by R. Goodman, Meltzer, and Bailey (1998). The 3-dimensional structure was suggested by A. Goodman, Lamping, and Ploubidis (2010).(Kan, de Jonge, van der Maas, Levine, & Epskamp, 2020)

Supplementary Table 11. *Restricted factor model of five-factor SDQ structure in the SAABC*

| **Factor Loadings** |  |  |  |  |  |
| --- | --- | --- | --- | --- | --- |
|  | *Prosocial behaviour* | *Peer Problems* | *Hyperactivity* | *Conduct Problems* | *Emotional Problems* |
| considerate | **0.74 (0.03)** | 0.00 (0.00) | 0.00 (0.00) | 0.00 (0.00) | 0.00 (0.00) |
| shares | **0.68 (0.04)** | 0.00 (0.00) | 0.00 (0.00) | 0.00 (0.00) | 0.00 (0.00) |
| caring | **0.79 (0.03)** | 0.00 (0.00) | 0.00 (0.00) | 0.00 (0.00) | 0.00 (0.00) |
| kind | **0.66 (0.04)** | 0.00 (0.00) | 0.00 (0.00) | 0.00 (0.00) | 0.00 (0.00) |
| helps | **0.62 (0.04)** | 0.00 (0.00) | 0.00 (0.00) | 0.00 (0.00) | 0.00 (0.00) |
| solitary | 0.00 (0.00) | **0.44 (0.06)** | 0.00 (0.00) | 0.00 (0.00) | 0.00 (0.00) |
| friend | 0.00 (0.00) | **0.82 (0.03)** | 0.00 (0.00) | 0.00 (0.00) | 0.00 (0.00) |
| popular | 0.00 (0.00) | **0.77 (0.03)** | 0.00 (0.00) | 0.00 (0.00) | 0.00 (0.00) |
| bullied | 0.00 (0.00) | **0.40 (0.06)** | 0.00 (0.00) | 0.00 (0.00) | 0.00 (0.00) |
| prefer adults | 0.00 (0.00) | **0.52 (0.05)** | 0.00 (0.00) | 0.00 (0.00) | 0.00 (0.00) |
| restless | 0.00 (0.00) | 0.00 (0.00) | **0.80 (0.03)** | 0.00 (0.00) | 0.00 (0.00) |
| fidgety | 0.00 (0.00) | 0.00 (0.00) | **0.79 (0.03)** | 0.00 (0.00) | 0.00 (0.00) |
| distractible | 0.00 (0.00) | 0.00 (0.00) | **0.84 (0.02)** | 0.00 (0.00) | 0.00 (0.00) |
| reflective | 0.00 (0.00) | 0.00 (0.00) | **0.65 (0.04)** | 0.00 (0.00) | 0.00 (0.00) |
| persistent | 0.00 (0.00) | 0.00 (0.00) | **0.73 (0.03)** | 0.00 (0.00) | 0.00 (0.00) |
| tempers | 0.00 (0.00) | 0.00 (0.00) | 0.00 (0.00) | **0.74 (0.03)** | 0.00 (0.00) |
| obedient | 0.00 (0.00) | 0.00 (0.00) | 0.00 (0.00) | **0.72 (0.04)** | 0.00 (0.00) |
| fights | 0.00 (0.00) | 0.00 (0.00) | 0.00 (0.00) | **0.69 (0.04)** | 0.00 (0.00) |
| lies | 0.00 (0.00) | 0.00 (0.00) | 0.00 (0.00) | **0.57 (0.05)** | 0.00 (0.00) |
| steals | 0.00 (0.00) | 0.00 (0.00) | 0.00 (0.00) | **0.49 (0.05)** | 0.00 (0.00) |
| somatic | 0.00 (0.00) | 0.00 (0.00) | 0.00 (0.00) | 0.00 (0.00) | **0.62 (0.05)** |
| worries | 0.00 (0.00) | 0.00 (0.00) | 0.00 (0.00) | 0.00 (0.00) | **0.75 (0.04)** |
| unhappy | 0.00 (0.00) | 0.00 (0.00) | 0.00 (0.00) | 0.00 (0.00) | **0.78 (0.03)** |
| clingy | 0.00 (0.00) | 0.00 (0.00) | 0.00 (0.00) | 0.00 (0.00) | **0.47 (0.06)** |
| fears | 0.00 (0.00) | 0.00 (0.00) | 0.00 (0.00) | 0.00 (0.00) | **0.59 (0.05)** |
|  |  |  |  |  |  |
| **Factor Correlations** |  |  |  |  |  |
| Prosocial behaviour x Peer Problems | 0.76 (0.04) |  |  |  |  |
| Prosocial behaviour x Hyperactivity | 0.49 (0.06) |  |  |  |  |
| Prosocial behaviour x Conduct Problems | 0.73 (0.05) |  |  |  |  |
| Prosocial behaviour x Emotional Problems | 0.33 (0.07) |  |  |  |  |
| Peer Problems x Hyperactivity | 0.44 (0.06) |  |  |  |  |
| Peer Problems x Conduct Problems | 0.57 (0.06) |  |  |  |  |
| Peer Problems x Emotional Problems | 0.58 (0.06) |  |  |  |  |
| Hyperactivity x Conduct Problems | 0.81 (0.04) |  |  |  |  |
| Hyperactivity x Emotional Problems | 0.59 (0.05) |  |  |  |  |
| Conduct Problems x Emotional Problems | 0.71 (0.05) |  |  |  |  |

Note. Table reports estimates and standard errors (Estimates (SE)). The factor loadings on the theoretical SDQ factors are highlighted in bold.

Supplementary Table 12. *Restricted factor model of three-factor SDQ structure in the SAABC*

| **Factor Loadings** |  |  |  |
| --- | --- | --- | --- |
|  | *Prosocial behaviour* | *Internalizing* | *Externalizing* |
| considerate | **0.73 (0.04)** | 0.00 (0.00) | 0.00 (0.00) |
| shares | **0.67 (0.04)** | 0.00 (0.00) | 0.00 (0.00) |
| caring | **0.79 (0.03)** | 0.00 (0.00) | 0.00 (0.00) |
| kind | **0.66 (0.04)** | 0.00 (0.00) | 0.00 (0.00) |
| helps | **0.64 (0.04)** | 0.00 (0.00) | 0.00 (0.00) |
| solitary | 0.00 (0.00) | **0.57 (0.05)** | 0.00 (0.00) |
| friend | 0.00 (0.00) | **0.51 (0.05)** | 0.00 (0.00) |
| popular | 0.00 (0.00) | **0.48 (0.05)** | 0.00 (0.00) |
| bullied | 0.00 (0.00) | **0.56 (0.05)** | 0.00 (0.00) |
| prefer adults | 0.00 (0.00) | **0.65 (0.04)** | 0.00 (0.00) |
| somatic | 0.00 (0.00) | **0.62 (0.04)** | 0.00 (0.00) |
| worries | 0.00 (0.00) | **0.69 (0.04)** | 0.00 (0.00) |
| unhappy | 0.00 (0.00) | **0.71 (0.04)** | 0.00 (0.00) |
| clingy | 0.00 (0.00) | **0.48 (0.05)** | 0.00 (0.00) |
| fears | 0.00 (0.00) | **0.54 (0.05)** | 0.00 (0.00) |
| restless | 0.00 (0.00) | 0.00 (0.00) | **0.75 (0.03)** |
| fidgety | 0.00 (0.00) | 0.00 (0.00) | **0.74 (0.03)** |
| distractible | 0.00 (0.00) | 0.00 (0.00) | **0.79 (0.03)** |
| reflective | 0.00 (0.00) | 0.00 (0.00) | **0.65 (0.04)** |
| persistent | 0.00 (0.00) | 0.00 (0.00) | **0.75 (0.03)** |
| tempers | 0.00 (0.00) | 0.00 (0.00) | **0.72 (0.03)** |
| obedient | 0.00 (0.00) | 0.00 (0.00) | **0.70 (0.04)** |
| fights | 0.00 (0.00) | 0.00 (0.00) | **0.61 (0.04)** |
| lies | 0.00 (0.00) | 0.00 (0.00) | **0.50 (0.05)** |
| steals | 0.00 (0.00) | 0.00 (0.00) | **0.45 (0.05)** |
| **Factor Correlations** |  |  |  |
| Prosocial behaviour x Internalizing | 0.47 (0.06) |  |  |
| Prosocial behaviour x Externalizing | 0.62 (0.05) |  |  |
| Internalizing x Externalizing | 0.70 (0.04) |  |  |

Note. Table reports estimates and standard errors (Estimates (SE)). The factor loadings on the theoretical SDQ factors are highlighted in bold.

Supplementary Table 13. *Unrestricted factor model of three-factor SDQ structure in the SAABC*

| **Factor Loadings** |  |  |  |
| --- | --- | --- | --- |
|  | *Factor 1* | *Factor 2* | *Factor 3* |
| considerate | **0.72 (0.04)** | 0.09 (0.07) | -0.05 (0.07) |
| shares | **0.58 (0.05)** | 0.20 (0.07) | 0.03 (0.07) |
| caring | **0.75 (0.03)** | 0.04 (0.00) | 0.06 (0.00) |
| kind | **0.64 (0.05)** | 0.06 (0.07) | 0.00 (0.07) |
| helps | **0.49 (0.06)** | 0.29 (0.07) | -0.06 (0.07) |
| solitary | 0.05 (0.07) | 0.24 (0.07) | **0.41 (0.06)** |
| friend | **0.69 (0.05)** | -0.21 (0.07) | 0.34 (0.07) |
| popular | **0.70 (0.05)** | -0.13 (0.07) | 0.27 (0.07) |
| bullied | 0.04 (0.07) | 0.16 (0.07) | **0.49 (0.06)** |
| adults | 0.10 (0.07) | 0.01 (0.08) | **0.63 (0.05)** |
| restless | 0.03 (0.06) | **0.69 (0.05)** | 0.13 (0.06) |
| fidgety | -0.09 (0.06) | **0.69 (0.05)** | 0.25 (0.06) |
| distractible | -0.08 (0.00) | **0.80 (0.03)** | 0.14 (0.01) |
| reflective | 0.18 (0.07) | **0.76 (0.05)** | -0.24 (0.07) |
| persistent | 0.34 (0.06) | **0.61 (0.05)** | 0.00 (0.06) |
| tempers | 0.06 (0.06) | **0.58 (0.05)** | 0.24 (0.06) |
| obedient | **0.55 (0.05)** | **0.47 (0.06)** | -0.04 (0.06) |
| fights | 0.18 (0.06) | 0.36 (0.06) | 0.30 (0.06) |
| lies | 0.20 (0.07) | 0.13 (0.07) | **0.43 (0.06)** |
| steals | 0.12 (0.07) | 0.13 (0.07) | **0.40 (0.06)** |
| somatic | 0.04 (0.00) | -0.05 (0.00) | **0.72 (0.04)** |
| worries | 0.02 (0.07) | 0.13 (0.07) | **0.60 (0.05)** |
| unhappy | 0.06 (0.07) | 0.23 (0.07) | **0.58 (0.05)** |
| clingy | -0.10 (0.07) | 0.23 (0.07) | **0.38 (0.07)** |
| fears | -0.05 (0.07) | 0.17 (0.08) | **0.44 (0.06)** |
| **Factor Correlations** |  |  |  |
| Factor 1 x Factor 2 | 0.32 (0.09) |  |  |
| Factor 1 x Factor 3 | 0.20 (0.11) |  |  |
| Factor 2 x Factor 3 | 0.37 (0.1) |  |  |

Note. Table reports estimates and standard errors (Estimates (SE)). Substantive factor loadings (>.40) are highlighted in bold.

Supplementary Table 14. *Restricted factor model of the proposed four-factor SDQ structure in the SAABC*

| **Factor Loadings** |  |  |  |  |
| --- | --- | --- | --- | --- |
|  | *Factor 1* | *Factor 2* | *Factor 3* | *Factor 4* |
| considerate | **0.70 (0.04)** | 0.00 (0.00) | 0.00 (0.00) | 0.00 (0.00) |
| shares | **0.68 (0.04)** | 0.00 (0.00) | 0.00 (0.00) | 0.00 (0.00) |
| caring | **0.74 (0.03)** | 0.00 (0.00) | 0.00 (0.00) | 0.00 (0.00) |
| kind | **0.64 (0.04)** | 0.00 (0.00) | 0.00 (0.00) | 0.00 (0.00) |
| helps | **0.61 (0.04)** | 0.00 (0.00) | 0.00 (0.00) | 0.00 (0.00) |
| obedient | **0.79 (0.03)** | 0.00 (0.00) | 0.00 (0.00) | 0.00 (0.00) |
| friend | **0.62 (0.04)** | 0.00 (0.00) | 0.00 (0.00) | 0.00 (0.00) |
| popular | **0.66 (0.04)** | 0.00 (0.00) | 0.00 (0.00) | 0.00 (0.00) |
| reflective | **0.56 (0.05)** | 0.00 (0.00) | 0.00 (0.00) | 0.00 (0.00) |
| persistent | **0.70 (0.04)** | 0.00 (0.00) | 0.00 (0.00) | 0.00 (0.00) |
| solitary | 0.00 (0.00) | **0.53 (0.05)** | 0.00 (0.00) | 0.00 (0.00) |
| bullied | 0.00 (0.00) | **0.58 (0.05)** | 0.00 (0.00) | 0.00 (0.00) |
| adults | 0.00 (0.00) | **0.57 (0.05)** | 0.00 (0.00) | 0.00 (0.00) |
| tempers | 0.00 (0.00) | **0.73 (0.04)** | 0.00 (0.00) | 0.00 (0.00) |
| fights | 0.00 (0.00) | **0.71 (0.04)** | 0.00 (0.00) | 0.00 (0.00) |
| steals | 0.00 (0.00) | **0.57 (0.05)** | 0.00 (0.00) | 0.00 (0.00) |
| lies | 0.00 (0.00) | **0.59 (0.05)** | 0.00 (0.00) | 0.00 (0.00) |
| unhappy | 0.00 (0.00) | 0.00 (0.00) | **0.80 (0.03)** | 0.00 (0.00) |
| clingy | 0.00 (0.00) | 0.00 (0.00) | **0.47 (0.06)** | 0.00 (0.00) |
| fears | 0.00 (0.00) | 0.00 (0.00) | **0.57 (0.05)** | 0.00 (0.00) |
| worries | 0.00 (0.00) | 0.00 (0.00) | **0.71 (0.04)** | 0.00 (0.00) |
| somatic | 0.00 (0.00) | 0.00 (0.00) | **0.64 (0.04)** | 0.00 (0.00) |
| restless | 0.00 (0.00) | 0.00 (0.00) | 0.00 (0.00) | **0.83 (0.03)** |
| fidgety | 0.00 (0.00) | 0.00 (0.00) | 0.00 (0.00) | **0.84 (0.03)** |
| distractible | 0.00 (0.00) | 0.00 (0.00) | 0.00 (0.00) | **0.82 (0.03)** |
|  |  |  |  |  |
| **Factor Correlations** |  |  |  |  |
| Factor 1 x Factor 2 | 0.63 (0.05) |  |  |  |
| Factor 1 x Factor 3 | 0.44 (0.06) |  |  |  |
| Factor 1 x Factor 4 | 0.55 (0.05) |  |  |  |
| Factor 2 x Factor 3 | 0.81 (0.04) |  |  |  |
| Factor 2 x Factor 4 | 0.74 (0.04) |  |  |  |
| Factor 3 x Factor 4 | 0.61 (0.05) |  |  |  |

Note. Table reports estimates and standard errors (Estimates (SE)). The factor loadings on the theoretical SDQ factors are highlighted in bold.

Supplementary Table 15. *Restricted factor model of five-factor SDQ structure in LSIC Wave 3K*

| **Factor Loadings** |  |  |  |  |  |
| --- | --- | --- | --- | --- | --- |
|  | *Prosocial behaviour* | *Peer Problems* | *Hyperactivity* | *Conduct Problems* | *Emotional Problems* |
| considerate | **0.57 (0.03)** | 0.00 (0.00) | 0.00 (0.00) | 0.00 (0.00) | 0.00 (0.00) |
| shares | **0.67 (0.03)** | 0.00 (0.00) | 0.00 (0.00) | 0.00 (0.00) | 0.00 (0.00) |
| caring | **0.73 (0.03)** | 0.00 (0.00) | 0.00 (0.00) | 0.00 (0.00) | 0.00 (0.00) |
| kind | **0.73 (0.03)** | 0.00 (0.00) | 0.00 (0.00) | 0.00 (0.00) | 0.00 (0.00) |
| helps | **0.63 (0.03)** | 0.00 (0.00) | 0.00 (0.00) | 0.00 (0.00) | 0.00 (0.00) |
| solitary | 0.00 (0.00) | **0.15 (0.05)** | 0.00 (0.00) | 0.00 (0.00) | 0.00 (0.00) |
| friend | 0.00 (0.00) | **0.82 (0.02)** | 0.00 (0.00) | 0.00 (0.00) | 0.00 (0.00) |
| popular | 0.00 (0.00) | **0.90 (0.02)** | 0.00 (0.00) | 0.00 (0.00) | 0.00 (0.00) |
| bullied | 0.00 (0.00) | **0.17 (0.05)** | 0.00 (0.00) | 0.00 (0.00) | 0.00 (0.00) |
| prefer adults | 0.00 (0.00) | **0.04 (0.05)** | 0.00 (0.00) | 0.00 (0.00) | 0.00 (0.00) |
| restless | 0.00 (0.00) | 0.00 (0.00) | **0.77 (0.03)** | 0.00 (0.00) | 0.00 (0.00) |
| fidgety | 0.00 (0.00) | 0.00 (0.00) | **0.77 (0.03)** | 0.00 (0.00) | 0.00 (0.00) |
| distractible | 0.00 (0.00) | 0.00 (0.00) | **0.61 (0.03)** | 0.00 (0.00) | 0.00 (0.00) |
| reflective | 0.00 (0.00) | 0.00 (0.00) | **0.26 (0.05)** | 0.00 (0.00) | 0.00 (0.00) |
| persistent | 0.00 (0.00) | 0.00 (0.00) | **0.35 (0.04)** | 0.00 (0.00) | 0.00 (0.00) |
| tempers | 0.00 (0.00) | 0.00 (0.00) | 0.00 (0.00) | **0.49 (0.04)** | 0.00 (0.00) |
| obedient | 0.00 (0.00) | 0.00 (0.00) | 0.00 (0.00) | **0.66 (0.03)** | 0.00 (0.00) |
| fights | 0.00 (0.00) | 0.00 (0.00) | 0.00 (0.00) | **0.65 (0.03)** | 0.00 (0.00) |
| lies | 0.00 (0.00) | 0.00 (0.00) | 0.00 (0.00) | **0.66 (0.03)** | 0.00 (0.00) |
| steals | 0.00 (0.00) | 0.00 (0.00) | 0.00 (0.00) | **0.67 (0.03)** | 0.00 (0.00) |
| somatic | 0.00 (0.00) | 0.00 (0.00) | 0.00 (0.00) | 0.00 (0.00) | **0.34 (0.04)** |
| worries | 0.00 (0.00) | 0.00 (0.00) | 0.00 (0.00) | 0.00 (0.00) | **0.70 (0.03)** |
| unhappy | 0.00 (0.00) | 0.00 (0.00) | 0.00 (0.00) | 0.00 (0.00) | **0.85 (0.03)** |
| clingy | 0.00 (0.00) | 0.00 (0.00) | 0.00 (0.00) | 0.00 (0.00) | **0.52 (0.04)** |
| fears | 0.00 (0.00) | 0.00 (0.00) | 0.00 (0.00) | 0.00 (0.00) | **0.46 (0.04)** |
|  |  |  |  |  |  |
| **Factor Correlations** |  |  |  |  |  |
| Prosocial behaviour x Peer Problems | 0.78 (0.03) |  |  |  |  |
| Prosocial behaviour x Hyperactivity | 0.32 (0.05) |  |  |  |  |
| Prosocial behaviour x Conduct Problems | 0.56 (0.04) |  |  |  |  |
| Prosocial behaviour x Emotional Problems | 0.31 (0.05) |  |  |  |  |
| Peer Problems x Hyperactivity | 0.09 (0.05) |  |  |  |  |
| Peer Problems x Conduct Problems | 0.26 (0.05) |  |  |  |  |
| Peer Problems x Emotional Problems | 0.28 (0.05) |  |  |  |  |
| Hyperactivity x Conduct Problems | 0.61 (0.04) |  |  |  |  |
| Hyperactivity x Emotional Problems | 0.43 (0.05) |  |  |  |  |
| Conduct Problems x Emotional Problems | 0.47 (0.05) |  |  |  |  |

Note. Table reports estimates and standard errors (Estimates (SE)). The factor loadings on the theoretical SDQ factors are highlighted in bold.

Supplementary Table 16. *Unrestricted factor model of five-factor SDQ structure in LSIC Wave 3K*

| **Factor Loadings** |  |  |  |  |  |
| --- | --- | --- | --- | --- | --- |
|  | *Factor 1* | *Factor 2* | *Factor 3* | *Factor 4* | *Factor 5* |
| considerate | 0.17 (0.07) | **0.50 (0.04)** | -0.06 (0.08) | 0.01 (0.09) | 0.21 (0.10) |
| shares | **0.40 (0.05)** | **0.52 (0.06)** | -0.07 (0.10) | -0.13 (0.10) | 0.35 (0.12) |
| caring | 0.29 (0.04) | **0.66 (0.04)** | -0.04 (0.07) | 0.00 (0.07) | 0.03 (0.09) |
| kind | 0.27 (0.01) | **0.72 (0.05)** | 0.14 (0.00) | -0.01 (0.00) | -0.16 (0.01) |
| helps | 0.23 (0.04) | **0.57 (0.04)** | 0.00 (0.07) | 0.04 (0.07) | 0.02 (0.09) |
| solitary | 0.06 (0.04) | -0.02 (0.05) | 0.27 (0.07) | 0.07 (0.07) | 0.07 (0.09) |
| friend | **1.93 (0.58)** | -0.05 (0.00) | -0.02 (0.00) | -0.02 (0.00) | 0.06 (0.00) |
| popular | 0.39 (0.06) | **0.65 (0.08)** | 0.18 (0.06) | -0.09 (0.06) | -0.16 (0.08) |
| bullied | 0.04 (0.05) | 0.09 (0.05) | 0.30 (0.07) | 0.12 (0.08) | 0.19 (0.09) |
| prefer adults | 0.14 (0.00) | -0.11 (0.00) | -0.01 (0.00) | 0.07 (0.00) | **0.47 (0.04)** |
| restless | -0.11 (0.05) | 0.05 (0.07) | 0.19 (0.10) | **0.76 (0.08)** | -0.04 (0.12) |
| fidgety | -0.02 (0.00) | -0.11 (0.00) | 0.05 (0.00) | **0.59 (0.04)** | 0.31 (0.01) |
| distractible | 0.01 (0.04) | -0.07 (0.05) | 0.06 (0.07) | 0.34 (0.07) | 0.35 (0.08) |
| reflective | 0.10 (0.05) | **0.46 (0.06)** | -0.03 (0.08) | 0.28 (0.08) | -0.19 (0.10) |
| persistent | 0.14 (0.09) | **0.40 (0.10)** | 0.21 (0.10) | **0.44 (0.09)** | -0.33 (0.12) |
| tempers | 0.22 (0.07) | 0.04 (0.08) | -0.10 (0.12) | -0.11 (0.12) | **0.64 (0.14)** |
| obedient | 0.38 (0.07) | **0.54 (0.06)** | -0.17 (0.12) | -0.09 (0.12) | **0.52 (0.13)** |
| fights | 0.30 (0.09) | 0.18 (0.09) | -0.05 (0.14) | -0.18 (0.14) | **0.73 (0.16)** |
| lies | 0.34 (0.15) | 0.15 (0.15) | -0.39 (0.22) | -0.39 (0.20) | **1.12 (0.23)** |
| steals | 0.30 (0.15) | 0.15 (0.14) | -0.36 (0.20) | -0.33 (0.19) | **1.07 (0.22)** |
| somatic | -0.01 (0.04) | 0.00 (0.05) | **0.42 (0.07)** | 0.12 (0.08) | -0.14 (0.10) |
| worries | -0.02 (0.00) | -0.05 (0.00) | **0.80 (0.03)** | 0.01 (0.00) | -0.12 (0.00) |
| unhappy | 0.08 (0.04) | 0.13 (0.05) | **0.77 (0.06)** | -0.01 (0.07) | 0.04 (0.09) |
| clingy | 0.03 (0.04) | -0.09 (0.05) | **0.40 (0.06)** | 0.12 (0.08) | 0.20 (0.09) |
| fears | -0.04 (0.05) | -0.16 (0.05) | **0.59 (0.07)** | 0.11 (0.08) | -0.10 (0.10) |
|  |  |  |  |  |  |
| **Factor Correlations** |  |  |  |  |  |
| Factor 1 x Factor 2 | -0.04 (0.12) |  |  |  |  |
| Factor 1 x Factor 3 | 0.04 (0.04) |  |  |  |  |
| Factor 1 x Factor 4 | 0.14 (0.04) |  |  |  |  |
| Factor 1 x Factor 5 | -0.27 (0.08) |  |  |  |  |
| Factor 2 x Factor 3 | 0.2 (0.07) |  |  |  |  |
| Factor 3 x Factor 4 | 0.22 (0.10) |  |  |  |  |
| Factor 4 x Factor 5 | 0.25 (0.12) |  |  |  |  |
| Factor 3 x Factor 4 | 0.14 (0.10) |  |  |  |  |
| Factor 3 x Factor 5 | 0.58 (0.09) |  |  |  |  |
| Factor 4 x Factor 5 | 0.55 (0.13) |  |  |  |  |

Note. Table reports estimates and standard errors (Estimates (SE). Substantive factor loadings (>.40) are highlighted in bold.

Supplementary Table 17. *Restricted factor model of three-factor SDQ structure in LSIC Wave 3K*

| **Factor Loadings** |  |  |  |
| --- | --- | --- | --- |
|  | *Prosocial behaviour* | *Internalizing* | *Externalizing* |
| considerate | **0.61 (0.03)** | 0.00 (0.00) | 0.00 (0.00) |
| shares | **0.65 (0.03)** | 0.00 (0.00) | 0.00 (0.00) |
| caring | **0.75 (0.03)** | 0.00 (0.00) | 0.00 (0.00) |
| kind | **0.70 (0.03)** | 0.00 (0.00) | 0.00 (0.00) |
| helps | **0.64 (0.03)** | 0.00 (0.00) | 0.00 (0.00) |
| solitary | 0.00 (0.00) | **0.34 (0.04)** | 0.00 (0.00) |
| friend | 0.00 (0.00) | **0.27 (0.05)** | 0.00 (0.00) |
| popular | 0.00 (0.00) | **0.34 (0.04)** | 0.00 (0.00) |
| bullied | 0.00 (0.00) | **0.50 (0.04)** | 0.00 (0.00) |
| prefer adults | 0.00 (0.00) | **0.30 (0.04)** | 0.00 (0.00) |
| somatic | 0.00 (0.00) | **0.36 (0.04)** | 0.00 (0.00) |
| worries | 0.00 (0.00) | **0.65 (0.03)** | 0.00 (0.00) |
| unhappy | 0.00 (0.00) | **0.82 (0.02)** | 0.00 (0.00) |
| clingy | 0.00 (0.00) | **0.52 (0.04)** | 0.00 (0.00) |
| fears | 0.00 (0.00) | **0.46 (0.04)** | 0.00 (0.00) |
| restless | 0.00 (0.00) | 0.00 (0.00) | **0.58 (0.03)** |
| fidgety | 0.00 (0.00) | 0.00 (0.00) | **0.59 (0.03)** |
| distractible | 0.00 (0.00) | 0.00 (0.00) | **0.53 (0.04)** |
| reflective | 0.00 (0.00) | 0.00 (0.00) | **0.31 (0.04)** |
| persistent | 0.00 (0.00) | 0.00 (0.00) | **0.38 (0.04)** |
| tempers | 0.00 (0.00) | 0.00 (0.00) | **0.47 (0.04)** |
| obedient | 0.00 (0.00) | 0.00 (0.00) | **0.64 (0.03)** |
| fights | 0.00 (0.00) | 0.00 (0.00) | **0.60 (0.03)** |
| lies | 0.00 (0.00) | 0.00 (0.00) | **0.60 (0.03)** |
| steals | 0.00 (0.00) | 0.00 (0.00) | **0.61 (0.03)** |
| **Factor Correlations** |  |  |  |
| Prosocial behaviour x Internalizing | **0.41 (0.05)** |  |  |
| Prosocial behaviour x Externalizing | **0.55 (0.04)** |  |  |
| Internalizing x Externalizing | **0.59 (0.04)** |  |  |

Note. Table reports estimates and standard errors (Estimates (SE)). The factor loadings on the theoretical SDQ factors are highlighted in bold.

Supplementary Table 18. *Unrestricted factor model of three-factor SDQ structure in LSIC Wave 3K*

| **Factor Loadings** |  |  |  |
| --- | --- | --- | --- |
|  | *Factor 1* | *Factor 2* | *Factor 3* |
| considerate | 0.38 (0.04) | 0.33 (0.05) | -0.01 (0.05) |
| shares | **0.55 (0.04)** | 0.28 (0.04) | 0.05 (0.05) |
| caring | **0.63 (0.03)** | 0.19 (0.05) | -0.06 (0.05) |
| kind | **0.69 (0.03)** | 0.07 (0.05) | 0.04 (0.05) |
| helps | **0.54 (0.04)** | 0.20 (0.05) | -0.02 (0.05) |
| solitary | 0.06 (0.05) | 0.06 (0.05) | 0.32 (0.05) |
| friend | 0.83 (0.02) | -0.21 (0.04) | 0.11 (0.04) |
| popular | **0.84 (0.02)** | -0.14 (0.00) | 0.12 (0.00) |
| bullied | **0.06 (0.05)** | 0.26 (0.05) | 0.37 (0.05) |
| adults | -0.07 (0.05) | 0.33 (0.05) | 0.20 (0.05) |
| restless | 0.02 (0.05) | **0.49 (0.04)** | 0.18 (0.05) |
| fidgety | -0.11 (0.05) | **0.57 (0.04)** | 0.20 (0.05) |
| distractible | -0.15 (0.05) | **0.52 (0.04)** | 0.21 (0.05) |
| reflective | **0.44 (0.04)** | 0.16 (0.05) | -0.11 (0.05) |
| persistent | **0.54 (0.04)** | 0.08 (0.05) | 0.10 (0.05) |
| tempers | -0.01 (0.05) | **0.43 (0.05)** | 0.14 (0.05) |
| obedient | **0.46 (0.04)** | **0.48 (0.04)** | 0.00 (0.05) |
| fights | 0.11 (0.05) | **0.49 (0.04)** | 0.22 (0.05) |
| lies | -0.01 (0.05) | **0.63 (0.04)** | 0.04 (0.06) |
| steals | -0.01 (0.00) | **0.64 (0.03)** | 0.04 (0.00) |
| somatic | 0.07 (0.05) | -0.05 (0.05) | 0.38 (0.05) |
| worries | 0.01 (0.00) | -0.11 (0.00) | **0.74 (0.03)** |
| unhappy | 0.16 (0.04) | 0.07 (0.05) | **0.73 (0.03)** |
| clingy | -0.08 (0.05) | 0.22 (0.05) | **0.48 (0.04)** |
| fears | -0.08 (0.05) | -0.07 (0.05) | **0.58 (0.04)** |
| **Factor Correlations** |  |  |  |
| Factor 1 x Factor 2 | 0.18 (0.07) |  |  |
| Factor 1 x Factor 3 | 0.12 (0.07) |  |  |
| Factor 2 x Factor 3 | 0.30 (0.08) |  |  |

Note. Table reports estimates and standard errors (Estimates (SE)). Substantive factor loadings (>.40) are highlighted in bold.

Supplementary Table 19. *Restricted factor model of the proposed four-factor SDQ structure in LSIC Wave 3K*

| **Factor Loadings** |  |  |  |  |
| --- | --- | --- | --- | --- |
|  | *Factor 1* | *Factor 2* | *Factor 3* | *Factor 4* |
| considerate | **0.51 (0.04)** | 0.00 (0.00) | 0.00 (0.00) | 0.00 (0.00) |
| shares | **0.65 (0.03)** | 0.00 (0.00) | 0.00 (0.00) | 0.00 (0.00) |
| caring | **0.69 (0.03)** | 0.00 (0.00) | 0.00 (0.00) | 0.00 (0.00) |
| kind | **0.73 (0.02)** | 0.00 (0.00) | 0.00 (0.00) | 0.00 (0.00) |
| helps | **0.62 (0.03)** | 0.00 (0.00) | 0.00 (0.00) | 0.00 (0.00) |
| obedient | **0.61 (0.03)** | 0.00 (0.00) | 0.00 (0.00) | 0.00 (0.00) |
| friend | **0.73 (0.02)** | 0.00 (0.00) | 0.00 (0.00) | 0.00 (0.00) |
| popular | **0.77 (0.02)** | 0.00 (0.00) | 0.00 (0.00) | 0.00 (0.00) |
| reflective | **0.47 (0.04)** | 0.00 (0.00) | 0.00 (0.00) | 0.00 (0.00) |
| persistent | **0.58 (0.03)** | 0.00 (0.00) | 0.00 (0.00) | 0.00 (0.00) |
| solitary | 0.00 (0.00) | **0.28 (0.05)** | 0.00 (0.00) | 0.00 (0.00) |
| bullied | 0.00 (0.00) | **0.52 (0.04)** | 0.00 (0.00) | 0.00 (0.00) |
| adults | 0.00 (0.00) | **0.49 (0.04)** | 0.00 (0.00) | 0.00 (0.00) |
| tempers | 0.00 (0.00) | **0.49 (0.04)** | 0.00 (0.00) | 0.00 (0.00) |
| fights | 0.00 (0.00) | **0.70 (0.03)** | 0.00 (0.00) | 0.00 (0.00) |
| steals | 0.00 (0.00) | **0.63 (0.03)** | 0.00 (0.00) | 0.00 (0.00) |
| lies | 0.00 (0.00) | **0.63 (0.03)** | 0.00 (0.00) | 0.00 (0.00) |
| unhappy | 0.00 (0.00) | 0.00 (0.00) | **0.86 (0.03)** | 0.00 (0.00) |
| clingy | 0.00 (0.00) | 0.00 (0.00) | **0.52 (0.04)** | 0.00 (0.00) |
| fears | 0.00 (0.00) | 0.00 (0.00) | **0.46 (0.04)** | 0.00 (0.00) |
| worries | 0.00 (0.00) | 0.00 (0.00) | **0.70 (0.03)** | 0.00 (0.00) |
| somatic | 0.00 (0.00) | 0.00 (0.00) | **0.34 (0.04)** | 0.00 (0.00) |
| restless | 0.00 (0.00) | 0.00 (0.00) | 0.00 (0.00) | **0.75 (0.03)** |
| fidgety | 0.00 (0.00) | 0.00 (0.00) | 0.00 (0.00) | **0.81 (0.03)** |
| distractible | 0.00 (0.00) | 0.00 (0.00) | 0.00 (0.00) | **0.59 (0.04)** |
|  |  |  |  |  |
| **Factor Correlations** |  |  |  |  |
| Factor 1 x Factor 2 | 0.36 (0.05) |  |  |  |
| Factor 1 x Factor 3 | 0.32 (0.05) |  |  |  |
| Factor 1 x Factor 4 | 0.20 (0.05) |  |  |  |
| Factor 2 x Factor 3 | 0.54 (0.04) |  |  |  |
| Factor 2 x Factor 4 | 0.66 (0.04) |  |  |  |
| Factor 3 x Factor 4 | 0.43 (0.05) |  |  |  |

Note. Table reports estimates and standard errors (Estimates (SE)). The factor loadings on the theoretical SDQ factors are highlighted in bold.

Supplementary Table 20. *Restricted factor model of five-factor SDQ structure in LSIC Wave 4K*

| **Factor Loadings** |  |  |  |  |  |
| --- | --- | --- | --- | --- | --- |
|  | *Prosocial behaviour* | *Peer Problems* | *Hyperactivity* | *Conduct Problems* | *Emotional Problems* |
| considerate | **0.75 (0.03)** | 0.00 (0.00) | 0.00 (0.00) | 0.00 (0.00) | 0.00 (0.00) |
| shares | **0.60 (0.03)** | 0.00 (0.00) | 0.00 (0.00) | 0.00 (0.00) | 0.00 (0.00) |
| caring | **0.76 (0.02)** | 0.00 (0.00) | 0.00 (0.00) | 0.00 (0.00) | 0.00 (0.00) |
| kind | **0.74 (0.03)** | 0.00 (0.00) | 0.00 (0.00) | 0.00 (0.00) | 0.00 (0.00) |
| helps | **0.52 (0.04)** | 0.00 (0.00) | 0.00 (0.00) | 0.00 (0.00) | 0.00 (0.00) |
| solitary | 0.00 (0.00) | **0.36 (0.05)** | 0.00 (0.00) | 0.00 (0.00) | 0.00 (0.00) |
| friend | 0.00 (0.00) | **0.46 (0.04)** | 0.00 (0.00) | 0.00 (0.00) | 0.00 (0.00) |
| popular | 0.00 (0.00) | **0.67 (0.04)** | 0.00 (0.00) | 0.00 (0.00) | 0.00 (0.00) |
| bullied | 0.00 (0.00) | **0.31 (0.05)** | 0.00 (0.00) | 0.00 (0.00) | 0.00 (0.00) |
| prefer adults | 0.00 (0.00) | **0.24 (0.05)** | 0.00 (0.00) | 0.00 (0.00) | 0.00 (0.00) |
| restless | 0.00 (0.00) | 0.00 (0.00) | **0.75 (0.03)** | 0.00 (0.00) | 0.00 (0.00) |
| fidgety | 0.00 (0.00) | 0.00 (0.00) | **0.88 (0.02)** | 0.00 (0.00) | 0.00 (0.00) |
| distractible | 0.00 (0.00) | 0.00 (0.00) | **0.59 (0.03)** | 0.00 (0.00) | 0.00 (0.00) |
| reflective | 0.00 (0.00) | 0.00 (0.00) | **0.31 (0.04)** | 0.00 (0.00) | 0.00 (0.00) |
| persistent | 0.00 (0.00) | 0.00 (0.00) | **0.41 (0.04)** | 0.00 (0.00) | 0.00 (0.00) |
| tempers | 0.00 (0.00) | 0.00 (0.00) | 0.00 (0.00) | **0.50 (0.04)** | 0.00 (0.00) |
| obedient | 0.00 (0.00) | 0.00 (0.00) | 0.00 (0.00) | **0.61 (0.04)** | 0.00 (0.00) |
| fights | 0.00 (0.00) | 0.00 (0.00) | 0.00 (0.00) | **0.50 (0.04)** | 0.00 (0.00) |
| lies | 0.00 (0.00) | 0.00 (0.00) | 0.00 (0.00) | **0.58 (0.04)** | 0.00 (0.00) |
| steals | 0.00 (0.00) | 0.00 (0.00) | 0.00 (0.00) | **0.33 (0.05)** | 0.00 (0.00) |
| somatic | 0.00 (0.00) | 0.00 (0.00) | 0.00 (0.00) | 0.00 (0.00) | **0.47 (0.04)** |
| worries | 0.00 (0.00) | 0.00 (0.00) | 0.00 (0.00) | 0.00 (0.00) | **0.54 (0.04)** |
| unhappy | 0.00 (0.00) | 0.00 (0.00) | 0.00 (0.00) | 0.00 (0.00) | **0.69 (0.03)** |
| clingy | 0.00 (0.00) | 0.00 (0.00) | 0.00 (0.00) | 0.00 (0.00) | **0.41 (0.04)** |
| fears | 0.00 (0.00) | 0.00 (0.00) | 0.00 (0.00) | 0.00 (0.00) | **0.67 (0.03)** |
|  |  |  |  |  |  |
| **Factor Correlations** |  |  |  |  |  |
| Prosocial behaviour x Peer Problems | 0.84 (0.04) |  |  |  |  |
| Prosocial behaviour x Hyperactivity | 0.34 (0.05) |  |  |  |  |
| Prosocial behaviour x Conduct Problems | 0.73 (0.04) |  |  |  |  |
| Prosocial behaviour x Emotional Problems | 0.23 (0.06) |  |  |  |  |
| Peer Problems x Hyperactivity | 0.26 (0.06) |  |  |  |  |
| Peer Problems x Conduct Problems | 0.62 (0.06) |  |  |  |  |
| Peer Problems x Emotional Problems | 0.6 (0.06) |  |  |  |  |
| Hyperactivity x Conduct Problems | 0.65 (0.04) |  |  |  |  |
| Hyperactivity x Emotional Problems | 0.49 (0.05) |  |  |  |  |
| Conduct Problems x Emotional Problems | 0.57 (0.05) |  |  |  |  |

Note. Table reports estimates and standard errors (Estimates (SE)). The factor loadings on the theoretical SDQ factors are highlighted in bold.

Supplementary Table 21. *Restricted factor model of three-factor SDQ structure in LSIC Wave 4K*

| **Factor Loadings** |  |  |  |
| --- | --- | --- | --- |
|  | *Prosocial behaviour* | *Internalizing* | *Externalizing* |
| considerate | **0.72 (0.03)** | 0.00 (0.00) | 0.00 (0.00) |
| shares | **0.59 (0.03)** | 0.00 (0.00) | 0.00 (0.00) |
| caring | **0.79 (0.02)** | 0.00 (0.00) | 0.00 (0.00) |
| kind | **0.72 (0.03)** | 0.00 (0.00) | 0.00 (0.00) |
| helps | **0.56 (0.04)** | 0.00 (0.00) | 0.00 (0.00) |
| solitary | 0.00 (0.00) | **0.28 (0.05)** | 0.00 (0.00) |
| friend | 0.00 (0.00) | **0.22 (0.05)** | 0.00 (0.00) |
| popular | 0.00 (0.00) | **0.48 (0.04)** | 0.00 (0.00) |
| bullied | 0.00 (0.00) | **0.47 (0.04)** | 0.00 (0.00) |
| prefer adults | 0.00 (0.00) | **0.29 (0.05)** | 0.00 (0.00) |
| somatic | 0.00 (0.00) | **0.42 (0.04)** | 0.00 (0.00) |
| worries | 0.00 (0.00) | **0.50 (0.04)** | 0.00 (0.00) |
| unhappy | 0.00 (0.00) | **0.72 (0.03)** | 0.00 (0.00) |
| clingy | 0.00 (0.00) | **0.38 (0.05)** | 0.00 (0.00) |
| fears | 0.00 (0.00) | **0.62 (0.04)** | 0.00 (0.00) |
| restless | 0.00 (0.00) | 0.00 (0.00) | **0.64 (0.03)** |
| fidgety | 0.00 (0.00) | 0.00 (0.00) | **0.76 (0.03)** |
| distractible | 0.00 (0.00) | 0.00 (0.00) | **0.58 (0.04)** |
| reflective | 0.00 (0.00) | 0.00 (0.00) | **0.38 (0.04)** |
| persistent | 0.00 (0.00) | 0.00 (0.00) | **0.48 (0.04)** |
| tempers | 0.00 (0.00) | 0.00 (0.00) | **0.45 (0.04)** |
| obedient | 0.00 (0.00) | 0.00 (0.00) | **0.50 (0.04)** |
| fights | 0.00 (0.00) | 0.00 (0.00) | **0.46 (0.04)** |
| lies | 0.00 (0.00) | 0.00 (0.00) | **0.53 (0.04)** |
| steals | 0.00 (0.00) | 0.00 (0.00) | **0.26 (0.05)** |
| **Factor Correlations** |  |  |  |
| Prosocial behaviour x Internalizing | 0.41 (0.05) |  |  |
| Prosocial behaviour x Externalizing | 0.53 (0.04) |  |  |
| Internalizing x Externalizing | 0.61 (0.04) |  |  |

Note. Table reports estimates and standard errors (Estimates (SE)). The factor loadings on the theoretical SDQ factors are highlighted in bold.

Supplementary Table 22. *Unrestricted factor model of three-factor SDQ structure in LSIC Wave 4K*

| **Factor Loadings** |  |  |  |
| --- | --- | --- | --- |
|  | *Factor 1* | *Factor 2* | *Factor 3* |
| considerate | **0.64 (0.03)** | 0.25 (0.04) | 0.04 (0.05) |
| shares | **0.57 (0.04)** | 0.00 (0.05) | 0.11 (0.05) |
| caring | **0.74 (0.03)** | -0.04 (0.05) | 0.13 (0.05) |
| kind | **0.78 (0.02)** | 0.04 (0.00) | -0.05 (0.00) |
| helps | **0.49 (0.04)** | 0.12 (0.05) | 0.07 (0.05) |
| solitary | 0.21 (0.05) | -0.12 (0.05) | 0.22 (0.05) |
| friend | **0.52 (0.04)** | 0.07 (0.05) | -0.04 (0.05) |
| popular | **0.49 (0.04)** | -0.19 (0.05) | **0.44 (0.05)** |
| bullied | 0.01 (0.05) | 0.20 (0.05) | 0.36 (0.05) |
| adults | 0.04 (0.05) | 0.13 (0.05) | 0.17 (0.05) |
| restless | -0.12 (0.04) | **0.74 (0.03)** | 0.06 (0.04) |
| fidgety | 0.00 (0.00) | **0.90 (0.02)** | 0.01 (0.00) |
| distractible | 0.03 (0.04) | **0.53 (0.04)** | 0.10 (0.05) |
| reflective | 0.37 (0.04) | 0.20 (0.05) | -0.03 (0.05) |
| persistent | 0.35 (0.04) | 0.22 (0.05) | 0.15 (0.05) |
| tempers | 0.18 (0.05) | 0.20 (0.05) | 0.30 (0.05) |
| obedient | **0.46 (0.04)** | 0.17 (0.05) | 0.21 (0.05) |
| fights | 0.18 (0.05) | 0.32 (0.05) | 0.13 (0.05) |
| lies | 0.19 (0.05) | 0.39 (0.04) | 0.11 (0.05) |
| steals | 0.20 (0.05) | 0.23 (0.05) | -0.18 (0.05) |
| somatic | -0.16 (0.05) | 0.16 (0.05) | **0.42 (0.05)** |
| worries | -0.08 (0.05) | -0.04 (0.05) | **0.61 (0.04)** |
| unhappy | 0.03 (0.00) | 0.06 (0.00) | **0.75 (0.03)** |
| clingy | -0.14 (0.05) | 0.22 (0.05) | 0.32 (0.05) |
| fears | -0.06 (0.05) | 0.20 (0.05) | **0.52 (0.04)** |
| **Factor Correlations** |  |  |  |
| Factor 1 x Factor 2 | 0.20 (0.06) |  |  |
| Factor 1 x Factor 3 | 0.20 (0.07) |  |  |
| Factor 2 x Factor 3 | 0.29 (0.06) |  |  |

Note. Table reports estimates and standard errors (Estimates (SE)). Substantive factor loadings (>.40) are highlighted in bold.

Supplementary Table 23. *Restricted factor model of the proposed four-factor SDQ structure in LSIC Wave 4K*

| **Factor Loadings** |  |  |  |  |
| --- | --- | --- | --- | --- |
|  | *Factor 1* | *Factor 2* | *Factor 3* | *Factor 4* |
| considerate | **0.74 (0.03)** | 0.00 (0.00) | 0.00 (0.00) | 0.00 (0.00) |
| shares | **0.60 (0.03)** | 0.00 (0.00) | 0.00 (0.00) | 0.00 (0.00) |
| caring | **0.74 (0.03)** | 0.00 (0.00) | 0.00 (0.00) | 0.00 (0.00) |
| kind | **0.72 (0.03)** | 0.00 (0.00) | 0.00 (0.00) | 0.00 (0.00) |
| helps | **0.56 (0.04)** | 0.00 (0.00) | 0.00 (0.00) | 0.00 (0.00) |
| obedient | **0.61 (0.03)** | 0.00 (0.00) | 0.00 (0.00) | 0.00 (0.00) |
| friend | **0.50 (0.04)** | 0.00 (0.00) | 0.00 (0.00) | 0.00 (0.00) |
| popular | **0.57 (0.03)** | 0.00 (0.00) | 0.00 (0.00) | 0.00 (0.00) |
| reflective | **0.44 (0.04)** | 0.00 (0.00) | 0.00 (0.00) | 0.00 (0.00) |
| persistent | **0.50 (0.04)** | 0.00 (0.00) | 0.00 (0.00) | 0.00 (0.00) |
| solitary | 0.00 (0.00) | **0.17 (0.05)** | 0.00 (0.00) | 0.00 (0.00) |
| bullied | 0.00 (0.00) | **0.51 (0.04)** | 0.00 (0.00) | 0.00 (0.00) |
| adults | 0.00 (0.00) | **0.29 (0.05)** | 0.00 (0.00) | 0.00 (0.00) |
| tempers | 0.00 (0.00) | **0.52 (0.04)** | 0.00 (0.00) | 0.00 (0.00) |
| fights | 0.00 (0.00) | **0.59 (0.04)** | 0.00 (0.00) | 0.00 (0.00) |
| steals | 0.00 (0.00) | **0.41 (0.05)** | 0.00 (0.00) | 0.00 (0.00) |
| lies | 0.00 (0.00) | **0.64 (0.04)** | 0.00 (0.00) | 0.00 (0.00) |
| unhappy | 0.00 (0.00) | 0.00 (0.00) | **0.72 (0.03)** | 0.00 (0.00) |
| clingy | 0.00 (0.00) | 0.00 (0.00) | **0.41 (0.05)** | 0.00 (0.00) |
| fears | 0.00 (0.00) | 0.00 (0.00) | **0.65 (0.04)** | 0.00 (0.00) |
| worries | 0.00 (0.00) | 0.00 (0.00) | **0.56 (0.04)** | 0.00 (0.00) |
| somatic | 0.00 (0.00) | 0.00 (0.00) | **0.44 (0.04)** | 0.00 (0.00) |
| restless | 0.00 (0.00) | 0.00 (0.00) | 0.00 (0.00) | **0.74 (0.03)** |
| fidgety | 0.00 (0.00) | 0.00 (0.00) | 0.00 (0.00) | **0.91 (0.02)** |
| distractible | 0.00 (0.00) | 0.00 (0.00) | 0.00 (0.00) | **0.57 (0.04)** |
|  |  |  |  |  |
| **Factor Correlations** |  |  |  |  |
| Factor 1 x Factor 2 | **0.53 (0.05)** |  |  |  |
| Factor 1 x Factor 3 | **0.32 (0.05)** |  |  |  |
| Factor 1 x Factor 4 | **0.34 (0.05)** |  |  |  |
| Factor 2 x Factor 3 | **0.57 (0.05)** |  |  |  |
| Factor 2 x Factor 4 | **0.60 (0.04)** |  |  |  |
| Factor 3 x Factor 4 | **0.47 (0.05)** |  |  |  |

Note. Table reports estimates and standard errors (Estimates (SE)). The factor loadings on the theoretical SDQ factors are highlighted in bold.

Supplementary Table 24. *Restricted factor model of five-factor SDQ structure in LSIC Wave 6*

| **Factor Loadings** |  |  |  |  |  |
| --- | --- | --- | --- | --- | --- |
|  | *Prosocial behaviour* | *Peer Problems* | *Hyperactivity* | *Conduct Problems* | *Emotional Problems* |
| considerate | **0.63 (0.02)** | 0.00 (0.00) | 0.00 (0.00) | 0.00 (0.00) | 0.00 (0.00) |
| shares | **0.67 (0.02)** | 0.00 (0.00) | 0.00 (0.00) | 0.00 (0.00) | 0.00 (0.00) |
| caring | **0.67 (0.02)** | 0.00 (0.00) | 0.00 (0.00) | 0.00 (0.00) | 0.00 (0.00) |
| kind | **0.78 (0.02)** | 0.00 (0.00) | 0.00 (0.00) | 0.00 (0.00) | 0.00 (0.00) |
| helps | **0.51 (0.03)** | 0.00 (0.00) | 0.00 (0.00) | 0.00 (0.00) | 0.00 (0.00) |
| solitary | 0.00 (0.00) | **0.34 (0.03)** | 0.00 (0.00) | 0.00 (0.00) | 0.00 (0.00) |
| friend | 0.00 (0.00) | **0.64 (0.02)** | 0.00 (0.00) | 0.00 (0.00) | 0.00 (0.00) |
| popular | 0.00 (0.00) | **0.81 (0.02)** | 0.00 (0.00) | 0.00 (0.00) | 0.00 (0.00) |
| bullied | 0.00 (0.00) | **0.39 (0.03)** | 0.00 (0.00) | 0.00 (0.00) | 0.00 (0.00) |
| prefer adults | 0.00 (0.00) | **0.35 (0.03)** | 0.00 (0.00) | 0.00 (0.00) | 0.00 (0.00) |
| restless | 0.00 (0.00) | 0.00 (0.00) | **0.81 (0.02)** | 0.00 (0.00) | 0.00 (0.00) |
| fidgety | 0.00 (0.00) | 0.00 (0.00) | **0.80 (0.02)** | 0.00 (0.00) | 0.00 (0.00) |
| distractible | 0.00 (0.00) | 0.00 (0.00) | **0.65 (0.02)** | 0.00 (0.00) | 0.00 (0.00) |
| reflective | 0.00 (0.00) | 0.00 (0.00) | **0.39 (0.03)** | 0.00 (0.00) | 0.00 (0.00) |
| persistent | 0.00 (0.00) | 0.00 (0.00) | **0.52 (0.03)** | 0.00 (0.00) | 0.00 (0.00) |
| tempers | 0.00 (0.00) | 0.00 (0.00) | 0.00 (0.00) | **0.65 (0.02)** | 0.00 (0.00) |
| obedient | 0.00 (0.00) | 0.00 (0.00) | 0.00 (0.00) | **0.65 (0.02)** | 0.00 (0.00) |
| fights | 0.00 (0.00) | 0.00 (0.00) | 0.00 (0.00) | **0.76 (0.02)** | 0.00 (0.00) |
| lies | 0.00 (0.00) | 0.00 (0.00) | 0.00 (0.00) | **0.65 (0.02)** | 0.00 (0.00) |
| steals | 0.00 (0.00) | 0.00 (0.00) | 0.00 (0.00) | **0.52 (0.03)** | 0.00 (0.00) |
| somatic | 0.00 (0.00) | 0.00 (0.00) | 0.00 (0.00) | 0.00 (0.00) | **0.51 (0.03)** |
| worries | 0.00 (0.00) | 0.00 (0.00) | 0.00 (0.00) | 0.00 (0.00) | **0.60 (0.02)** |
| unhappy | 0.00 (0.00) | 0.00 (0.00) | 0.00 (0.00) | 0.00 (0.00) | **0.78 (0.02)** |
| clingy | 0.00 (0.00) | 0.00 (0.00) | 0.00 (0.00) | 0.00 (0.00) | **0.50 (0.03)** |
| fears | 0.00 (0.00) | 0.00 (0.00) | 0.00 (0.00) | 0.00 (0.00) | **0.54 (0.03)** |
|  |  |  |  |  |  |
| **Factor Correlations** |  |  |  |  |  |
| Prosocial behaviour x Peer Problems | 0.79 (0.02) |  |  |  |  |
| Prosocial behaviour x Hyperactivity | 0.44 (0.03) |  |  |  |  |
| Prosocial behaviour x Conduct Problems | 0.61 (0.03) |  |  |  |  |
| Prosocial behaviour x Emotional Problems | 0.28 (0.04) |  |  |  |  |
| Peer Problems x Hyperactivity | 0.46 (0.03) |  |  |  |  |
| Peer Problems x Conduct Problems | 0.59 (0.03) |  |  |  |  |
| Peer Problems x Emotional Problems | 0.54 (0.03) |  |  |  |  |
| Hyperactivity x Conduct Problems | 0.73 (0.02) |  |  |  |  |
| Hyperactivity x Emotional Problems | 0.64 (0.03) |  |  |  |  |
| Conduct Problems x Emotional Problems | 0.59 (0.03) |  |  |  |  |

Note. Table reports estimates and standard errors (Estimates (SE)). The factor loadings on the theoretical SDQ factors are highlighted in bold.

Supplementary Table 25. *Unrestricted factor model of five-factor SDQ structure in LSIC Wave 6*

| **Factor Loadings** |  |  |  |  |  |
| --- | --- | --- | --- | --- | --- |
|  | *Factor 1* | *Factor 2* | *Factor 3* | *Factor 4* | *Factor 5* |
| considerate | 0.36 (0.04) | 0.10 (0.04) | 0.17 (0.04) | -0.05 (0.04) | 0.28 (0.04) |
| shares | 0.25 (0.01) | -0.03 (0.00) | 0.10 (0.00) | 0.27 (0.01) | **0.54 (0.03)** |
| caring | **0.53 (0.03)** | 0.05 (0.04) | 0.06 (0.04) | -0.10 (0.04) | 0.20 (0.04) |
| kind | **0.58 (0.03)** | -0.11 (0.04) | 0.18 (0.04) | -0.05 (0.04) | 0.32 (0.04) |
| helps | 0.22 (0.05) | 0.08 (0.05) | 0.05 (0.05) | -0.16 (0.04) | **0.40 (0.04)** |
| solitary | 0.13 (0.04) | -0.10 (0.04) | 0.00 (0.04) | **0.41 (0.04)** | 0.16 (0.05) |
| friend | **0.68 (0.02)** | 0.10 (0.00) | -0.11 (0.00) | 0.06 (0.00) | 0.06 (0.00) |
| popular | **0.66 (0.03)** | 0.03 (0.04) | 0.16 (0.04) | 0.12 (0.04) | 0.05 (0.04) |
| bullied | 0.19 (0.05) | 0.11 (0.05) | 0.34 (0.05) | 0.21 (0.05) | -0.23 (0.04) |
| prefer adults | 0.26 (0.04) | 0.14 (0.05) | 0.06 (0.05) | 0.24 (0.04) | -0.19 (0.04) |
| restless | 0.06 (0.00) | **0.81 (0.02)** | 0.06 (0.00) | 0.01 (0.00) | 0.00 (0.00) |
| fidgety | -0.08 (0.03) | **0.73 (0.03)** | 0.12 (0.04) | 0.10 (0.04) | 0.03 (0.03) |
| distractible | -0.04 (0.04) | **0.44 (0.04)** | 0.09 (0.04) | 0.11 (0.04) | 0.29 (0.04) |
| reflective | 0.14 (0.05) | 0.20 (0.05) | 0.02 (0.05) | -0.05 (0.05) | **0.44 (0.04)** |
| persistent | 0.02 (0.05) | 0.34 (0.05) | 0.01 (0.05) | 0.02 (0.05) | **0.44 (0.04)** |
| tempers | -0.15 (0.04) | 0.16 (0.04) | **0.41 (0.04)** | 0.24 (0.04) | 0.28 (0.04) |
| obedient | 0.13 (0.04) | 0.19 (0.04) | 0.31 (0.04) | -0.01 (0.04) | 0.38 (0.03) |
| fights | 0.00 (0.00) | 0.07 (0.00) | **0.76 (0.02)** | 0.03 (0.00) | 0.08 (0.00) |
| lies | 0.00 (0.04) | 0.15 (0.04) | **0.64 (0.04)** | -0.05 (0.04) | 0.00 (0.04) |
| steals | 0.16 (0.04) | 0.03 (0.04) | **0.46 (0.04)** | 0.05 (0.04) | -0.05 (0.04) |
| somatic | 0.02 (0.05) | 0.22 (0.05) | 0.06 (0.05) | 0.38 (0.04) | -0.18 (0.04) |
| worries | 0.10 (0.04) | 0.17 (0.05) | -0.06 (0.05) | **0.50 (0.04)** | 0.00 (0.05) |
| unhappy | 0.28 (0.05) | 0.29 (0.05) | 0.23 (0.05) | 0.37 (0.04) | -0.22 (0.04) |
| clingy | -0.15 (0.00) | 0.05 (0.00) | 0.01 (0.00) | **0.64 (0.03)** | 0.14 (0.00) |
| fears | -0.02 (0.05) | 0.07 (0.05) | 0.13 (0.05) | **0.49 (0.04)** | -0.05 (0.05) |
|  |  |  |  |  |  |
| **Factor Correlations** |  |  |  |  |  |
| Factor 1 x Factor 2 | 0.17 (0.05) |  |  |  |  |
| Factor 1 x Factor 3 | 0.29 (0.05) |  |  |  |  |
| Factor 1 x Factor 4 | 0.14 (0.07) |  |  |  |  |
| Factor 1 x Factor 5 | 0.33 (0.08) |  |  |  |  |
| Factor 2 x Factor 3 | 0.42 (0.05) |  |  |  |  |
| Factor 3 x Factor 4 | 0.35 (0.06) |  |  |  |  |
| Factor 4 x Factor 5 | 0.23 (0.07) |  |  |  |  |
| Factor 3 x Factor 4 | 0.27 (0.06) |  |  |  |  |
| Factor 3 x Factor 5 | 0.24 (0.08) |  |  |  |  |
| Factor 4 x Factor 5 | -0.01 (0.07) |  |  |  |  |

Note. Table reports estimates and standard errors (Estimates (SE). Substantive factor loadings (>.40) are highlighted in bold.

Supplementary Table 26. *Restricted factor model of three-factor SDQ structure in LSIC Wave 6*

| **Factor Loadings** |  |  |  |
| --- | --- | --- | --- |
|  | *Prosocial behaviour* | *Internalizing* | *Externalizing* |
| considerate | **0.64 (0.02)** | 0.00 (0.00) | 0.00 (0.00) |
| shares | **0.68 (0.02)** | 0.00 (0.00) | 0.00 (0.00) |
| caring | **0.68 (0.02)** | 0.00 (0.00) | 0.00 (0.00) |
| kind | **0.75 (0.02)** | 0.00 (0.00) | 0.00 (0.00) |
| helps | **0.52 (0.03)** | 0.00 (0.00) | 0.00 (0.00) |
| solitary | 0.00 (0.00) | **0.34 (0.03)** | 0.00 (0.00) |
| friend | 0.00 (0.00) | **0.43 (0.03)** | 0.00 (0.00) |
| popular | 0.00 (0.00) | **0.56 (0.02)** | 0.00 (0.00) |
| bullied | 0.00 (0.00) | **0.51 (0.03)** | 0.00 (0.00) |
| prefer adults | 0.00 (0.00) | **0.40 (0.03)** | 0.00 (0.00) |
| somatic | 0.00 (0.00) | **0.45 (0.03)** | 0.00 (0.00) |
| worries | 0.00 (0.00) | **0.54 (0.03)** | 0.00 (0.00) |
| unhappy | 0.00 (0.00) | **0.75 (0.02)** | 0.00 (0.00) |
| clingy | 0.00 (0.00) | **0.45 (0.03)** | 0.00 (0.00) |
| fears | 0.00 (0.00) | **0.49 (0.03)** | 0.00 (0.00) |
| restless | 0.00 (0.00) | 0.00 (0.00) | **0.71 (0.02)** |
| fidgety | 0.00 (0.00) | 0.00 (0.00) | **0.69 (0.02)** |
| distractible | 0.00 (0.00) | 0.00 (0.00) | **0.63 (0.02)** |
| reflective | 0.00 (0.00) | 0.00 (0.00) | **0.44 (0.03)** |
| persistent | 0.00 (0.00) | 0.00 (0.00) | **0.53 (0.02)** |
| tempers | 0.00 (0.00) | 0.00 (0.00) | **0.63 (0.02)** |
| obedient | 0.00 (0.00) | 0.00 (0.00) | **0.64 (0.02)** |
| fights | 0.00 (0.00) | 0.00 (0.00) | **0.67 (0.02)** |
| lies | 0.00 (0.00) | 0.00 (0.00) | **0.60 (0.02)** |
| steals | 0.00 (0.00) | 0.00 (0.00) | **0.46 (0.03)** |
| **Factor Correlations** |  |  |  |
| Prosocial behaviour x Internalizing | 0.49 (0.03) |  |  |
| Prosocial behaviour x Externalizing | 0.60 (0.03) |  |  |
| Internalizing x Externalizing | 0.72 (0.02) |  |  |

Note. Table reports estimates and standard errors (Estimates (SE)). The factor loadings on the theoretical SDQ factors are highlighted in bold.

Supplementary Table 27. *Unrestricted factor model of three-factor SDQ structure in LSIC Wave 6*

| **Factor Loadings** |  |  |  |
| --- | --- | --- | --- |
|  | *Factor 1* | *Factor 2* | *Factor 3* |
| considerate | **0.52 (0.03)** | 0.23 (0.04) | -0.02 (0.04) |
| shares | **0.53 (0.03)** | 0.25 (0.03) | 0.06 (0.03) |
| caring | **0.63 (0.03)** | 0.04 (0.04) | -0.02 (0.04) |
| kind | **0.79 (0.02)** | 0.02 (0.00) | -0.03 (0.00) |
| helps | **0.43 (0.04)** | 0.26 (0.04) | -0.25 (0.04) |
| solitary | 0.21 (0.04) | -0.03 (0.04) | 0.28 (0.04) |
| friend | **0.61 (0.03)** | -0.07 (0.04) | 0.17 (0.04) |
| popular | **0.69 (0.03)** | -0.04 (0.04) | 0.29 (0.03) |
| bullied | 0.12 (0.04) | 0.06 (0.04) | **0.47 (0.03)** |
| adults | 0.14 (0.04) | -0.05 (0.04) | **0.42 (0.03)** |
| restless | -0.05 (0.03) | **0.65 (0.03)** | 0.26 (0.03) |
| fidgety | -0.15 (0.00) | **0.68 (0.02)** | 0.28 (0.01) |
| distractible | 0.05 (0.04) | **0.61 (0.03)** | 0.07 (0.04) |
| reflective | 0.32 (0.04) | **0.42 (0.04)** | -0.18 (0.04) |
| persistent | 0.18 (0.04) | **0.57 (0.03)** | -0.12 (0.04) |
| tempers | 0.07 (0.03) | **0.49 (0.03)** | 0.21 (0.04) |
| obedient | 0.36 (0.03) | **0.48 (0.03)** | -0.02 (0.04) |
| fights | 0.21 (0.03) | **0.41 (0.03)** | 0.25 (0.03) |
| lies | 0.13 (0.03) | **0.40 (0.03)** | 0.20 (0.04) |
| steals | 0.21 (0.03) | 0.18 (0.04) | 0.25 (0.04) |
| somatic | -0.11 (0.04) | 0.10 (0.04) | **0.50 (0.03)** |
| worries | 0.03 (0.04) | 0.10 (0.04) | **0.48 (0.03)** |
| unhappy | 0.14 (0.00) | 0.14 (0.00) | **0.65 (0.02)** |
| clingy | -0.09 (0.04) | 0.16 (0.04) | **0.44 (0.03)** |
| fears | -0.04 (0.04) | 0.09 (0.04) | **0.49 (0.03)** |
| **Factor Correlations** |  |  |  |
| Factor 1 x Factor 2 | 0.34 (0.05) |  |  |
| Factor 1 x Factor 3 | 0.14 (0.06) |  |  |
| Factor 2 x Factor 3 | 0.34 (0.06) |  |  |

Note. Table reports estimates and standard errors (Estimates (SE)). Substantive factor loadings (>.40) are highlighted in bold.

Supplementary Table 28. *Restricted factor model of the proposed four-factor SDQ structure in LSIC Wave 6*

| **Factor Loadings** |  |  |  |  |
| --- | --- | --- | --- | --- |
|  | *Factor 1* | *Factor 2* | *Factor 3* | *Factor 4* |
| considerate | **0.64 (0.02)** | 0.00 (0.00) | 0.00 (0.00) | 0.00 (0.00) |
| shares | **0.68 (0.02)** | 0.00 (0.00) | 0.00 (0.00) | 0.00 (0.00) |
| caring | **0.61 (0.02)** | 0.00 (0.00) | 0.00 (0.00) | 0.00 (0.00) |
| kind | **0.73 (0.02)** | 0.00 (0.00) | 0.00 (0.00) | 0.00 (0.00) |
| helps | **0.49 (0.03)** | 0.00 (0.00) | 0.00 (0.00) | 0.00 (0.00) |
| obedient | **0.63 (0.02)** | 0.00 (0.00) | 0.00 (0.00) | 0.00 (0.00) |
| friend | **0.58 (0.02)** | 0.00 (0.00) | 0.00 (0.00) | 0.00 (0.00) |
| popular | **0.70 (0.02)** | 0.00 (0.00) | 0.00 (0.00) | 0.00 (0.00) |
| reflective | **0.52 (0.03)** | 0.00 (0.00) | 0.00 (0.00) | 0.00 (0.00) |
| persistent | **0.49 (0.03)** | 0.00 (0.00) | 0.00 (0.00) | 0.00 (0.00) |
| solitary | 0.00 (0.00) | **0.27 (0.03)** | 0.00 (0.00) | 0.00 (0.00) |
| bullied | 0.00 (0.00) | **0.49 (0.03)** | 0.00 (0.00) | 0.00 (0.00) |
| adults | 0.00 (0.00) | **0.32 (0.03)** | 0.00 (0.00) | 0.00 (0.00) |
| tempers | 0.00 (0.00) | **0.64 (0.02)** | 0.00 (0.00) | 0.00 (0.00) |
| fights | 0.00 (0.00) | **0.76 (0.02)** | 0.00 (0.00) | 0.00 (0.00) |
| steals | 0.00 (0.00) | **0.54 (0.03)** | 0.00 (0.00) | 0.00 (0.00) |
| lies | 0.00 (0.00) | **0.66 (0.02)** | 0.00 (0.00) | 0.00 (0.00) |
| unhappy | 0.00 (0.00) | 0.00 (0.00) | **0.77 (0.02)** | 0.00 (0.00) |
| clingy | 0.00 (0.00) | 0.00 (0.00) | **0.50 (0.03)** | 0.00 (0.00) |
| fears | 0.00 (0.00) | 0.00 (0.00) | **0.55 (0.03)** | 0.00 (0.00) |
| worries | 0.00 (0.00) | 0.00 (0.00) | **0.59 (0.02)** | 0.00 (0.00) |
| somatic | 0.00 (0.00) | 0.00 (0.00) | **0.51 (0.03)** | 0.00 (0.00) |
| restless | 0.00 (0.00) | 0.00 (0.00) | 0.00 (0.00) | **0.84 (0.01)** |
| fidgety | 0.00 (0.00) | 0.00 (0.00) | 0.00 (0.00) | **0.84 (0.01)** |
| distractible | 0.00 (0.00) | 0.00 (0.00) | 0.00 (0.00) | **0.59 (0.02)** |
|  |  |  |  |  |
| **Factor Correlations** |  |  |  |  |
| Factor 1 x Factor 2 | 0.63 (0.03) |  |  |  |
| Factor 1 x Factor 3 | 0.37 (0.03) |  |  |  |
| Factor 1 x Factor 4 | 0.47 (0.03) |  |  |  |
| Factor 2 x Factor 3 | 0.67 (0.03) |  |  |  |
| Factor 2 x Factor 4 | 0.69 (0.02) |  |  |  |
| Factor 3 x Factor 4 | 0.66 (0.03) |  |  |  |

Note. Table reports estimates and standard errors (Estimates (SE)). The factor loadings on the theoretical SDQ factors are highlighted in bold.

Supplementary Table 29. *Restricted factor model of five-factor SDQ structure in LSIC Wave 8*

| **Factor Loadings** |  |  |  |  |  |
| --- | --- | --- | --- | --- | --- |
|  | *Prosocial behaviour* | *Peer Problems* | *Hyperactivity* | *Conduct Problems* | *Emotional Problems* |
| considerate | **0.64 (0.02)** | 0.00 (0.00) | 0.00 (0.00) | 0.00 (0.00) | 0.00 (0.00) |
| shares | **0.58 (0.02)** | 0.00 (0.00) | 0.00 (0.00) | 0.00 (0.00) | 0.00 (0.00) |
| caring | **0.73 (0.02)** | 0.00 (0.00) | 0.00 (0.00) | 0.00 (0.00) | 0.00 (0.00) |
| kind | **0.79 (0.02)** | 0.00 (0.00) | 0.00 (0.00) | 0.00 (0.00) | 0.00 (0.00) |
| helps | **0.53 (0.03)** | 0.00 (0.00) | 0.00 (0.00) | 0.00 (0.00) | 0.00 (0.00) |
| solitary | 0.00 (0.00) | **0.40 (0.03)** | 0.00 (0.00) | 0.00 (0.00) | 0.00 (0.00) |
| friend | 0.00 (0.00) | **0.69 (0.02)** | 0.00 (0.00) | 0.00 (0.00) | 0.00 (0.00) |
| popular | 0.00 (0.00) | **0.76 (0.02)** | 0.00 (0.00) | 0.00 (0.00) | 0.00 (0.00) |
| bullied | 0.00 (0.00) | **0.39 (0.03)** | 0.00 (0.00) | 0.00 (0.00) | 0.00 (0.00) |
| prefer adults | 0.00 (0.00) | **0.23 (0.03)** | 0.00 (0.00) | 0.00 (0.00) | 0.00 (0.00) |
| restless | 0.00 (0.00) | 0.00 (0.00) | **0.83 (0.02)** | 0.00 (0.00) | 0.00 (0.00) |
| fidgety | 0.00 (0.00) | 0.00 (0.00) | **0.82 (0.02)** | 0.00 (0.00) | 0.00 (0.00) |
| distractible | 0.00 (0.00) | 0.00 (0.00) | **0.67 (0.02)** | 0.00 (0.00) | 0.00 (0.00) |
| reflective | 0.00 (0.00) | 0.00 (0.00) | **0.36 (0.03)** | 0.00 (0.00) | 0.00 (0.00) |
| persistent | 0.00 (0.00) | 0.00 (0.00) | **0.46 (0.03)** | 0.00 (0.00) | 0.00 (0.00) |
| tempers | 0.00 (0.00) | 0.00 (0.00) | 0.00 (0.00) | **0.65 (0.02)** | 0.00 (0.00) |
| obedient | 0.00 (0.00) | 0.00 (0.00) | 0.00 (0.00) | **0.56 (0.03)** | 0.00 (0.00) |
| fights | 0.00 (0.00) | 0.00 (0.00) | 0.00 (0.00) | **0.78 (0.02)** | 0.00 (0.00) |
| lies | 0.00 (0.00) | 0.00 (0.00) | 0.00 (0.00) | **0.64 (0.02)** | 0.00 (0.00) |
| steals | 0.00 (0.00) | 0.00 (0.00) | 0.00 (0.00) | **0.55 (0.03)** | 0.00 (0.00) |
| somatic | 0.00 (0.00) | 0.00 (0.00) | 0.00 (0.00) | 0.00 (0.00) | **0.54 (0.03)** |
| worries | 0.00 (0.00) | 0.00 (0.00) | 0.00 (0.00) | 0.00 (0.00) | **0.66 (0.02)** |
| unhappy | 0.00 (0.00) | 0.00 (0.00) | 0.00 (0.00) | 0.00 (0.00) | **0.70 (0.02)** |
| clingy | 0.00 (0.00) | 0.00 (0.00) | 0.00 (0.00) | 0.00 (0.00) | **0.53 (0.03)** |
| fears | 0.00 (0.00) | 0.00 (0.00) | 0.00 (0.00) | 0.00 (0.00) | **0.56 (0.03)** |
|  |  |  |  |  |  |
| **Factor Correlations** |  |  |  |  |  |
| Prosocial behaviour x Peer Problems | 0.71 (0.03) |  |  |  |  |
| Prosocial behaviour x Hyperactivity | 0.32 (0.04) |  |  |  |  |
| Prosocial behaviour x Conduct Problems | 0.61 (0.03) |  |  |  |  |
| Prosocial behaviour x Emotional Problems | 0.18 (0.04) |  |  |  |  |
| Peer Problems x Hyperactivity | 0.39 (0.04) |  |  |  |  |
| Peer Problems x Conduct Problems | 0.54 (0.03) |  |  |  |  |
| Peer Problems x Emotional Problems | 0.43 (0.04) |  |  |  |  |
| Hyperactivity x Conduct Problems | 0.67 (0.03) |  |  |  |  |
| Hyperactivity x Emotional Problems | 0.57 (0.03) |  |  |  |  |
| Conduct Problems x Emotional Problems | 0.57 (0.03) |  |  |  |  |

Note. Table reports estimates and standard errors (Estimates (SE)). The factor loadings on the theoretical SDQ factors are highlighted in bold.

Supplementary Table 30. *Unrestricted factor model of five-factor SDQ structure in LSIC Wave 8*

| **Factor Loadings** |  |  |  |  |  |
| --- | --- | --- | --- | --- | --- |
|  | *Factor 1* | *Factor 2* | *Factor 3* | *Factor 4* | *Factor 5* |
| considerate | -0.05 (0.05) | **0.50 (0.04)** | 0.08 (0.06) | -0.11 (0.04) | 0.30 (0.05) |
| shares | 0.03 (0.05) | **0.40 (0.04)** | 0.03 (0.02) | -0.19 (0.04) | 0.36 (0.04) |
| caring | 0.01 (0.00) | -0.13 (0.00) | **2.41 (1.07)** | 0.06 (0.00) | -0.02 (0.00) |
| kind | -0.10 (0.05) | **0.42 (0.05)** | 0.12 (0.07) | -0.18 (0.04) | **0.50 (0.05)** |
| helps | 0.04 (0.05) | 0.21 (0.05) | 0.09 (0.06) | -0.37 (0.04) | **0.43 (0.05)** |
| solitary | -0.06 (0.04) | -0.09 (0.04) | 0.07 (0.01) | 0.28 (0.04) | 0.37 (0.04) |
| friend | -0.10 (0.00) | -0.06 (0.00) | 0.10 (0.00) | 0.08 (0.00) | **0.80 (0.02)** |
| popular | 0.01 (0.04) | 0.11 (0.04) | 0.14 (0.04) | 0.00 (0.04) | **0.64 (0.04)** |
| bullied | 0.11 (0.04) | 0.25 (0.04) | 0.04 (0.01) | 0.36 (0.04) | 0.09 (0.04) |
| prefer adults | 0.08 (0.04) | 0.17 (0.05) | 0.04 (0.01) | 0.36 (0.04) | -0.04 (0.04) |
| restless | **0.80 (0.02)** | 0.08 (0.00) | 0.09 (0.00) | 0.02 (0.00) | -0.06 (0.00) |
| fidgety | **0.83 (0.03)** | -0.03 (0.04) | 0.07 (0.03) | 0.16 (0.03) | -0.08 (0.04) |
| distractible | **0.56 (0.03)** | 0.09 (0.04) | 0.08 (0.01) | 0.11 (0.03) | 0.03 (0.03) |
| reflective | 0.33 (0.05) | 0.07 (0.05) | -0.02 (0.03) | -0.30 (0.04) | 0.34 (0.04) |
| persistent | **0.42 (0.04)** | -0.02 (0.05) | 0.06 (0.01) | -0.22 (0.04) | 0.39 (0.03) |
| tempers | 0.19 (0.04) | **0.41 (0.04)** | 0.02 (0.01) | 0.23 (0.04) | 0.08 (0.04) |
| obedient | 0.12 (0.04) | 0.36 (0.04) | 0.03 (0.02) | -0.13 (0.04) | **0.40 (0.04)** |
| fights | 0.14 (0.04) | **0.64 (0.03)** | -0.01 (0.01) | 0.07 (0.04) | 0.12 (0.04) |
| lies | 0.10 (0.05) | **0.63 (0.04)** | 0.01 (0.03) | 0.02 (0.04) | -0.05 (0.04) |
| steals | 0.03 (0.00) | **0.61 (0.03)** | -0.05 (0.00) | -0.01 (0.00) | 0.02 (0.00) |
| somatic | 0.21 (0.05) | -0.06 (0.05) | 0.10 (0.02) | **0.54 (0.03)** | -0.08 (0.04) |
| worries | 0.24 (0.05) | -0.08 (0.05) | 0.08 (0.02) | **0.49 (0.03)** | 0.14 (0.04) |
| unhappy | 0.10 (0.00) | 0.19 (0.00) | 0.08 (0.00) | **0.54 (0.03)** | 0.18 (0.00) |
| clingy | 0.24 (0.04) | 0.09 (0.05) | 0.08 (0.02) | 0.36 (0.04) | -0.09 (0.04) |
| fears | 0.12 (0.05) | 0.05 (0.05) | 0.01 (0.03) | **0.48 (0.03)** | -0.01 (0.04) |
|  |  |  |  |  |  |
| **Factor Correlations** |  |  |  |  |  |
| Factor 1 x Factor 2 | 0.42 (0.06) |  |  |  |  |
| Factor 1 x Factor 3 | -0.02 (0.05) |  |  |  |  |
| Factor 1 x Factor 4 | 0.23 (0.07) |  |  |  |  |
| Factor 1 x Factor 5 | 0.30 (0.05) |  |  |  |  |
| Factor 2 x Factor 3 | 0.19 (0.05) |  |  |  |  |
| Factor 3 x Factor 4 | 0.25 (0.08) |  |  |  |  |
| Factor 4 x Factor 5 | 0.23 (0.06) |  |  |  |  |
| Factor 3 x Factor 4 | -0.12 (0.02) |  |  |  |  |
| Factor 3 x Factor 5 | 0.11 (0.10) |  |  |  |  |
| Factor 4 x Factor 5 | 0.14 (0.07) |  |  |  |  |

Note. Table reports estimates and standard errors (Estimates (SE). Substantive factor loadings (>.40) are highlighted in bold.

Supplementary Table 31. *Restricted factor model of three-factor SDQ structure in LSIC Wave 8*

| **Factor Loadings** |  |  |  |
| --- | --- | --- | --- |
|  | *Prosocial behaviour* | *Internalizing* | *Externalizing* |
| considerate | **0.65 (0.02)** | 0.00 (0.00) | 0.00 (0.00) |
| shares | **0.58 (0.03)** | 0.00 (0.00) | 0.00 (0.00) |
| caring | **0.74 (0.02)** | 0.00 (0.00) | 0.00 (0.00) |
| kind | **0.78 (0.02)** | 0.00 (0.00) | 0.00 (0.00) |
| helps | **0.53 (0.03)** | 0.00 (0.00) | 0.00 (0.00) |
| solitary | 0.00 (0.00) | **0.30 (0.03)** | 0.00 (0.00) |
| friend | 0.00 (0.00) | **0.36 (0.03)** | 0.00 (0.00) |
| popular | 0.00 (0.00) | **0.42 (0.03)** | 0.00 (0.00) |
| bullied | 0.00 (0.00) | **0.56 (0.03)** | 0.00 (0.00) |
| prefer adults | 0.00 (0.00) | **0.41 (0.03)** | 0.00 (0.00) |
| somatic | 0.00 (0.00) | **0.49 (0.03)** | 0.00 (0.00) |
| worries | 0.00 (0.00) | **0.59 (0.03)** | 0.00 (0.00) |
| unhappy | 0.00 (0.00) | **0.73 (0.02)** | 0.00 (0.00) |
| clingy | 0.00 (0.00) | **0.48 (0.03)** | 0.00 (0.00) |
| fears | 0.00 (0.00) | **0.49 (0.03)** | 0.00 (0.00) |
| restless | 0.00 (0.00) | 0.00 (0.00) | **0.71 (0.02)** |
| fidgety | 0.00 (0.00) | 0.00 (0.00) | **0.67 (0.02)** |
| distractible | 0.00 (0.00) | 0.00 (0.00) | **0.63 (0.02)** |
| reflective | 0.00 (0.00) | 0.00 (0.00) | **0.38 (0.03)** |
| persistent | 0.00 (0.00) | 0.00 (0.00) | **0.47 (0.03)** |
| tempers | 0.00 (0.00) | 0.00 (0.00) | **0.62 (0.02)** |
| obedient | 0.00 (0.00) | 0.00 (0.00) | **0.53 (0.03)** |
| fights | 0.00 (0.00) | 0.00 (0.00) | **0.70 (0.02)** |
| lies | 0.00 (0.00) | 0.00 (0.00) | **0.58 (0.02)** |
| steals | 0.00 (0.00) | 0.00 (0.00) | **0.49 (0.03)** |
| **Factor Correlations** |  |  |  |
| Prosocial behaviour x Internalizing | 0.36 (0.04) |  |  |
| Prosocial behaviour x Externalizing | 0.53 (0.03) |  |  |
| Internalizing x Externalizing | 0.71 (0.02) |  |  |

Note. Table reports estimates and standard errors (Estimates (SE)). The factor loadings on the theoretical SDQ factors are highlighted in bold.

Supplementary Table 32. *Unrestricted factor model of three-factor SDQ structure in LSIC Wave 8*

| **Factor Loadings** |  |  |  |
| --- | --- | --- | --- |
|  | *Factor 1* | *Factor 2* | *Factor 3* |
| considerate | **0.56 (0.03)** | 0.08 (0.04) | 0.14 (0.04) |
| shares | **0.51 (0.03)** | 0.17 (0.04) | 0.03 (0.04) |
| caring | **0.72 (0.02)** | -0.04 (0.04) | -0.01 (0.04) |
| kind | **0.75 (0.02)** | 0.02 (0.00) | 0.06 (0.00) |
| helps | **0.56 (0.03)** | 0.16 (0.04) | -0.24 (0.04) |
| solitary | 0.19 (0.04) | -0.08 (0.04) | 0.28 (0.04) |
| friend | **0.57 (0.03)** | -0.05 (0.04) | 0.14 (0.04) |
| popular | **0.60 (0.03)** | 0.07 (0.04) | 0.13 (0.04) |
| bullied | 0.09 (0.04) | 0.14 (0.04) | **0.48 (0.03)** |
| adults | -0.01 (0.04) | 0.06 (0.04) | **0.43 (0.04)** |
| restless | -0.04 (0.00) | **0.82 (0.02)** | 0.04 (0.00) |
| fidgety | -0.16 (0.03) | **0.80 (0.03)** | 0.14 (0.03) |
| distractible | 0.01 (0.03) | **0.58 (0.03)** | 0.14 (0.04) |
| reflective | 0.26 (0.03) | **0.42 (0.04)** | -0.22 (0.04) |
| persistent | 0.32 (0.03) | **0.46 (0.04)** | -0.16 (0.04) |
| tempers | 0.16 (0.03) | 0.29 (0.04) | **0.40 (0.03)** |
| obedient | **0.52 (0.03)** | 0.23 (0.04) | 0.08 (0.04) |
| fights | 0.30 (0.03) | 0.32 (0.03) | 0.32 (0.03) |
| lies | 0.19 (0.03) | 0.29 (0.04) | 0.25 (0.04) |
| steals | 0.19 (0.03) | 0.22 (0.04) | 0.22 (0.04) |
| somatic | -0.17 (0.04) | 0.12 (0.04) | **0.51 (0.03)** |
| worries | -0.01 (0.04) | 0.16 (0.04) | **0.48 (0.03)** |
| unhappy | 0.14 (0.00) | 0.08 (0.00) | **0.66 (0.02)** |
| clingy | -0.07 (0.04) | 0.21 (0.04) | 0.39 (0.04) |
| fears | -0.12 (0.04) | 0.08 (0.04) | **0.50 (0.03)** |
| **Factor Correlations** |  |  |  |
| Factor 1 x Factor 2 | 0.28 (0.05) |  |  |
| Factor 1 x Factor 3 | 0.13 (0.06) |  |  |
| Factor 2 x Factor 3 | 0.37 (0.05) |  |  |

Note. Table reports estimates and standard errors (Estimates (SE)). Substantive factor loadings (>.40) are highlighted in bold.

Supplementary Table 33. *Restricted factor model of the proposed four-factor SDQ structure in LSIC Wave 8*

| **Factor Loadings** |  |  |  |  |
| --- | --- | --- | --- | --- |
|  | *Factor 1* | *Factor 2* | *Factor 3* | *Factor 4* |
| considerate | **0.62 (0.02)** | 0.00 (0.00) | 0.00 (0.00) | 0.00 (0.00) |
| shares | **0.59 (0.02)** | 0.00 (0.00) | 0.00 (0.00) | 0.00 (0.00) |
| caring | **0.68 (0.02)** | 0.00 (0.00) | 0.00 (0.00) | 0.00 (0.00) |
| kind | **0.74 (0.02)** | 0.00 (0.00) | 0.00 (0.00) | 0.00 (0.00) |
| helps | **0.56 (0.03)** | 0.00 (0.00) | 0.00 (0.00) | 0.00 (0.00) |
| obedient | **0.65 (0.02)** | 0.00 (0.00) | 0.00 (0.00) | 0.00 (0.00) |
| friend | **0.56 (0.02)** | 0.00 (0.00) | 0.00 (0.00) | 0.00 (0.00) |
| popular | **0.66 (0.02)** | 0.00 (0.00) | 0.00 (0.00) | 0.00 (0.00) |
| reflective | **0.41 (0.03)** | 0.00 (0.00) | 0.00 (0.00) | 0.00 (0.00) |
| persistent | **0.50 (0.03)** | 0.00 (0.00) | 0.00 (0.00) | 0.00 (0.00) |
| solitary | 0.00 (0.00) | **0.21 (0.03)** | 0.00 (0.00) | 0.00 (0.00) |
| bullied | 0.00 (0.00) | **0.54 (0.03)** | 0.00 (0.00) | 0.00 (0.00) |
| adults | 0.00 (0.00) | **0.36 (0.03)** | 0.00 (0.00) | 0.00 (0.00) |
| tempers | 0.00 (0.00) | **0.65 (0.02)** | 0.00 (0.00) | 0.00 (0.00) |
| fights | 0.00 (0.00) | **0.79 (0.02)** | 0.00 (0.00) | 0.00 (0.00) |
| steals | 0.00 (0.00) | **0.56 (0.03)** | 0.00 (0.00) | 0.00 (0.00) |
| lies | 0.00 (0.00) | **0.63 (0.02)** | 0.00 (0.00) | 0.00 (0.00) |
| unhappy | 0.00 (0.00) | 0.00 (0.00) | **0.69 (0.02)** | 0.00 (0.00) |
| clingy | 0.00 (0.00) | 0.00 (0.00) | **0.55 (0.03)** | 0.00 (0.00) |
| fears | 0.00 (0.00) | 0.00 (0.00) | **0.57 (0.03)** | 0.00 (0.00) |
| worries | 0.00 (0.00) | 0.00 (0.00) | **0.64 (0.02)** | 0.00 (0.00) |
| somatic | 0.00 (0.00) | 0.00 (0.00) | **0.54 (0.03)** | 0.00 (0.00) |
| restless | 0.00 (0.00) | 0.00 (0.00) | 0.00 (0.00) | **0.83 (0.02)** |
| fidgety | 0.00 (0.00) | 0.00 (0.00) | 0.00 (0.00) | **0.84 (0.02)** |
| distractible | 0.00 (0.00) | 0.00 (0.00) | 0.00 (0.00) | **0.65 (0.02)** |
|  |  |  |  |  |
| **Factor Correlations** |  |  |  |  |
| Factor 1 x Factor 2 | 0.57 (0.03) |  |  |  |
| Factor 1 x Factor 3 | 0.26 (0.04) |  |  |  |
| Factor 1 x Factor 4 | 0.37 (0.03) |  |  |  |
| Factor 2 x Factor 3 | 0.64 (0.03) |  |  |  |
| Factor 2 x Factor 4 | 0.66 (0.03) |  |  |  |
| Factor 3 x Factor 4 | 0.60 (0.03) |  |  |  |

Note. Table reports estimates and standard errors (Estimates (SE)). The factor loadings on the theoretical SDQ factors are highlighted in bold.

Supplementary Table 34. *Restricted factor model of five-factor SDQ structure in LSIC Wave 10B*

| **Factor Loadings** |  |  |  |  |  |
| --- | --- | --- | --- | --- | --- |
|  | *Prosocial behaviour* | *Peer Problems* | *Hyperactivity* | *Conduct Problems* | *Emotional Problems* |
| considerate | **0.71 (0.02)** | 0.00 (0.00) | 0.00 (0.00) | 0.00 (0.00) | 0.00 (0.00) |
| shares | **0.72 (0.02)** | 0.00 (0.00) | 0.00 (0.00) | 0.00 (0.00) | 0.00 (0.00) |
| caring | **0.76 (0.02)** | 0.00 (0.00) | 0.00 (0.00) | 0.00 (0.00) | 0.00 (0.00) |
| kind | **0.76 (0.02)** | 0.00 (0.00) | 0.00 (0.00) | 0.00 (0.00) | 0.00 (0.00) |
| helps | **0.61 (0.03)** | 0.00 (0.00) | 0.00 (0.00) | 0.00 (0.00) | 0.00 (0.00) |
| solitary | 0.00 (0.00) | **0.27 (0.04)** | 0.00 (0.00) | 0.00 (0.00) | 0.00 (0.00) |
| friend | 0.00 (0.00) | **0.71 (0.02)** | 0.00 (0.00) | 0.00 (0.00) | 0.00 (0.00) |
| popular | 0.00 (0.00) | **0.85 (0.02)** | 0.00 (0.00) | 0.00 (0.00) | 0.00 (0.00) |
| bullied | 0.00 (0.00) | **0.31 (0.04)** | 0.00 (0.00) | 0.00 (0.00) | 0.00 (0.00) |
| prefer adults | 0.00 (0.00) | **0.21 (0.04)** | 0.00 (0.00) | 0.00 (0.00) | 0.00 (0.00) |
| restless | 0.00 (0.00) | 0.00 (0.00) | **0.80 (0.02)** | 0.00 (0.00) | 0.00 (0.00) |
| fidgety | 0.00 (0.00) | 0.00 (0.00) | **0.85 (0.02)** | 0.00 (0.00) | 0.00 (0.00) |
| distractible | 0.00 (0.00) | 0.00 (0.00) | **0.76 (0.02)** | 0.00 (0.00) | 0.00 (0.00) |
| reflective | 0.00 (0.00) | 0.00 (0.00) | **0.35 (0.04)** | 0.00 (0.00) | 0.00 (0.00) |
| persistent | 0.00 (0.00) | 0.00 (0.00) | **0.51 (0.03)** | 0.00 (0.00) | 0.00 (0.00) |
| tempers | 0.00 (0.00) | 0.00 (0.00) | 0.00 (0.00) | **0.69 (0.02)** | 0.00 (0.00) |
| obedient | 0.00 (0.00) | 0.00 (0.00) | 0.00 (0.00) | **0.48 (0.03)** | 0.00 (0.00) |
| fights | 0.00 (0.00) | 0.00 (0.00) | 0.00 (0.00) | **0.81 (0.02)** | 0.00 (0.00) |
| lies | 0.00 (0.00) | 0.00 (0.00) | 0.00 (0.00) | **0.75 (0.02)** | 0.00 (0.00) |
| steals | 0.00 (0.00) | 0.00 (0.00) | 0.00 (0.00) | **0.60 (0.03)** | 0.00 (0.00) |
| somatic | 0.00 (0.00) | 0.00 (0.00) | 0.00 (0.00) | 0.00 (0.00) | **0.55 (0.03)** |
| worries | 0.00 (0.00) | 0.00 (0.00) | 0.00 (0.00) | 0.00 (0.00) | **0.65 (0.03)** |
| unhappy | 0.00 (0.00) | 0.00 (0.00) | 0.00 (0.00) | 0.00 (0.00) | **0.74 (0.02)** |
| clingy | 0.00 (0.00) | 0.00 (0.00) | 0.00 (0.00) | 0.00 (0.00) | **0.68 (0.02)** |
| fears | 0.00 (0.00) | 0.00 (0.00) | 0.00 (0.00) | 0.00 (0.00) | **0.66 (0.03)** |
|  |  |  |  |  |  |
| **Factor Correlations** |  |  |  |  |  |
| Prosocial behaviour x Peer Problems | 0.84 (0.02) |  |  |  |  |
| Prosocial behaviour x Hyperactivity | 0.35 (0.04) |  |  |  |  |
| Prosocial behaviour x Conduct Problems | 0.49 (0.04) |  |  |  |  |
| Prosocial behaviour x Emotional Problems | 0.25 (0.04) |  |  |  |  |
| Peer Problems x Hyperactivity | 0.35 (0.04) |  |  |  |  |
| Peer Problems x Conduct Problems | 0.39 (0.04) |  |  |  |  |
| Peer Problems x Emotional Problems | 0.42 (0.04) |  |  |  |  |
| Hyperactivity x Conduct Problems | 0.71 (0.03) |  |  |  |  |
| Hyperactivity x Emotional Problems | 0.70 (0.03) |  |  |  |  |
| Conduct Problems x Emotional Problems | 0.73 (0.03) |  |  |  |  |

Note. Table reports estimates and standard errors (Estimates (SE)). The factor loadings on the theoretical SDQ factors are highlighted in bold.

Supplementary Table 35. *Unrestricted factor model of five-factor SDQ structure in LSIC Wave 10B*

| **Factor Loadings** |  |  |  |  |  |
| --- | --- | --- | --- | --- | --- |
|  | *Factor 1* | *Factor 2* | *Factor 3* | *Factor 4* | *Factor 5* |
| considerate | 0.26 (0.04) | **0.47 (0.04)** | 0.01 (0.04) | -0.03 (0.05) | 0.31 (0.03) |
| shares | 0.12 (0.04) | **0.56 (0.03)** | 0.04 (0.05) | 0.00 (0.05) | 0.25 (0.03) |
| caring | 0.08 (0.00) | **0.60 (0.03)** | -0.06 (0.00) | 0.07 (0.00) | 0.29 (0.01) |
| kind | 0.01 (0.04) | **0.59 (0.03)** | -0.02 (0.05) | 0.07 (0.05) | 0.30 (0.03) |
| helps | -0.11 (0.05) | **0.56 (0.04)** | 0.22 (0.05) | -0.09 (0.05) | 0.15 (0.04) |
| solitary | -0.06 (0.05) | 0.15 (0.05) | -0.08 (0.05) | **0.49 (0.05)** | 0.07 (0.04) |
| friend | -0.20 (0.04) | 0.38 (0.04) | 0.04 (0.04) | 0.22 (0.05) | **0.40 (0.03)** |
| popular | -0.05 (0.00) | 0.00 (0.00) | 0.03 (0.00) | -0.05 (0.00) | **1.10 (0.04)** |
| bullied | **0.48 (0.04)** | -0.16 (0.05) | 0.10 (0.04) | 0.12 (0.05) | 0.25 (0.04) |
| prefer adults | 0.38 (0.05) | -0.21 (0.05) | -0.16 (0.05) | 0.25 (0.05) | 0.25 (0.05) |
| restless | 0.10 (0.04) | 0.00 (0.04) | **0.60 (0.04)** | 0.27 (0.04) | -0.05 (0.03) |
| fidgety | 0.17 (0.01) | -0.12 (0.00) | **0.66 (0.02)** | 0.19 (0.01) | 0.05 (0.00) |
| distractible | 0.19 (0.04) | -0.11 (0.04) | **0.63 (0.03)** | 0.15 (0.04) | 0.03 (0.03) |
| reflective | -0.09 (0.06) | 0.37 (0.05) | **0.52 (0.05)** | -0.19 (0.06) | 0.06 (0.05) |
| persistent | -0.09 (0.05) | 0.22 (0.05) | **0.57 (0.04)** | -0.05 (0.05) | 0.23 (0.04) |
| tempers | 0.28 (0.04) | 0.17 (0.04) | 0.23 (0.04) | 0.35 (0.04) | -0.03 (0.03) |
| obedient | 0.15 (0.05) | **0.46 (0.04)** | 0.29 (0.05) | -0.1 (0.05) | 0.16 (0.04) |
| fights | **0.52 (0.03)** | 0.22 (0.04) | 0.18 (0.04) | 0.15 (0.04) | 0.11 (0.03) |
| lies | **0.71 (0.03)** | 0.04 (0.04) | 0.14 (0.04) | 0.10 (0.04) | 0.00 (0.03) |
| steals | **0.82 (0.02)** | 0.03 (0.00) | 0.02 (0.00) | -0.06 (0.00) | -0.08 (0.00) |
| somatic | 0.08 (0.05) | -0.05 (0.05) | 0.09 (0.05) | **0.53 (0.04)** | -0.07 (0.04) |
| worries | -0.07 (0.00) | -0.09 (0.00) | 0.18 (0.01) | **0.62 (0.03)** | 0.12 (0.00) |
| unhappy | 0.24 (0.04) | 0.03 (0.04) | 0.19 (0.04) | **0.42 (0.04)** | 0.22 (0.03) |
| clingy | 0.12 (0.05) | 0.03 (0.04) | 0.15 (0.05) | **0.54 (0.04)** | 0.03 (0.04) |
| fears | 0.22 (0.05) | -0.16 (0.05) | 0.06 (0.05) | **0.47 (0.04)** | 0.11 (0.04) |
|  |  |  |  |  |  |
| **Factor Correlations** |  |  |  |  |  |
| Factor 1 x Factor 2 | 0.05 (0.07) |  |  |  |  |
| Factor 1 x Factor 3 | 0.29 (0.07) |  |  |  |  |
| Factor 1 x Factor 4 | 0.38 (0.06) |  |  |  |  |
| Factor 1 x Factor 5 | 0.14 (0.04) |  |  |  |  |
| Factor 2 x Factor 3 | 0.25 (0.07) |  |  |  |  |
| Factor 3 x Factor 4 | 0.01 (0.08) |  |  |  |  |
| Factor 4 x Factor 5 | 0.35 (0.06) |  |  |  |  |
| Factor 3 x Factor 4 | 0.32 (0.08) |  |  |  |  |
| Factor 3 x Factor 5 | 0.19 (0.05) |  |  |  |  |
| Factor 4 x Factor 5 | 0.15 (0.06) |  |  |  |  |

Note. Table reports estimates and standard errors (Estimates (SE). Substantive factor loadings (>.40) are highlighted in bold.

Supplementary Table 36. *Restricted factor model of three-factor SDQ structure in LSIC Wave 10B*

| **Factor Loadings** |  |  |  |
| --- | --- | --- | --- |
|  | *Prosocial behaviour* | *Internalizing* | *Externalizing* |
| considerate | **0.69 (0.02)** | 0.00 (0.00) | 0.00 (0.00) |
| shares | **0.73 (0.02)** | 0.00 (0.00) | 0.00 (0.00) |
| caring | **0.76 (0.02)** | 0.00 (0.00) | 0.00 (0.00) |
| kind | **0.76 (0.02)** | 0.00 (0.00) | 0.00 (0.00) |
| helps | **0.63 (0.03)** | 0.00 (0.00) | 0.00 (0.00) |
| solitary | 0.00 (0.00) | **0.36 (0.04)** | 0.00 (0.00) |
| friend | 0.00 (0.00) | **0.31 (0.04)** | 0.00 (0.00) |
| popular | 0.00 (0.00) | **0.38 (0.04)** | 0.00 (0.00) |
| bullied | 0.00 (0.00) | **0.57 (0.03)** | 0.00 (0.00) |
| prefer adults | 0.00 (0.00) | **0.41 (0.03)** | 0.00 (0.00) |
| somatic | 0.00 (0.00) | **0.52 (0.03)** | 0.00 (0.00) |
| worries | 0.00 (0.00) | **0.63 (0.03)** | 0.00 (0.00) |
| unhappy | 0.00 (0.00) | **0.75 (0.02)** | 0.00 (0.00) |
| clingy | 0.00 (0.00) | **0.66 (0.02)** | 0.00 (0.00) |
| fears | 0.00 (0.00) | **0.62 (0.03)** | 0.00 (0.00) |
| restless | 0.00 (0.00) | 0.00 (0.00) | **0.70 (0.02)** |
| fidgety | 0.00 (0.00) | 0.00 (0.00) | **0.74 (0.02)** |
| distractible | 0.00 (0.00) | 0.00 (0.00) | **0.72 (0.02)** |
| reflective | 0.00 (0.00) | 0.00 (0.00) | **0.36 (0.04)** |
| persistent | 0.00 (0.00) | 0.00 (0.00) | **0.50 (0.03)** |
| tempers | 0.00 (0.00) | 0.00 (0.00) | **0.67 (0.02)** |
| obedient | 0.00 (0.00) | 0.00 (0.00) | **0.48 (0.03)** |
| fights | 0.00 (0.00) | 0.00 (0.00) | **0.73 (0.02)** |
| lies | 0.00 (0.00) | 0.00 (0.00) | **0.68 (0.02)** |
| steals | 0.00 (0.00) | 0.00 (0.00) | **0.51 (0.03)** |
| **Factor Correlations** |  |  |  |
| Prosocial behaviour x Internalizing | 0.36 (0.04) |  |  |
| Prosocial behaviour x Externalizing | 0.47 (0.04) |  |  |
| Internalizing x Externalizing | 0.81 (0.02) |  |  |

Note. Table reports estimates and standard errors (Estimates (SE)). The factor loadings on the theoretical SDQ factors are highlighted in bold.

Supplementary Table 37. *Unrestricted factor model of three-factor SDQ structure in LSIC Wave 10B*

| **Factor Loadings** |  |  |  |
| --- | --- | --- | --- |
|  | *Factor 1* | *Factor 2* | *Factor 3* |
| considerate | **0.68 (0.03)** | 0.00 (0.05) | 0.20 (0.04) |
| shares | **0.70 (0.03)** | 0.06 (0.05) | 0.05 (0.04) |
| caring | **0.74 (0.02)** | -0.01 (0.05) | 0.07 (0.04) |
| kind | **0.73 (0.02)** | 0.03 (0.05) | 0.01 (0.04) |
| helps | **0.60 (0.03)** | 0.24 (0.05) | -0.28 (0.05) |
| solitary | 0.14 (0.04) | 0.08 (0.05) | 0.24 (0.04) |
| friend | **0.62 (0.03)** | 0.06 (0.05) | 0.00 (0.04) |
| popular | **0.74 (0.02)** | -0.04 (0.00) | 0.15 (0.00) |
| bullied | 0.09 (0.04) | 0.11 (0.05) | **0.56 (0.04)** |
| adults | 0.02 (0.00) | -0.12 (0.00) | **0.61 (0.03)** |
| restless | -0.06 (0.04) | **0.72 (0.03)** | 0.13 (0.04) |
| fidgety | -0.07 (0.00) | **0.74 (0.02)** | 0.17 (0.01) |
| distractible | -0.06 (0.04) | **0.70 (0.03)** | 0.17 (0.04) |
| reflective | 0.36 (0.04) | **0.50 (0.05)** | -0.36 (0.05) |
| persistent | 0.37 (0.04) | **0.58 (0.04)** | -0.22 (0.05) |
| tempers | 0.10 (0.04) | 0.39 (0.04) | 0.37 (0.04) |
| obedient | **0.53 (0.03)** | 0.29 (0.04) | -0.02 (0.04) |
| fights | 0.29 (0.04) | 0.28 (0.04) | **0.48 (0.04)** |
| lies | 0.06 (0.04) | 0.25 (0.05) | **0.59 (0.03)** |
| steals | 0.01 (0.05) | 0.10 (0.05) | **0.58 (0.04)** |
| somatic | -0.14 (0.04) | 0.27 (0.05) | 0.36 (0.04) |
| worries | -0.03 (0.04) | 0.35 (0.05) | 0.32 (0.04) |
| unhappy | 0.18 (0.04) | 0.32 (0.04) | **0.47 (0.04)** |
| clingy | 0.03 (0.04) | 0.33 (0.05) | **0.40 (0.04)** |
| fears | -0.07 (0.04) | 0.20 (0.05) | **0.49 (0.04)** |
| **Factor Correlations** |  |  |  |
| Factor 1 x Factor 2 | 0.28 (0.06) |  |  |
| Factor 1 x Factor 3 | 0.07 (0.07) |  |  |
| Factor 2 x Factor 3 | 0.38 (0.07) |  |  |

Note. Table reports estimates and standard errors (Estimates (SE)). Substantive factor loadings (>.40) are highlighted in bold.

Supplementary Table 38. *Restricted factor model of the proposed four-factor SDQ structure in LSIC Wave 10B*

| **Factor Loadings** |  |  |  |  |
| --- | --- | --- | --- | --- |
|  | *Factor 1* | *Factor 2* | *Factor 3* | *Factor 4* |
| considerate | **0.70 (0.02)** | 0.00 (0.00) | 0.00 (0.00) | 0.00 (0.00) |
| shares | **0.72 (0.02)** | 0.00 (0.00) | 0.00 (0.00) | 0.00 (0.00) |
| caring | **0.73 (0.02)** | 0.00 (0.00) | 0.00 (0.00) | 0.00 (0.00) |
| kind | **0.73 (0.02)** | 0.00 (0.00) | 0.00 (0.00) | 0.00 (0.00) |
| helps | **0.64 (0.02)** | 0.00 (0.00) | 0.00 (0.00) | 0.00 (0.00) |
| obedient | **0.66 (0.02)** | 0.00 (0.00) | 0.00 (0.00) | 0.00 (0.00) |
| friend | **0.64 (0.02)** | 0.00 (0.00) | 0.00 (0.00) | 0.00 (0.00) |
| popular | **0.73 (0.02)** | 0.00 (0.00) | 0.00 (0.00) | 0.00 (0.00) |
| reflective | **0.52 (0.03)** | 0.00 (0.00) | 0.00 (0.00) | 0.00 (0.00) |
| persistent | **0.59 (0.03)** | 0.00 (0.00) | 0.00 (0.00) | 0.00 (0.00) |
| solitary | 0.00 (0.00) | **0.28 (0.04)** | 0.00 (0.00) | 0.00 (0.00) |
| bullied | 0.00 (0.00) | **0.61 (0.03)** | 0.00 (0.00) | 0.00 (0.00) |
| adults | 0.00 (0.00) | **0.42 (0.03)** | 0.00 (0.00) | 0.00 (0.00) |
| tempers | 0.00 (0.00) | **0.66 (0.02)** | 0.00 (0.00) | 0.00 (0.00) |
| fights | 0.00 (0.00) | **0.77 (0.02)** | 0.00 (0.00) | 0.00 (0.00) |
| steals | 0.00 (0.00) | **0.65 (0.03)** | 0.00 (0.00) | 0.00 (0.00) |
| lies | 0.00 (0.00) | **0.79 (0.02)** | 0.00 (0.00) | 0.00 (0.00) |
| unhappy | 0.00 (0.00) | 0.00 (0.00) | **0.73 (0.02)** | 0.00 (0.00) |
| clingy | 0.00 (0.00) | 0.00 (0.00) | **0.69 (0.02)** | 0.00 (0.00) |
| fears | 0.00 (0.00) | 0.00 (0.00) | **0.66 (0.03)** | 0.00 (0.00) |
| worries | 0.00 (0.00) | 0.00 (0.00) | **0.64 (0.03)** | 0.00 (0.00) |
| somatic | 0.00 (0.00) | 0.00 (0.00) | **0.55 (0.03)** | 0.00 (0.00) |
| restless | 0.00 (0.00) | 0.00 (0.00) | 0.00 (0.00) | **0.81 (0.02)** |
| fidgety | 0.00 (0.00) | 0.00 (0.00) | 0.00 (0.00) | **0.86 (0.02)** |
| distractible | 0.00 (0.00) | 0.00 (0.00) | 0.00 (0.00) | **0.74 (0.02)** |
|  |  |  |  |  |
| **Factor Correlations** |  |  |  |  |
| Factor 1 x Factor 2 | 0.40 (0.04) |  |  |  |
| Factor 1 x Factor 3 | 0.30 (0.04) |  |  |  |
| Factor 1 x Factor 4 | 0.35 (0.04) |  |  |  |
| Factor 2 x Factor 3 | 0.77 (0.02) |  |  |  |
| Factor 2 x Factor 4 | 0.69 (0.03) |  |  |  |
| Factor 3 x Factor 4 | 0.71 (0.03) |  |  |  |

Note. Table reports estimates and standard errors (Estimates (SE)). The factor loadings on the theoretical SDQ factors are highlighted in bold.
